# SLC7A11 suppresses paclitaxel resistance and improves survival via the KLF2 regulatory axis associated with autophagy in ovarian cancer

**DOI:** 10.1186/s13062-026-00909-x

**Published:** 2026-07-22

**Authors:** Xiaoying Chen, Yao Ke, Shanzhou Xie, Yulin Guo, Yuting Su, Xuemei Sun, Lizhou Shi, Yue Yu, Shunmei Lei, Dan Wei, Han Zhang, Fuqiang Yin, Xia Liu

**Affiliations:** 1 Key Laboratory of Longevity and Aging-related Diseases of Chinese Ministry of Education, Institute of Neuroscience and Guangxi Key Laboratory of Brain Science, School of Basic Medical Sciences, Guangxi Medical University, 22 Shuangyong Rd, Nanning, Guangxi 530021 P. R. China; 2 Life Sciences Institute, Guangxi Medical University, 22 Shuangyong Rd, Nanning, Guangxi 530021 P. R. China; 3 Medical Science Laboratory, Children’s Hospital, Maternal and Child Health Hospital of Guangxi Zhuang Autonomous Region, 59 Xiangzhu Rd, Nanning, Guangxi 530003 P. R. China; 4 Key Laboratory of High-Incidence-Tumor Prevention and Treatment, Guangxi Medical University, Ministry of Education, 22 Shuangyong Rd, Nanning, Guangxi 530021 P. R. China; 5 Guangxi Health Commission Key Laboratory of Basic Research on Brain Function and Disease, Guangxi Medical University, 22 Shuangyong Rd, Nanning, Guangxi 530021 P. R. China; 6 Key Laboratory of Human Development and Disease Research, (Guangxi Medical University), Education Department of Guangxi Zhuang Autonomous Region, Guangxi Medical University, 22 Shuangyong Rd, Nanning, Guangxi 530021 P. R. China

**Keywords:** SLC7A11, Ovarian cancer, Paclitaxel resistance, Prognosis, Autophagy, KLF2

## Abstract

**Background:**

SLC7A11 is well-established as a key mediator of ferroptosis. However, its functional role in regulating autophagy, particularly in the context of ovarian cancer (OC) chemoresistance, remains poorly characterized. Our preliminary results suggested that low SLC7A11 expression is associated with paclitaxel resistance in OC, likely through inhibition of autophagy. Despite this initial observation, systematic functional investigations into the role of SLC7A11 in autophagy regulation and OC chemoresistance are lacking, and the underlying molecular mechanisms remain unclear. Therefore, this study aims to elucidate how SLC7A11 mediates chemoresistance in OC through the regulation of autophagy.

**Methods:**

The effects of SLC7A11 modulation were assessed in paclitaxel-resistant and parental OC cells using functional assays. Autophagy was evaluated via transmission electron microscopy, immunofluorescence, and Western blotting. Autophagy was inhibited using 3-methyladenine or LC3B knockdown. A subcutaneous xenograft model was used for in vivo validation. Clinical relevance was analyzed using patient tissues. Proteomics, bioinformatics, molecular, and CCK-8 assays were integrated to investigate the SLC7A11/KLF2-autophagy axis.

**Results:**

SLC7A11 knockdown enhanced the viability of paclitaxel-resistant OC cells (HeyA8-R) following paclitaxel treatment. In contrast, SLC7A11 overexpression sensitized both HeyA8-R cells and their parental HeyA8 cells to paclitaxel, as evidenced by reduced viability, increased intracellular paclitaxel accumulation, and elevated DNA damage. Moreover, SLC7A11 overexpression augmented autophagy-associated activity, and inhibition of autophagy partially attenuated SLC7A11-mediated sensitization to paclitaxel. In vivo studies showed that SLC7A11 suppressed tumor growth and overcame paclitaxel resistance in OC, accompanied by upregulated autophagy-related markers (LC3B, LC3B-II/I, and LAMP1). Mechanistically, SLC7A11 negatively regulated KLF2, a key oncogenic autophagy regulator, and the SLC7A11/KLF2 regulatory axis was associated with LC3B expression and autophagy-associated activity, which may contribute to paclitaxel sensitivity in OC. Clinically, low SLC7A11 expression correlated with chemoresistance and shorter survival in OC.

**Conclusions:**

Our findings underscore the significance of the SLC7A11/KLF2-autophagy regulatory axis in OC paclitaxel resistance. They also suggest that SLC7A11 may serve as a prognostic biomarker and therapeutic target for overcoming OC chemoresistance.

**Supplementary Information:**

The online version contains supplementary material available at https://doi.org/10.1186/s13062-026-00909-x.

## Introduction

Ovarian cancer (OC) is the second leading cause of gynecologic cancer death worldwide, with high mortality [1]. According to the latest global cancer statistics, there were 324,398 new OC cases and 206,839 OC-related deaths in 2022 alone [2]. Cytoreductive surgery combined with paclitaxel-based chemotherapy is one of the preferred treatment strategies for OC, and patients generally achieve favorable responses in the early stage of treatment [3, 4]. However, OC is often asymptomatic in its early stages, and approximately 80% of patients are diagnosed at an advanced stage [5]. Most patients with advanced disease eventually develop drug resistance, leading to recurrence and death, resulting in a 5-year survival rate of only approximately 48% [3–5]. Therefore, elucidating the mechanisms of chemotherapy resistance in OC is essential to identify effective therapeutic targets and improve patient prognosis.

The cystine/glutamate antiporter solute carrier family 7 member 11 (SLC7A11) is a light chain subunit with transport activity in the Xc- system [6]. Multiple studies have demonstrated that SLC7A11 is involved in regulating tumor resistance to platinum [7, 8], taxanes [9–11], and adriamycin [12], and SLC7A11 functions as a tumor suppressor in certain cancer types [13, 14]. For example, knockdown of SLC7A11 renders retinoblastoma resistant to arginine deprivation [15]. SLC7A11 was significantly downregulated in adriamycin-resistant breast cancer cells [12], sorafenib-resistant liver cancer cells [16], and paclitaxel-resistant OC cells [9]. Low SLC7A11 expression enhances chemoresistance in breast cancer by inducing P-gp up-regulation [12]. Consistently, our previous study showed that low SLC7A11 expression confers drug resistance and worse survival in OC and is associated with inhibited autophagy [17, 18]. However, these findings were limited to bioinformatics analysis and Western blot validation of seven autophagy-related proteins, with functional studies confined to cellular assays.

Autophagy is a self-degradative process in which intracellular components are metabolized and recycled [19]. Targeting autophagy represents one of the promising strategies to overcome tumor chemotherapy resistance [20]. For instance, activation of autophagy increases the sensitivity of nasopharyngeal carcinoma cells to paclitaxel [21] and triple-negative breast cancer cells to doxorubicin [22]. Moreover, SLC7A11 has been shown to induce autophagic cell death in triple-negative breast cancer cells [23], hepatocellular carcinoma [24], and sut melanocytes [25]. Therefore, the mechanism by which SLC7A11-mediated autophagy regulates paclitaxel resistance in OC warrants further exploration.

Krüppel-like factor 2 (KLF2) belongs to the KLF family of transcription factors [26]. KLF2 has been identified as a negative regulator of autophagy [27]. It functions as a tumor promoter in clear cell renal cell carcinoma [28] and multiple myeloma [29, 30]. KLF2 expression is negatively correlated with tumor cell sensitivity to multiple drugs and represents a risk factor for poor prognosis across various tumor types [31]. Notably, KLF2 mutation is the most common risk factor in ovarian serous cystadenocarcinoma [31]. However, the relationship between KLF2 and SLC7A11, chemotherapy resistance, and autophagy in OC has not been investigated.

In this study, we further demonstrated that low SLC7A11 expression contributes to paclitaxel resistance and poor prognosis in OC through in vitro functional assay and clinical sample analysis. Overexpression of SLC7A11 inhibited paclitaxel resistance in OC cells in vitro and in vivo by mediating autophagy, and suppressed tumorigenicity in vivo. Furthermore, integration of proteomics, bioinformatics analysis, and in vitro functional experiments suggested that SLC7A11 negatively regulated KLF2 and may jointly modulate autophagy to suppress paclitaxel resistance in OC. These findings suggest that SLC7A11 and KLF2 represent potential targets for overcoming chemotherapy resistance and improving survival in OC.

## Methods

### Cell lines and cell culture

Human OC cell lines HeyA8 and SKOV3 were kindly provided by Prof. Fengxia Xue of Tianjin Medical University, a co-author of Yang et al., who previously characterized and utilized these cell lines [32]. Cell identity was further confirmed by morphological assessment and growth characteristics consistent with the reported profiles. Independent STR profiling was not performed in this study. Paclitaxel-resistant OC cells (HeyA8-R and SKOV3-R) were established from parental cell lines by gradual exposure to increasing concentrations of paclitaxel as previously described [17]. The resistance index (RI=IC_50_ of resistant cells / IC_50_ of parental cells) of paclitaxel was 5.42 ± 0.55 for HeyA8-R cells and 34.47 ± 4.84 for SKOV3-R cells [17]. Cells were cultured in RPMI-1640 medium (Wisent) supplemented with 10% fetal bovine serum (Wisent) and 1% penicillin-streptomycin (Solarbio) at 37℃ in a humidified atmosphere with 5% CO_2_. Cell lines were tested for mycoplasma contamination and remained mycoplasma-free throughout the study. The present study was approved by the Ethics Committee of Guangxi Medical University (No. KY20250379).

### Paraffin sections of clinical tumor tissues and clinical data

Paraffin-embedded tumor tissue sections and corresponding clinical data were obtained from the Affiliated Tumor Hospital of Guangxi Medical University. A total of 36 paraffin-embedded benign tumor tissues (uterine leiomyoma) collected from 2017 to 2021, 35 chemotherapy-sensitive OC tissues collected from 2014 to 2022 (including 31 cases of high-grade serous carcinoma, 1 case of serous carcinoma, 1 case of clear cell carcinoma, 1 case of mucinous carcinoma, and 1 case of endometrioid adenocarcinoma), and 30 chemotherapy-resistant OC tissues collected from 2014 to 2022 (including 26 cases of high-grade serous carcinoma, 1 case of serous adenocarcinoma, 1 case of moderately to poorly differentiated adenocarcinoma, 1 case of high-grade mixed seromucinous carcinoma, and 1 case of mucinous papillary cystadenocarcinoma) were retrospectively collected. The clinical characteristics of 65 patients with OC are summarized in Table S1, and detailed clinical data are provided in Table S2. Of the 65 patients, 17 had incomplete overall survival (OS) follow-up data and were excluded from OS analysis, leaving 48 patients with complete OS data. For progression-free survival (PFS) analysis, 8 patients with undocumented recurrence time (including 6 cases of uncontrolled disease and 2 cases of primary resistance) were excluded. Patients with available recurrence records but incomplete OS follow-up were additionally included, yielding 57 patients for PFS analysis. The study was approved by the Ethics Committee of Guangxi Medical University (No. KY20250379). All patients provided informed consent for the use of specimens prior to surgery.

The inclusion criteria for benign tumor group were as follows: patients diagnosed with uterine leiomyoma who had bilaterally normal ovaries and underwent hysterectomy or myomectomy. The inclusion criteria for the chemotherapy-sensitive and chemotherapy-resistant OC groups were as follows: patients who could not achieve optimal cytoreduction upon preoperative evaluation received 1–5 cycles of neoadjuvant chemotherapy followed by interval cytoreductive surgery. After surgery, patients received 4–12 cycles of paclitaxel or docetaxel plus platinum-based chemotherapy. Patients with PFS of < 6 months were defined as chemotherapy-resistant, and those with a PFS of ≥ 6 months were defined as chemotherapy-sensitive. PFS was defined as the interval from the completion of the last chemotherapy to disease recurrence.

### Real-time quantitative polymerase chain reaction (RT-qPCR)

Total RNA was extracted using TRIzol reagent (15596026CN, Invitrogen) according to the manufacturer’s instructions, as described previously [17]. Reverse transcription was performed with 1 µg of total RNA using PrimeScript™ RT reagent Kit with gDNA Eraser (Perfect Real Time) (RR047A, Takara). Quantitative PCR was carried out on the ABI 7300 System (Thermo Fisher Scientific) using PerfectStart^®^ Green qPCR SuperMix (AQ601-01-V2, TransGen Biotech). The relative expression levels of *SLC7A11*, *LC3B*, *KLF2*, *TFCP2*, and *SOX7* were calculated using the 2⁻ΔΔCt method, with *GAPDH* as the internal reference. Primer sequences are listed in Table S3. All experiments were performed with three independent biological replicates, and data are presented as mean ± SD.

### Western blotting

Protein extraction, quantification, and western blotting were performed as previously described [17]. Briefly, total protein was extracted using RIPA buffer (R0010, Solarbio), quantified by a BCA assay (P0009, Beyotime), and separated by SDS-PAGE, followed by transfer to PVDF membranes (ISEQ00010, Millipore). After blocking, the membranes were incubated overnight at 4 °C with primary antibodies. The following antibodies from Abcam were used: SLC7A11 (ab37185), KLF2 (ab236507), and TFCP2 (ab308611). The following antibodies from Cell Signalling Technology were used: p18 INK4C (#2890), p21 Waf1/Cip1 (#2947), p27 Kip1 (#3686), Cyclin B1 (#12231), Cyclin D1 (#2978), PARP (#9542), Lamin A/C (#4777), Caspase-3 (#14220), Caspase-8 (#9746), Phospho-Histone H_2_A.X (Ser139) (#9718), MDR1/ABCB1 (#13342 and #13978), UVRAG (#13115), LC3B (#3868), LAMP1 (#9091), PI3K p110a (#4249), mTOR (#2983), p-mTOR (#5536), Beclin-1 (#3495), Rab7 (#9367). The following antibodies from Immunoway were used: STX17 (YN4187), RAB33B (YN1177). The following antibodies from Hangzhou Fude Biological Technology were used: GAPDH (FD0063), β-Tubulin (FD0064). β-actin antibodies (66009-1-Ig) from Proteintech were used. After washing, membranes were incubated with HRP-conjugated secondary antibodies (anti-rabbit/anti-mouse IgG, Cell Signalling Technology) at room temperature for 1 h. Protein bands were visualized using chemiluminescent substrate reagent (34580, Thermo Fisher Scientific) and a chemiluminescence imaging system (MiniChemi 610 Plus, Sage Creation). Band intensities were quantified using ImageJ. All experiments were performed with three independent biological replicates, and data are presented as mean ± SD.

### siRNA transfection and lentivirus infection

After seeding in 96-well and 6-well plates, cells were cultured at 37℃ in a humidified incubator with 5% CO_2_. When cells had adhered and reached 35–40% confluence, transfection complexes were prepared according to the manufacturer’s instructions for the riboFECT^TM^-CP transfection reagent and siRNA products (Guangzhou RiboBio Co., Ltd., Guangzhou, China). The final concentration of siRNA transfection was 50 nmol/L. Transfected cells were used for subsequent experiments at 24–96 h post-transfection, depending on the specific experimental requirements. siRNA knockdown efficiency was confirmed by RT-qPCR and Western blotting. siRNAs targeting *SLC7A11*, *LC3B*, *KLF2*, as well as a negative control (NC) siRNA, were designed and synthesized by RiboBio.

Lentivirus infection was performed as previously described [17]. The *SLC7A11*-overexpressing lentiviral vector LV-EF1a> h*SLC7A11*-CMV> eGFP/T2A/Puro and the control lentiviral vector LV-CMV> eGFP/T2A/Puro were purchased from Cyagen Biosciences (Guangzhou, China). Non-fluorescent lentiviral particles, including *SLC7A11*-overexpressing LV-*SLC7A11* (107403-2) and the negative control CON290(Ubi-MCS-SV40-Neomycin), were purchased from Shanghai Genechem Co., Ltd. (Shanghai, China).

### CCK-8 assay and colony formation assay

Cell viability and colony-forming ability were assessed as previously described [17]. For the CCK-8 assay, cells were seeded in 96-well plates at an appropriate density (e.g., 600–3,000 cells/well, depending on the cell growth rate), and cell viability was assessed using the CCK-8 reagent (K1018, APExBIO) according to the manufacturer’s instructions. Each biological replicate included at least three technical replicates. For the colony formation assay, cells were seeded in 60 mm dishes or 6-well plates at an appropriate density (e.g., 400–600 cells/well, depending on the cell growth rate) and cultured until visible colonies formed. The colonies were then fixed with 4% paraformaldehyde fix solution (P1110, Solarbio), stained with 0.02% crystal violet (G1063, Solarbio), and counted. All experiments were performed with at least three independent biological replicates, and data are presented as mean ± SD.

### Immunofluorescence staining

*SLC7A11*-overexpressing HeyA8-R-SLC7A11 (H-R-SLC7A11) cells and control HeyA8-R-eGFP (H-R-eGFP) cells were seeded on coverslips in 24-well plates at a density of 3,500 cells/well (vehicle group) and 10,500 cells/well (drug group). Cells were then treated with 0, 15.625, or 31.25 nmol/L paclitaxel (S1150, Selleck) for 72 h. Cells were fixed, permeabilized, and blocked as previously described [33]. They were then incubated overnight at 4 °C with the following primary antibodies: Phospho-Histone H2A.X (Ser139) (dilution 1:400, #9718, Cell Signalling Technology), LC3B (dilution 1:1,000, #3868, Cell Signalling Technology), or LAMP1 (dilution 1:1,000, #9091, Cell Signalling Technology). After washing, cells were incubated with Alexa Fluor secondary antibody (dilution 1:500, A-21422, Invitrogen) for 1 h at room temperature, followed by DAPI (0100 − 20, SouthernBiotech) staining. Images were acquired using a confocal microscope (TCS SP8, Leica). All experiments were performed with three independent biological replicates, and data are presented as mean ± SD.

### High performance liquid chromatography (HPLC)

H-R, H-R-eGFP, and H-R-SLC7A11 cells were seeded at 480,000 cells/dish in five 10 cm dishes per cell line. Cells were treated with 0 or 31.25 nmol/L paclitaxel for 24–48 h. Cells were collected, and the cell pellet was washed twice with precooled ddH_2_O. The cells were then resuspended in 150 µL of HPLC-grade methanol (M116118, Aladdin). Cells were then disrupted using an ultrasonic homogenizer to release intracellular paclitaxel. After centrifugation at 12,000 rpm for 5 min at 4 °C, the supernatant was filtered through an HPLC syringe filter, and the concentration of paclitaxel was determined using an HPLC system (LC-6AD, Shimadzu, Japan). Paclitaxel standard solutions were prepared at concentrations of 1, 2, 3, 4, 8, and 16 µmol/L. The HPLC conditions were as follows: a Phenomenex C18 reversed-phase column (250 mm×4.6 mm, 5 μm), mobile phase injection ratio of methanol: water (80:20, v/v), flow rate of 1.0 mL/min, detection wavelength of 227 nm, column temperature of 33℃, and injection volume of 30 µL. The retention time of paclitaxel was approximately 5 min 6 s. The relative intracellular paclitaxel concentration was calculated from the standard curve and normalized to the total cell number. All experiments were performed with three independent biological replicates, and data are presented as mean ± SD.

### Hematoxylin-eosin staining (HE) and immunohistochemical staining (IHC)

Paraffin-embedded tissue sections were deparaffinized, rehydrated, and subjected to HE staining or IHC as previously described [34]. Briefly, for IHC, antigen retrieval was performed using pH 6.0 citrate buffer (ZLI-9065, ZSGB-BIO, Beijing, China), followed by blocking with 5% goat serum (SL038, Solarbio) and incubation with primary antibodies overnight at 4 °C. The primary antibodies used were as follows: SLC7A11 (dilution 1:50 or 1:200, ab37185, Abcam), LC3B (dilution 1:50, #3868, Cell Signaling Technology), LAMP1 (dilution 1:200, #9091, Cell Signaling Technology), and Ki-67 (dilution 1:200, #9027, Cell Signaling Technology). After washing, sections were incubated with a polymer-based detection system (mouse/rabbit enhanced polymer detection system, PV-9000, ZSGB-BIO, Beijing, China). Staining was visualized using a DAB substrate kit (G1212, Servicebio, Wuhan, China) and counterstained with hematoxylin (BSBA-4021 A, ZSGB-BIO, Beijing, China). Images were acquired using a fluorescence upright microscope (BX53, Olympus, Japan). All experiments were performed with at least three independent biological replicates, and data are presented as mean ± SD.

### Transmission electron microscopy

H-R-eGFP, H-R-SLC7A11, H-eGFP, and H-SLC7A11 cells were seeded in 10 cm cell culture dishes at a density of 1 × 10⁵ cells/dish for the vehicle group and 2 × 10⁵ cells/dish for the drug-treated groups. H-R-eGFP and H-R-SLC7A11 cells were subsequently treated with 0, 15.625, or 31.25 nmol/L paclitaxel for 72 h. H-eGFP and H-SLC7A11 cells were also subsequently treated with 0, 7.8125, or 15.625 nmol/L paclitaxel for 72 h. After treatment, cells were collected, washed with precooled 0.1 mol/L PBS (P1003, Solarbio), and pelleted by centrifugation at 1,000 rpm for 10 min, and the excess PBS was carefully removed. The cell pellets were fixed with precooled 3% glutaraldehyde (T16427, Shanghai Shangbao Biotechnology, Shanghai, China) at 4 °C for 16 h, rinsed thoroughly with 0.1 mol/L PBS, and post-fixed with 1% osmium tetroxide (115355, Chengdu Bright Chemical Technology, Chengdu, China) at 4 °C for 1–2 h. The samples were then dehydrated through an ethanol (A500737, Sangon Biotech, Shanghai, China)–acetone (179124, Merck KGaA, Darmstadt, Germany) series as follows: 1 mL of 50% ethanol at 0–4 °C for 10 min (once), 1 mL of 70% ethanol at 0–4 °C for 10 min (once), 1 mL of 90% ethanol at room temperature for 10 min (once), 1 mL of a 1:1 mixture of 90% ethanol and 90% acetone at room temperature for 10 min (once), 1 mL of 90% acetone at room temperature for 10 min (once), and 1 mL of 100% acetone at room temperature for 10 min (once). Before each reagent change, the residual reagent was carefully removed. The cell pellets were then infiltrated at 40℃ as follows: acetone: epoxy resin 618 (2:1, v/v) for 2 h, acetone: epoxy resin 618 (45347, Merck KGaA, Darmstadt, Germany) (1:2, v/v) overnight, and pure epoxy resin 618 for 2 h. For polymerization, the samples were embedded in epoxy resin 618 in embedding molds and polymerized at 40 °C for 15 h, at 48 °C for 12 h, and at 60 °C for 24 h. Ultrathin sections were cut using a LEICA EM UC7 ultramicrotome (EM UC7, Leica Microsystems, Wetzlar, Germany), double-stained with 3% uranyl acetate (DJ151, Beijing Mozhidong Biotechnology, Beijing, China) and lead citrate (DJ152, Beijing Mozhidong Biotechnology, Beijing, China), and observed using an H-7650 transmission electron microscope (H-7650, Hitachi, Tokyo, Japan). One independent biological replicate was performed, and 5–8 fields were imaged per sample.

### Xenograft experiment

A total of 112 female C-NKG mice aged 5 weeks were purchased from Cyagen Biosciences (Suzhou, China) and housed under specific pathogen-free conditions at the Experimental Animal Center of Guangxi Medical University. For the non-resistant cell xenograft model, 16 mice were randomly divided into two groups (*n* = 8 per group) using a computer-generated random number sequence. H-eGFP or H-SLC7A11 cells (5 × 10^5^) were resuspended in a mixture of 150 µL PBS (P1020, Solarbio) and 50 µL Matrigel (082706, Xiamen Mogengel) and injected subcutaneously into the right axillary region of each mouse. For the paclitaxel-resistant xenograft model, 96 mice were initially randomly assigned to two groups (*n* = 48 per group) using a computer-generated random number sequence. H-R-eGFP or H-R-SLC7A11 cells (1 × 10^7^) were resuspended in a mixture of 150 µL PBS and 50 µL Matrigel and injected subcutaneously into the right axillary region. Mouse body weight and tumor volume were measured every other day by an investigator blinded to group allocation. Tumor volume (V) was calculated using the formula: V = (length × width²)/2. When the average tumor volume in the H-R-eGFP or H-R-SLC7A11 groups reached approximately 150 mm³ on day 5, mice in each group were further randomly assigned to treatment subgroups using stratified randomization to ensure comparable baseline tumor volumes across all four subgroups, and treated intraperitoneally with paclitaxel (20 mg/kg) or an equal volume of vehicle control (200 µL) twice per week. Treatment administration could not be fully blinded due to distinguishable drug preparations; however, all outcome assessments were conducted in a blinded manner. Sample sizes (*n* = 8 for the two-group experiment; *n* = 24 per subgroup for the four-group experiment) were determined by power analysis. A minimum of 6 mice per group was estimated to provide 80% power to detect a ≥ 50% difference in final tumor weight at α = 0.05. When the tumor volume reached 1,500 mm³, mice were euthanized, and the tumors were excised and weighed by a blinded investigator. The tumor inhibition rate (TIR) was calculated as follows [21]: TIR = [(average tumor weight in the control group − average tumor weight in the experimental group) / average tumor weight in the control group] × 100%. All animal experiments were approved by the Animal Experiment Ethics Committee of Guangxi Medical University (No. 202402015).

### DIA (data independent acquisition) quantitative proteomics test

H-R-SLC7A11 and H-R-eGFP cells were seeded in 148 cm^2^ culture dishes at a density of 3.6 × 10⁵ cells/dish (vehicle group) or 7.2 × 10⁵ cells/dish (drug group). Cells were treated with 0 or 31.25 nmol/L paclitaxel for 72 h. Three independent biological replicates per condition were collected and snap-frozen in liquid nitrogen. Samples were sent on dry ice to Applied Protein Technology (Zhejiang, China) for proteomics analysis. Proteins were extracted using the sodium deoxycholate lysis method, quantified by the BCA assay, and assessed for quality by SDS-PAGE. For each sample, 100 µg of protein was subjected to DIA (data independent acquisition) analysis on an Orbitrap Astral high-resolution mass spectrometer (Thermo Fisher Scientific). Protein intensities were normalized using median normalization. Protein sequence annotation was performed using the Uniprot database (https://www.uniprot.org/). Differentially expressed proteins were further analyzed by hierarchical clustering, volcano plots, Venn diagrams, and KEGG pathway classification and enrichment analysis.

### Bioinformatics analyses

The GeneMANIA online tool (http://www.genemania.org/) was used to construct an interaction network of SLC7A11 with KLF2, TFCP2, and SOX7. Based on the GEPIA database (http://gepia.cancer-pku.cn/detail.php?gene=&clicktag=stageplot), Pearson correlation analysis was performed to evaluate the correlation between SLC7A11 expression and KLF2, TFCP2, or SOX7 expression in the TCGA ovarian serous cystadenocarcinoma cohort. ROC Plotter database (https://rocplot.org/ovarian/index) was used to analyze the association between KLF2, TFCP2, and SOX7 expression levels and platinum/taxane resistance in OC tissues. A recurrence-free survival time < 6 months was used as the cutoff to distinguish chemoresistant from chemosensitive tissues. Expression and prognostic data of the target genes in 1,815 patients with OC were downloaded from the Kaplan-Meier Plotter database (https://kmplot.com/analysis/index.php?p=service&cancer=ovar). For each gene, mRNA expression was dichotomized into high (H) and low (L) expression groups using the best cut-off value automatically determined by the database. The associations between KLF2, TFCP2, and SOX7 expression levels and overall survival (OS), progression-free survival (PFS), and post-progression survival (PPS) in patients with OC were evaluated by the Kaplan-Meier survival analysis method and the Cox proportional hazard model. Furthermore, the prognostic value of the combined expression patterns of 2 genes was evaluated using methods similar to those in previous studies [35, 36].

### Statistical analysis

Experimental data were statistically analyzed using IBM SPSS Statistics 26, GraphPad Prism 8.0, and Microsoft Excel, and are presented as mean ± SD or SEM. Student’s t-test was used for comparisons between two groups. One-way ANOVA was used for multiple group comparisons. When equal variances were assumed, Fisher’s LSD test was used for post-hoc comparisons; when equal variances were not assumed, Tamhane’s T2 test was applied. Two-way ANOVA with Bonferroni’s post-hoc test was applied for dose-response experiments and comparisons involving two factors. For proteomics data, differentially expressed proteins were initially screened using fold change > 1.5 (or < 1/1.5) and nominal *P* < 0.05 (Student’s t-test). The Benjamini-Hochberg procedure was subsequently applied to control the false discovery rate (FDR); FDR-adjusted *P* values are provided in the supplementary materials. Associations between categorical variables and *SLC7A11* were assessed by Pearson’s chi-square test or chi-square test with continuity correction, as appropriate. For survival analyses, an IHC score of 5 was used as the pre-specified cutoff. The Kaplan-Meier method with log-rank test and multivariate Cox regression were used to evaluate the prognostic value of single gene and/or combined gene expression patterns, with sensitivity analysis performed using the receiver operating characteristic (ROC)-derived optimal cutoff. Database-derived survival analyses were exploratory, with each gene-outcome association tested independently without multiplicity adjustment. ROC curve analysis was used to evaluate the predictive performance of *SLC7A11* expression for chemotherapy response. Western blot band intensities, immunofluorescence intensities, and colony areas were quantified using ImageJ. Statistical significance was defined as *P* < 0.05. ns (not significant), *P* > 0.05; *, *P* < 0.05; **, *P* < 0.01; ***, *P* < 0.001.

## Results

### SLC7A11 suppresses paclitaxel resistance in OC

#### SLC7A11 knockdown and overexpression exert opposing effects on paclitaxel resistance in OC

**Fig. 1:**
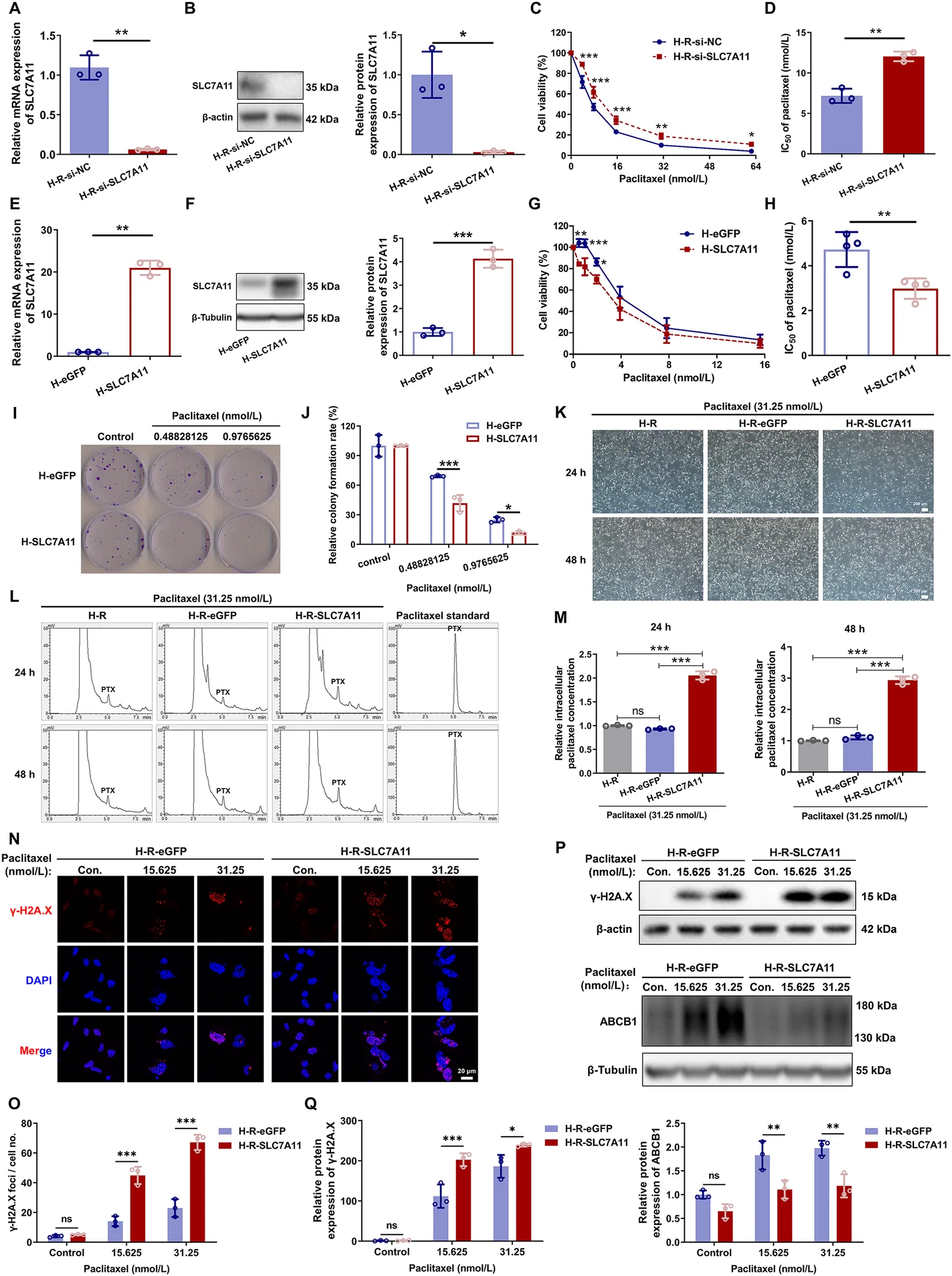
Effect of SLC7A11 knockdown or overexpression on paclitaxel resistance, intracellular drug accumulation, and DNA damage in ovarian cancer paclitaxel-resistant (HeyA8-R, H-R) or parental (HeyA8, H) cells. (**A-B**,** E-F**) The expression level of SLC7A11 in H-R-si-SLC7A11 vs. H-R-si-NC and H-SLC7A11 vs. H-eGFP was analyzed by RT-qPCR and Western blotting. (**C-D**,** G-H**) Cell viability and paclitaxel IC_50_ in H-R-si-SLC7A11 vs. H-R-si-NC and H-SLC7A11 vs. H-eGFP cells treated with a gradient of paclitaxel for 72 h, determined by CCK-8 assay. (**I-J**) Representative images and relative colony formation rates of H-SLC7A11 and H-eGFP cells treated with a gradient of paclitaxel for 9 days. (**K-M**) Intracellular paclitaxel concentration in H-R, H-R-SLC7A11, and H-R-eGFP cells treated with 31.25 nmol/L paclitaxel for 24 h and 48 h, detected by HPLC. K: representative cell images (scale bar: 200 μm); L: representative kurtosis plots; M: quantification. (**N-O**) γ-H2A.X foci in H-R-eGFP and H-R-SLC7A11 cells treated with paclitaxel for 72 h, detected by immunofluorescence. N: representative images (scale bar: 20 μm); O: quantification. (**P-Q**) ABCB1 and γ-H2A.X protein levels in H-R-eGFP and H-R-SLC7A11 cells treated with paclitaxel for 72 h, detected by Western blotting and quantified by ImageJ. Data are presented as mean ± SD. ns, *P* > 0.05; *, *P* < 0.05; **, *P* < 0.01; ***, *P* < 0.001

We previously found that SLC7A11 was downregulated in paclitaxel-resistant OC cells (HeyA8-R, H-R; SKOV3-R, S-R), and that overexpression of SLC7A11 significantly inhibited paclitaxel resistance in H-R cells [17]. To further establish its role, we performed complementary experiments. Knockdown of SLC7A11 in H-R cells (Fig. 1A-B) significantly enhanced paclitaxel resistance compared to control cells, as demonstrated by increased relative cell viability (*P* < 0.05) and increased IC₅₀ of paclitaxel (*P* < 0.01) (Fig. 1C-D). In contrast, overexpression of SLC7A11 in parental HeyA8 (H) cells (Fig. 1E-F) sensitized cells to paclitaxel compared to control cells, as evidenced by reduced relative cell viability (*P* < 0.05) and decreased IC₅₀ of paclitaxel (*P* < 0.01) (Fig. 1G-H), and decreased relative colony formation rate (*P* < 0.05) (Fig. 1I-J). Consistent results were observed in S-R cells (Fig. S1). Furthermore, SLC7A11 overexpression altered cell cycle and apoptosis proteins in H cells (Fig. S2), supporting its role in promoting cell death [17].

#### Overexpression of SLC7A11 increases intracellular paclitaxel accumulation and suppresses ABCB1 expression in H-R cells under paclitaxel treatment

Increased drug efflux is an established mechanism of paclitaxel resistance [37]. We therefore asked whether SLC7A11 overexpression sensitizes OC cells to paclitaxel by altering this process. Specifically, we assessed its impact on intracellular paclitaxel concentration and the expression of the efflux transporter ABCB1. HPLC analysis revealed no significant difference in relative intracellular paclitaxel concentration between H-R and H-R-eGFP cells after exposure to 31.25 nmol/L paclitaxel for 24–48 h (*P* > 0.05). In contrast, the relative intracellular paclitaxel concentration was significantly increased in SLC7A11-overexpressing H-R cells (H-R-SLC7A11) under the same treatment conditions (*P* < 0.001). Notably, the relative intracellular concentration was significantly higher in H-R-SLC7A11 cells at 48 h than at 24 h of paclitaxel exposure (*P* < 0.001) (Fig. 1K-M). Consistently, Western blot analysis demonstrated that ABCB1 protein expression was significantly suppressed in H-R-SLC7A11 cells treated with paclitaxel (0, 15.625, and 31.25 nmol/L) for 72 h, compared to H-R-eGFP control cells (*P* < 0.01) (Fig. 1P-Q).

#### Overexpression of SLC7A11 increases DNA damage in H-R cells under paclitaxel treatment

We next investigated whether the pro-apoptotic effect of SLC7A11 [17] involves the DNA damage response, given that paclitaxel efficacy is linked to the induction of DNA double-strand breaks [38–41] and that blocking DNA repair can enhance its cytotoxicity [42]. Immunofluorescence staining for γ-H2A.X, a marker of DNA double-strand breaks, revealed that paclitaxel treatment induced DNA damage in a dose-dependent manner in both control (H-R-eGFP) and SLC7A11-overexpressing (H-R-SLC7A11) cells (Fig. 1N-O). Notably, the number of γ-H2A.X foci per cell was significantly higher in H-R-SLC7A11 cells at all paclitaxel concentrations (*P* < 0.001) (Fig. 1N-O). These findings were corroborated by Western blot analysis. γ-H2A.X protein was undetectable in untreated cells, but it was markedly induced after treatment with 15.625 and 31.25 nmol/L paclitaxel for 72 h. Consistent with the immunofluorescence data, the upregulation of γ-H2A.X was significantly more pronounced in H-R-SLC7A11 cells than in H-R-eGFP cells (*P* < 0.05) (Fig. 1P-Q).

### SLC7A11 suppresses paclitaxel resistance in OC through regulation of autophagy

#### Overexpression of SLC7A11 increases the number of autophagosomes, lysosomes, and autolysosomes in OC cells under paclitaxel treatment

**Fig. 2:**
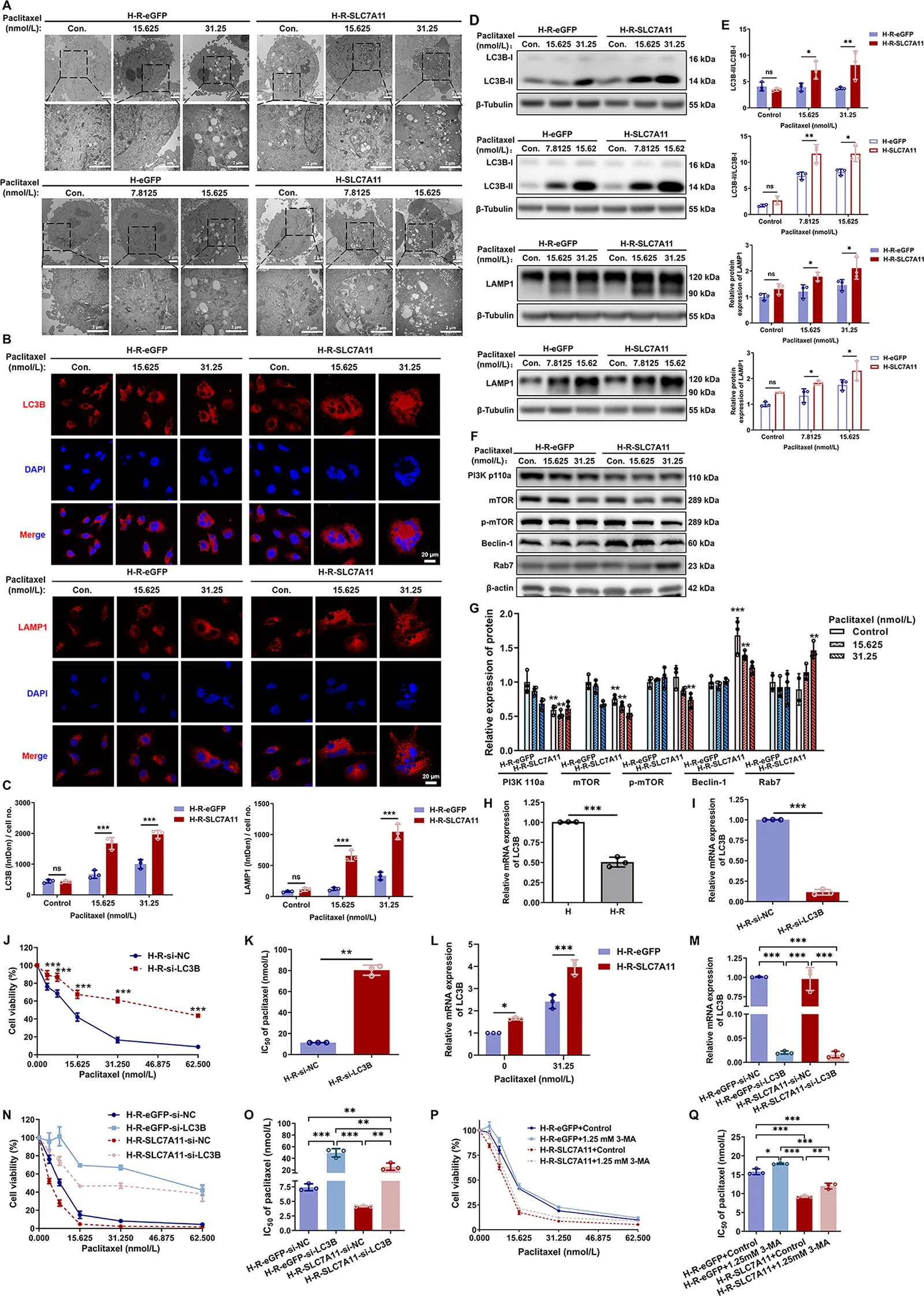
SLC7A11 regulates autophagy-related structures and proteins, and LC3B/3-MA modulates paclitaxel resistance in ovarian cancer parental (HeyA8, H) and paclitaxel-resistant (HeyA8-R, H-R) cells. (**A**) Transmission electron microscopy images of H-R-eGFP vs. H-R-SLC7A11 and H-eGFP vs. H-SLC7A11 cells treated with paclitaxel for 72 h. Scale bar: 2 μm. (**B-C**) Immunofluorescence staining (B) and quantification (C) of LC3B (red) and LAMP1 (red) in H-R-eGFP vs. H-R-SLC7A11 cells treated with paclitaxel for 72 h; nuclei stained with DAPI (blue). Scale bar: 20 μm. (**D-E**) Western blotting (D) and quantification (E) of LC3B and LAMP1 in H-R-eGFP vs. H-R-SLC7A11 and H-eGFP vs. H-SLC7A11 cells treated with paclitaxel for 72 h. (**F-G**) Western blotting (F) and quantification (G) of PI3K p110α, mTOR, p-mTOR, Beclin-1, and Rab7 in H-R-eGFP vs. H-R-SLC7A11 cells treated with paclitaxel for 72 h. (**H-I**) RT-qPCR analysis of LC3B mRNA in H vs. H-R cells (H) and H-R-si-LC3B vs. H-R-si-NC cells (I). (**J-K**) Cell viability (J) and paclitaxel IC_50_ (K) in H-R-si-LC3B and H-R-si-NC cells treated with paclitaxel for 72 h, determined by CCK-8 assay. (**L-M**) RT-qPCR analysis of LC3B mRNA in H-R-eGFP vs. H-R-SLC7A11 cells treated with 0 or 31.25 nmol/L paclitaxel for 72 h (L), and H-R-eGFP-si-LC3B, H-R-SLC7A11-si-LC3B, and respective si-NC control cells (M). (**N-O**) Cell viability (N) and paclitaxel IC_50_ (O) in H-R-eGFP-si-LC3B, H-R-SLC7A11-si-LC3B, and respective si-NC control cells treated with paclitaxel for 72 h, determined by CCK-8 assay. (**P-Q**) Cell viability (P) and paclitaxel IC_50_ (Q) in H-R-eGFP and H-R-SLC7A11 cells treated with 1.25 mM 3-MA plus paclitaxel for 72 h, determined by CCK-8 assay. Data are presented as mean ± SD. ns, *P* > 0.05; *, *P* < 0.05; **, *P* < 0.01; ***, *P* < 0.001

Our previous bioinformatic and Western blot analyses showed that SLC7A11 mediates autophagy to regulate paclitaxel resistance in OC. Specifically, SLC7A11 overexpression upregulated key autophagy-related targets (STX17, UVRAG, and RAB33B) in both H-R and H cells under paclitaxel treatment [17] (Fig. S3). To further investigate whether autophagy mediates the chemosensitizing effect of SLC7A11, we first examined ultrastructural changes using transmission electron microscopy, the gold standard for detecting autophagy-related structures. After 72 h of paclitaxel treatment, both H-R-SLC7A11 and H-SLC7A11 cells displayed significantly increased numbers of autophagosomes, lysosomes, and autolysosomes compared to their respective eGFP-control cells, in a dose-dependent manner (Fig. 2A). The presence of abundant autolysosomes, rather than solely double-membraned autophagosomes, is morphologically suggestive of ongoing autophagic degradation rather than a blockade. Notably, under high paclitaxel concentrations, apoptotic bodies containing abundant autolysosomes were observed in SLC7A11-overexpressing cells, suggesting a link between enhanced autophagy-associated activity and cell death (Fig. 2A). Consistent with these observations, immunofluorescence and Western blot analyses confirmed the upregulation of autophagy-related markers. LC3B and LAMP1 were significantly elevated in SLC7A11-overexpressing cells (*P* < 0.05) (Fig. 2B-E). Furthermore, the LC3B-II/LC3B-I ratio, a key indicator of autophagy-associated activity, was markedly increased (*P* < 0.05) (Fig. 2D-E). Mechanistically, SLC7A11 overexpression in H-R cells modulated core autophagy regulators. Specifically, it decreased the protein levels of PI3K p110α, mTOR, and p-mTOR, while increasing the expression of Beclin-1 (*P* < 0.01) (Fig. 2F-G). Additionally, it upregulated Rab7, a GTPase critical for autophagosome-lysosome fusion (*P* < 0.01) (Fig. 2F-G). The coordinated upregulation of LAMP1, Rab7, and LC3B-II suggests enhanced autophagy-associated activity, although definitive flux measurements are required to confirm that autophagic degradation is increased.

#### Inhibition of autophagosome formation partially reverses the effect of SLC7A11 overexpression on paclitaxel resistance in OC

To establish a causal link between enhanced autophagy and SLC7A11-mediated chemosensitization, we inhibited autophagosome formation by knocking down LC3B or treating cells with the autophagy inhibitor 3-methyladenine (3-MA). We first confirmed that LC3B expression was significantly lower in paclitaxel-resistant H-R cells than in sensitive H cells (*P* < 0.001) (Fig. 2H), and that SLC7A11 overexpression in H-R cells restored LC3B expression at both mRNA and protein levels (Fig. 2L and B-E). LC3B knockdown in H-R cells (H-R-si-LC3B) markedly increased paclitaxel resistance and elevated the IC₅₀ of paclitaxel compared to control cells (*P* < 0.01) (Fig. 2I-K). Critically, when LC3B was knocked down in both control (H-R-eGFP) and SLC7A11-overexpressing (H-R-SLC7A11) cells, the chemosensitizing effect of SLC7A11 was substantially attenuated. Notably, the IC₅₀ of SLC7A11-overexpressing cells with LC3B knockdown remained lower than that of control cells with LC3B knockdown (*P* < 0.01), indicating a partial reversal (Fig. 2M-O). Consistently, pharmacological inhibition of autophagy using 1.25 mmol/L 3-MA partially reversed the reduction in H-R cell viability conferred by SLC7A11 overexpression under paclitaxel treatment (*P* < 0.05) (Fig. 2P-Q).

### Overexpression of SLC7A11 suppresses the tumorigenicity and paclitaxel resistance of OC cells and is associated with autophagy in vivo

**Fig. 3:**
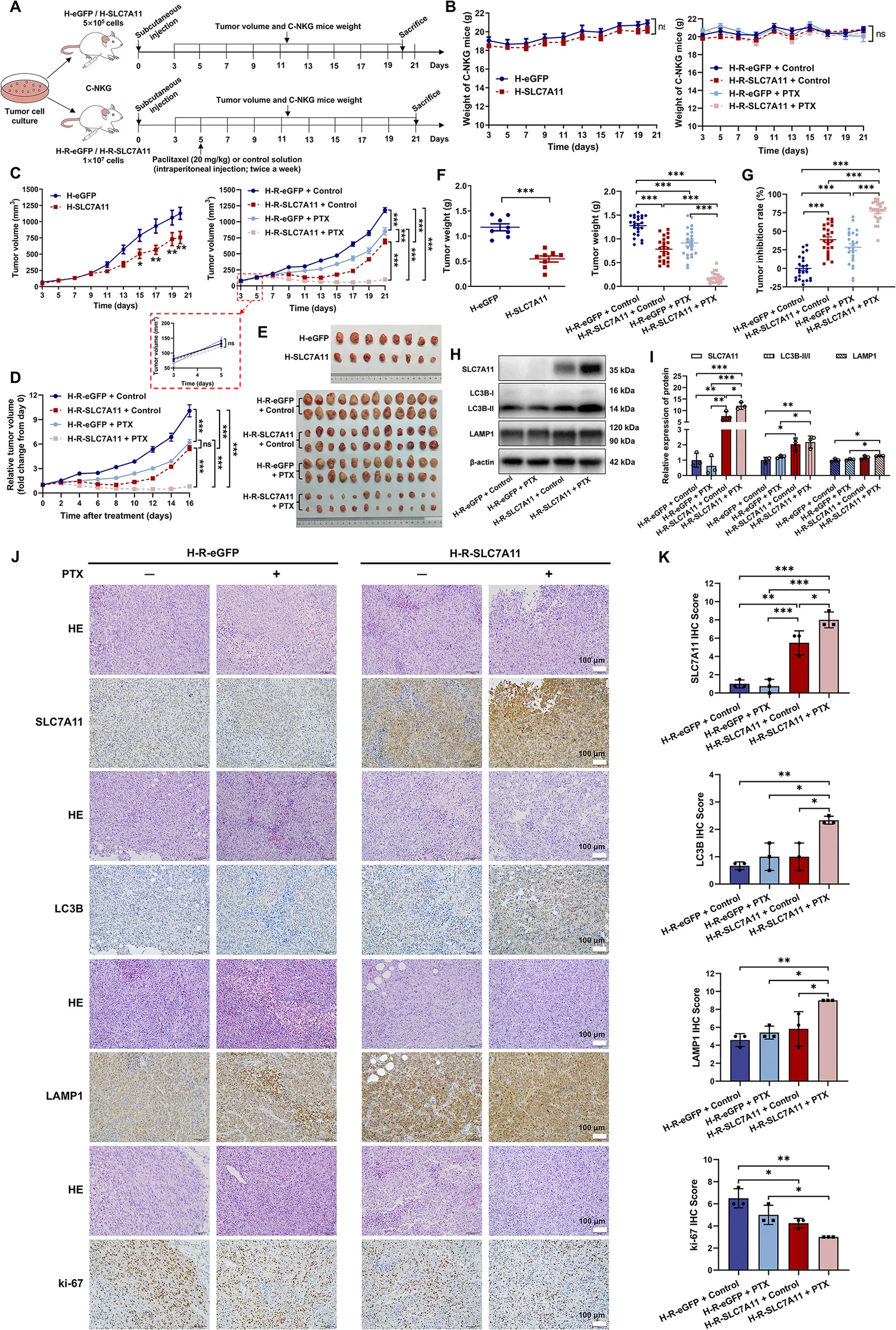
SLC7A11 regulates tumorigenicity, paclitaxel resistance, and autophagy-related protein expression in xenografts of ovarian cancer parental (HeyA8, H) and paclitaxel-resistant (HeyA8-R, H-R) cells. (**A**) Schematic of subcutaneous xenograft models in C-NKG mice: H-eGFP vs. H-SLC7A11 (*n* = 8/group) and H-R-eGFP+Control/PTX vs. H-R-SLC7A11 + Control/PTX (*n* = 24/group). (**B**) Body weight curves. (**C**) Tumor volume curves. Insets show magnified views of tumor volumes at the time of treatment initiation (day 5). (**D**) Tumor growth normalized to individual baseline volume at treatment initiation (day 5). (**E**) Images of xenograft tumors. (**F**) Tumor weight quantification. (**G**) Tumor inhibition rate: H-R-eGFP+Control/PTX vs. H-R-SLC7A11 + Control/PTX. (**H-I**) Western blotting (H) and ImageJ quantification (I) of SLC7A11, LC3B-Ⅱ, and LAMP1 protein levels in H-R-eGFP+Control/PTX vs. H-R-SLC7A11 + Control/PTX xenografts; β-actin as loading control. (**J-K**) HE and IHC staining (J) and IHC scoring quantification (K) of SLC7A11, LC3B-Ⅱ, LAMP1, and Ki-67 protein levels in H-R-eGFP+Control/PTX vs. H-R-SLC7A11 + Control/PTX xenografts. PTX, paclitaxel. HE, hematoxylin-eosin staining. IHC, immunohistochemical staining. Data are presented as mean ± SEM or mean ± SD. *, *P* < 0.05; **, *P* < 0.01; ***, *P* < 0.001

To validate the translational relevance of our findings, we established subcutaneous xenograft models using C-NKG mice implanted with SLC7A11-overexpressing or control OC cells (Fig. 3A). Compared to their respective control groups (H-eGFP or H-R-eGFP), mice bearing H-SLC7A11 or H-R-SLC7A11 tumors exhibited markedly slower tumor growth, reduced final tumor volumes, and lower tumor weights (*P* < 0.05) (Fig. 3C-F). Baseline tumor volumes at treatment initiation (day 5) were comparable among all groups (*P* > 0.05; Fig. 3C insets), confirming successful randomization. In paclitaxel-resistant models, the combination of SLC7A11 overexpression and paclitaxel treatment (H-R-SLC7A11 + PTX) produced the most potent antitumor effect, with significantly smaller tumor volumes and lower tumor weights compared to all other groups (*P* < 0.001) (Fig. 3C-F). The tumor inhibition rate in the H-R-SLC7A11 + PTX group was higher than that in the H-R-eGFP + PTX and H-R-SLC7A11 + Control groups (*P* < 0.001) (Fig. 3G). SLC7A11 overexpression alone (H-R-SLC7A11 + Control) also conferred a significant baseline inhibitory effect, with a tumor inhibition rate 38.68% points higher than that of the H-R-eGFP+Control group (*P* < 0.001) (Fig. 3G). Analysis of tumor growth normalized to individual baseline volume at treatment initiation confirmed the intrinsic tumor-suppressive effect of SLC7A11, as the H-R-SLC7A11 + Control group showed significantly lower relative tumor volume compared to H-R-eGFP+Control (*P* < 0.001). The H-R-SLC7A11 + PTX group exhibited a further significant reduction in relative tumor volume compared to H-R-SLC7A11 + Control (*P* < 0.001), supporting an additional paclitaxel-sensitizing effect (Fig. 3D). None of the treatments significantly affected mouse body weight, indicating good tolerability (Fig. 3B).

To assess whether these antitumor effects were associated with autophagy in vivo, we analyzed key protein markers in xenograft tissues. Western blot and IHC analyses confirmed that SLC7A11, the LC3B-II/I ratio, and LAMP1 were significantly elevated in H-R-SLC7A11 + Control and H-R-SLC7A11 + PTX tumors compared to control tumors (*P* < 0.05) (Fig. 3H-K). These findings are consistent with our cellular-level data (Fig. 2B-E). Furthermore, these tumors showed a marked reduction in the proliferation marker Ki-67 (*P* < 0.05) (Fig. 3J-K), correlating with the observed decreases in tumor volume and weight (Fig. 3C-F).

### Low SLC7A11 expression correlates with chemoresistance and poor prognosis in OC patients

**Fig. 4:**
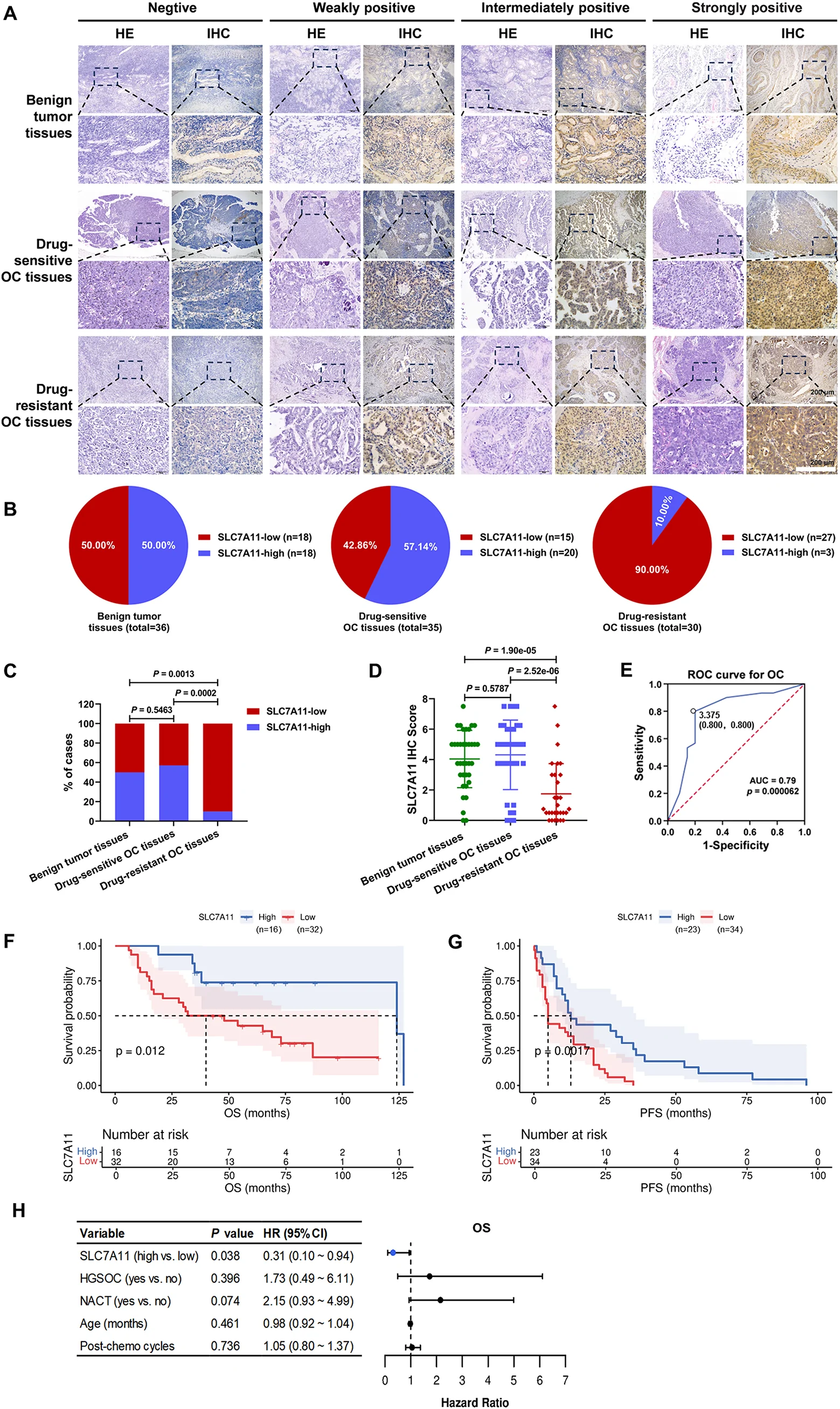
SLC7A11 correlates with chemoresistance and independent prognosis in ovarian cancer (OC). (**A**) Representative hematoxylin-eosin (HE) and immunohistochemical (IHC) staining images of SLC7A11 expression (negative, weakly/intermediately/strongly positive) in benign tumor tissues (uterine leiomyoma, *n* = 36), chemosensitive OC tissues (*n* = 35), and chemoresistant OC tissues (*n* = 30). Scale bar: 200 μm. (**B**) Distribution of SLC7A11 expression across three cohorts, stratified by a predefined IHC score cutoff of 5 (low: <5; high: ≥5). (**C-D**) Quantitative comparisons of SLC7A11 expression via chi-square test (C) and one-way ANOVA (D). (**E**) Receiver operating characteristic (ROC) curve analysis of SLC7A11 IHC score for OC chemotherapy sensitivity prediction. (**F**) Kaplan-Meier curve of OS in the 48-patient cohort with available OS follow-up data, stratified by SLC7A11 expression, with matched number-at-risk table. *P* value by log-rank test. (**G**) Kaplan-Meier curve of PFS in the 57-patient cohort with documented recurrence records, stratified by SLC7A11 expression, with matched number-at-risk table. *P* value by log-rank test. (**H**) Forest plot of multivariate Cox regression for OS in the 48-patient cohort, adjusted for SLC7A11 expression, HGSOC histology, NACT status, age, and postoperative chemotherapy cycles. PFS, progression-free survival. OS, overall survival. HGSOC, High-grade serous ovarian cancer. NACT, Neoadjuvant chemotherapy. HR, Hazard ratio. CI, Confidence interval

To assess the clinical relevance of our findings, we analyzed SLC7A11 expression in benign uterine leiomyomas (*n* = 36), chemotherapy-sensitive (*n* = 35), and chemotherapy-resistant OC tissues (*n* = 30). Uterine leiomyoma does not represent an ideal biological control for OC. Histological assessment by HE staining confirmed the expected morphological characteristics of each tissue type (Fig. 4A). IHC analysis revealed membranous and cytoplasmic SLC7A11 expression (Fig. 4A). IHC scores demonstrated a significant association between low SLC7A11 expression and chemoresistance. Specifically, 90% (27/30) of chemoresistant tumors exhibited low SLC7A11 expression, compared to only 10% (3/30) with high expression (Fig. 4B). SLC7A11 levels were significantly lower in chemoresistant tissues than in chemosensitive tissues (*P* < 0.001) (Fig. 4C-D). SLC7A11 expression in benign uterine leiomyoma tissues was also examined for reference. However, given the distinct tissue origin, this comparison should be interpreted with caution (Fig. 4C-D). ROC curve analysis demonstrated that SLC7A11 expression had significant predictive value for chemotherapy response in OC (AUC = 0.79, *P* < 0.001) (Fig. 4E). The optimal cutoff value of the SLC7A11 IHC score was 3.375, yielding a sensitivity of 0.800 and specificity of 0.800 (Fig. 4E).

Survival analysis revealed a significant prognostic value for SLC7A11 expression. Using the pre-specified IHC cutoff of 5, high SLC7A11 expression (*n* = 16) was associated with significantly longer OS compared to low expression (*n* = 32) in the 48-patient OS cohort (*P* = 0.012) (Fig. 4F). Multivariate Cox regression analysis adjusting for histologic subtype, neoadjuvant chemotherapy, age, and total postoperative chemotherapy courses confirmed that high SLC7A11 expression was an independent predictor of favorable OS (HR = 0.31, 95% CI: 0.10–0.94, *P* = 0.038) (Fig. 4H). Similarly, using the same cutoff, high SLC7A11 expression (*n* = 23) was associated with significantly longer PFS than low expression (*n* = 34) in the 57-patient PFS cohort (*P* = 0.0017) (Fig. 4G). As all 57 patients experienced disease progression, multivariate Cox regression for PFS was not feasible. Histologic subtype, age, and total postoperative chemotherapy courses were not independently associated with OS (all *P* > 0.05) (Fig. 4H). FIGO stage and residual disease status were not included in the multivariate model due to incomplete data for the full cohort. Sensitivity analysis using the ROC-derived cutoff of 3.375 yielded consistent results (Fig. S4).

### SLC7A11 negatively regulates KLF2 and may jointly modulate autophagy and paclitaxel resistance in OC

#### Proteomic profiling reveals molecular networks underlying SLC7A11-mediated chemosensitization

**Fig. 5:**
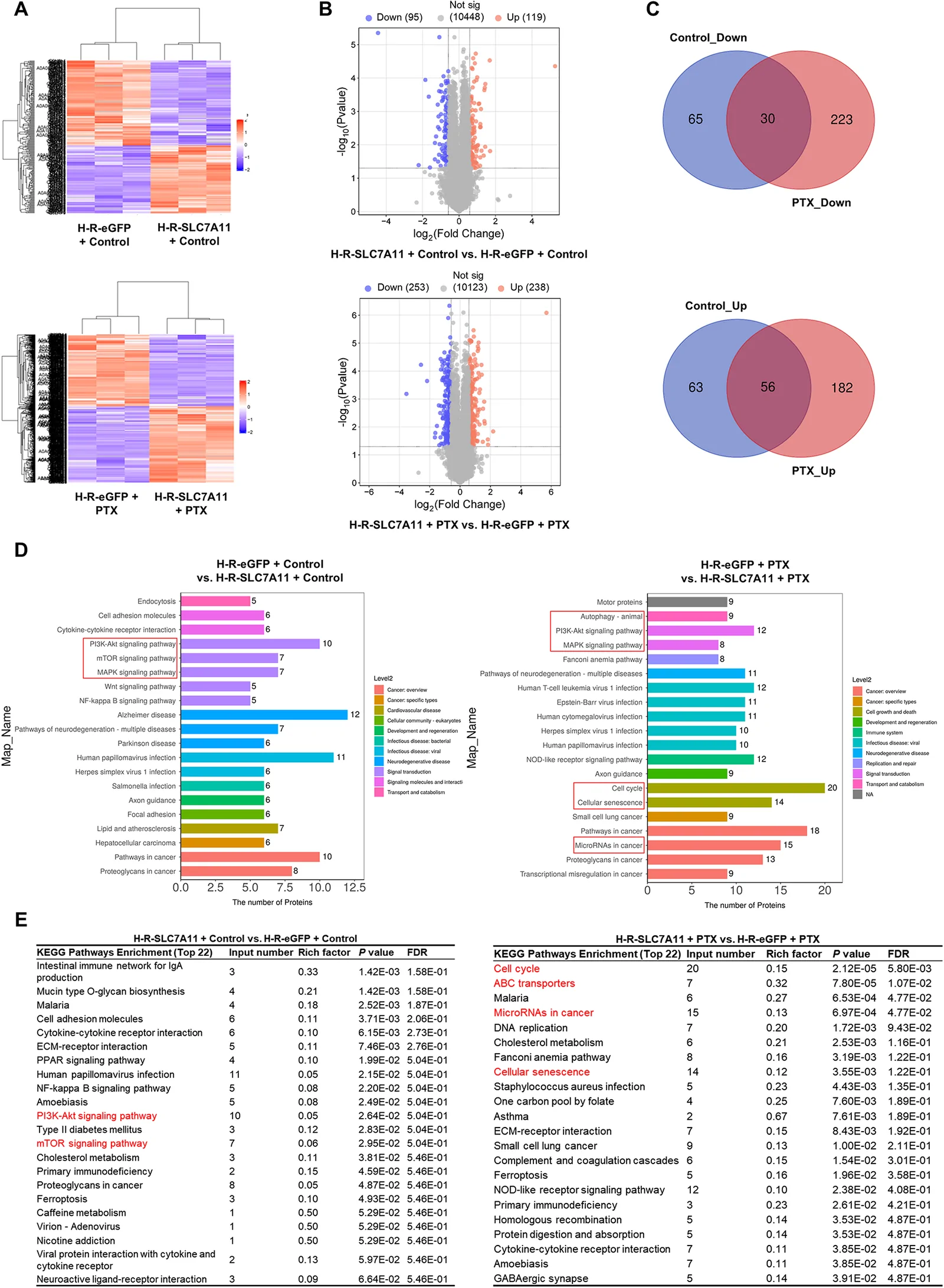
The effect of SLC7A11 overexpression on protein expression profile in paclitaxel-resistant ovarian cancer cells (HeyA8-R, H-R) under paclitaxel (PTX) treatment. (**A**) Hierarchical clustering heatmaps of differentially expressed proteins in H-R-SLC7A11 vs. H-R-eGFP cells, under Control (top) or PTX (31.25 nmol/L, 72 h; bottom) conditions. (**B**) Volcano plots of differential proteins: H-R-SLC7A11 + Control vs. H-R-eGFP+Control (top) and H-R-SLC7A11 + PTX vs. H-R-eGFP + PTX (bottom). (**C**) Venn diagrams showing overlapping/unique down-regulated (top) and up-regulated (bottom) proteins between the two comparison groups. (**D**) Top 20 KEGG pathway categories of differentially expressed proteins ranked by protein count, colored by KEGG secondary functional classification. Pathway enrichment statistics (raw *P* value and Benjamini-Hochberg FDR) are provided in Supplementary Table 4. (**E**) Top 22 enriched KEGG pathways for both comparisons, with input number, Rich factor, raw *P*, and Benjamini-Hochberg FDR values shown

To comprehensively investigate the molecular mechanisms by which SLC7A11 inhibits paclitaxel resistance, we performed quantitative proteomic analysis of control (H-R-eGFP) and SLC7A11-overexpressing (H-R-SLC7A11) cells, with and without paclitaxel treatment (31.25 nmol/L, 72 h). SLC7A11 overexpression substantially remodeled the cellular proteome. In untreated conditions, 214 proteins were differentially expressed (95 downregulated, 119 upregulated) (Fig. 5B). Paclitaxel treatment amplified this effect, with 491 proteins showing altered expression (253 downregulated, 238 upregulated) in SLC7A11-overexpressing cells (Fig. 5B). Notably, 86 proteins displayed consistent expression changes across both conditions (30 downregulated, 56 upregulated), representing core molecular targets of SLC7A11 (Fig. 5C).

KEGG pathway classification was first performed to profile the functional distribution of differentially expressed proteins (Fig. 5D). In untreated cells, the PI3K-Akt, mTOR, and MAPK signaling pathways were prominently represented. Upon paclitaxel treatment, autophagy-animal, PI3K-Akt, MAPK signaling, cell cycle, cellular senescence, and microRNAs in cancer ranked among the top functional categories. Formal KEGG pathway enrichment analysis with Benjamini-Hochberg FDR correction was then performed (Fig. 5E, S5). In control cells, PI3K-Akt and mTOR signaling showed only nominal enrichment (raw *P* < 0.05) and did not survive FDR adjustment. In paclitaxel-treated cells, cell cycle, ABC transporters, and microRNAs in cancer reached FDR < 0.05 in the main differentially expressed protein set; mitophagy-animal, ferroptosis, and proteoglycans in cancer reached significance in the FDR-stratified proteomic analysis (Fig. S5). Autophagy – animal and cellular senescence showed only nominal enrichment trends. At the protein level, eight essential autophagy components (MRAS, GABARAP, GABARAPL1, GABARAPL2, SQSTM1, NBR1, TAX1BP1, and ZFYVE1) were upregulated and CAMKK2 was downregulated in SLC7A11-overexpressing cells, further supporting SLC7A11-mediated regulation of the autophagy machinery. Complete FDR-corrected datasets are provided in the Supplementary Materials (Tables S4-5).

#### SLC7A11 suppresses drug resistance and improves OC patient prognosis by downregulating the autophagy regulator KLF2

**Fig. 6:**
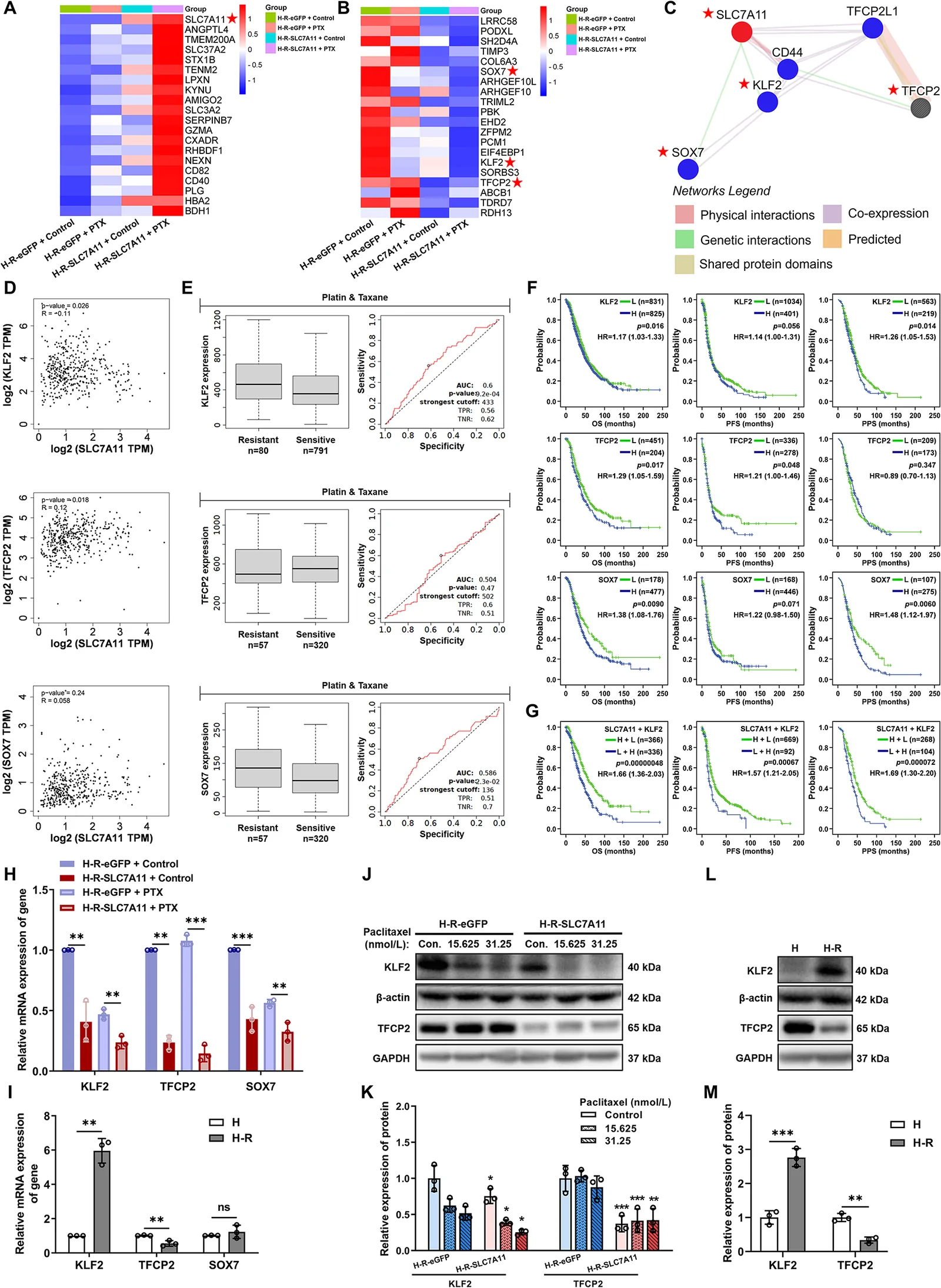
Screening and validation of target genes for potential regulation of SLC7A11. (**A-B**) Heatmaps of top 20 up-regulated (A) or down-regulated (B) proteins common to H-R-SLC7A11 vs. H-R-eGFP comparisons with or without paclitaxel treatment (31.25 nmol/L, 72 h). (**C**) GeneMANIA predicted functional association network of SLC7A11 with KLF2, TFCP2, and SOX7. Lines denote predicted functional associations. (**D**) Pearson correlation of SLC7A11 with KLF2, TFCP2, and SOX7 mRNA expression in the TCGA ovarian serous cystadenocarcinoma cohort, analyzed using GEPIA. R, correlation coefficient. (**E**) ROC curve analysis of KLF2, TFCP2, and SOX7 expression for predicting platinum/taxane resistance in ovarian cancer (ROC Plotter). AUC, area under the curve. (**F-G**) Kaplan-Meier survival analysis of KLF2, TFCP2, SOX7, and combined SLC7A11 + KLF2 in ovarian cancer patients (Kaplan-Meier plotter). H, high expression; L, low expression; OS, overall survival; PFS, progression-free survival; PPS, survival after progression; HR, hazard ratio. (**H**) RT-qPCR analysis of KLF2, TFCP2, and SOX7 mRNA in H-R-eGFP and H-R-SLC7A11 cells treated with 0 or 31.25 nmol/L paclitaxel for 72 h. (**I**) RT-qPCR analysis of KLF2, TFCP2, and SOX7 mRNA in ovarian cancer parental (HeyA8, H) and paclitaxel-resistant (HeyA8-R, H-R) cells. (**J-K**) Western blotting (J) and ImageJ quantification (K) of KLF2 and TFCP2 protein in H-R-eGFP vs. H-R-SLC7A11 cells treated with paclitaxel for 72 h. (**L-M**) Western blotting (L) and ImageJ quantification (M) of KLF2 and TFCP2 protein in H vs. H-R cells. Data are presented as mean ± SD. ns, *P* > 0.05; *, *P* < 0.05; **, *P* < 0.01; ***, *P* < 0.001

Based on the proteomic profiles, we focused on the top 20 differentially expressed proteins showing consistent up- or down-regulation in both untreated and paclitaxel-treated H-R-eGFP and H-R-SLC7A11 cells (Fig. 6A-B, Table S6). From these 40 candidate proteins, three transcription factors—SOX7, KLF2, and TFCP2—were significantly downregulated upon SLC7A11 overexpression (*P* < 0.01) (Fig. 6A-B). Notably, KLF2 overexpression has been reported to suppress autophagy-related proteins, whereas its knockdown elevates Beclin-1 expression, consistent with the autophagy-modulating role of SLC7A11 observed in our study [27]. GeneMANIA analysis suggested potential functional links between SLC7A11 and KLF2/SOX7, and between SLC7A11 and TFCP2 through CD44 and TFCP2L1 (Fig. 6C). In the TCGA ovarian serous cystadenocarcinoma cohort, KLF2 expression exhibited a significant negative correlation with SLC7A11 (*R* = − 0.11, *P* < 0.05) (Fig. 6D). High KLF2 expression predicted resistance to platinum and taxane chemotherapy in OC (AUC = 0.6, *P* < 0.001) (Fig. 6E). Prognostic analysis showed that high expression of all three transcription factors correlated with poorer OS, PFS, and PPS in OC patients (Fig. 6F). The combination of low SLC7A11 and high KLF2 expression demonstrated enhanced prognostic predictive power (Fig. 6G), consistent with our previous observation that low SLC7A11 expression is an independent risk factor for OC prognosis [17]. Experimental validation at both mRNA and protein levels showed that SLC7A11 overexpression downregulates KLF2 and TFCP2 under paclitaxel treatment (Fig. 6H, J, K). Furthermore, KLF2 was specifically upregulated in paclitaxel-resistant H-R cells compared to parental H cells (Fig. 6I, L, M), suggesting a potential role in chemoresistance. These results suggest that KLF2 may serve as a downstream mediator of SLC7A11 in regulating autophagy and paclitaxel resistance in OC, with combined SLC7A11 and KLF2 expression offering improved prognostic value.

#### SLC7A11 negatively regulates KLF2 and may jointly modulate autophagy to suppress paclitaxel resistance in OC

**Fig. 7:**
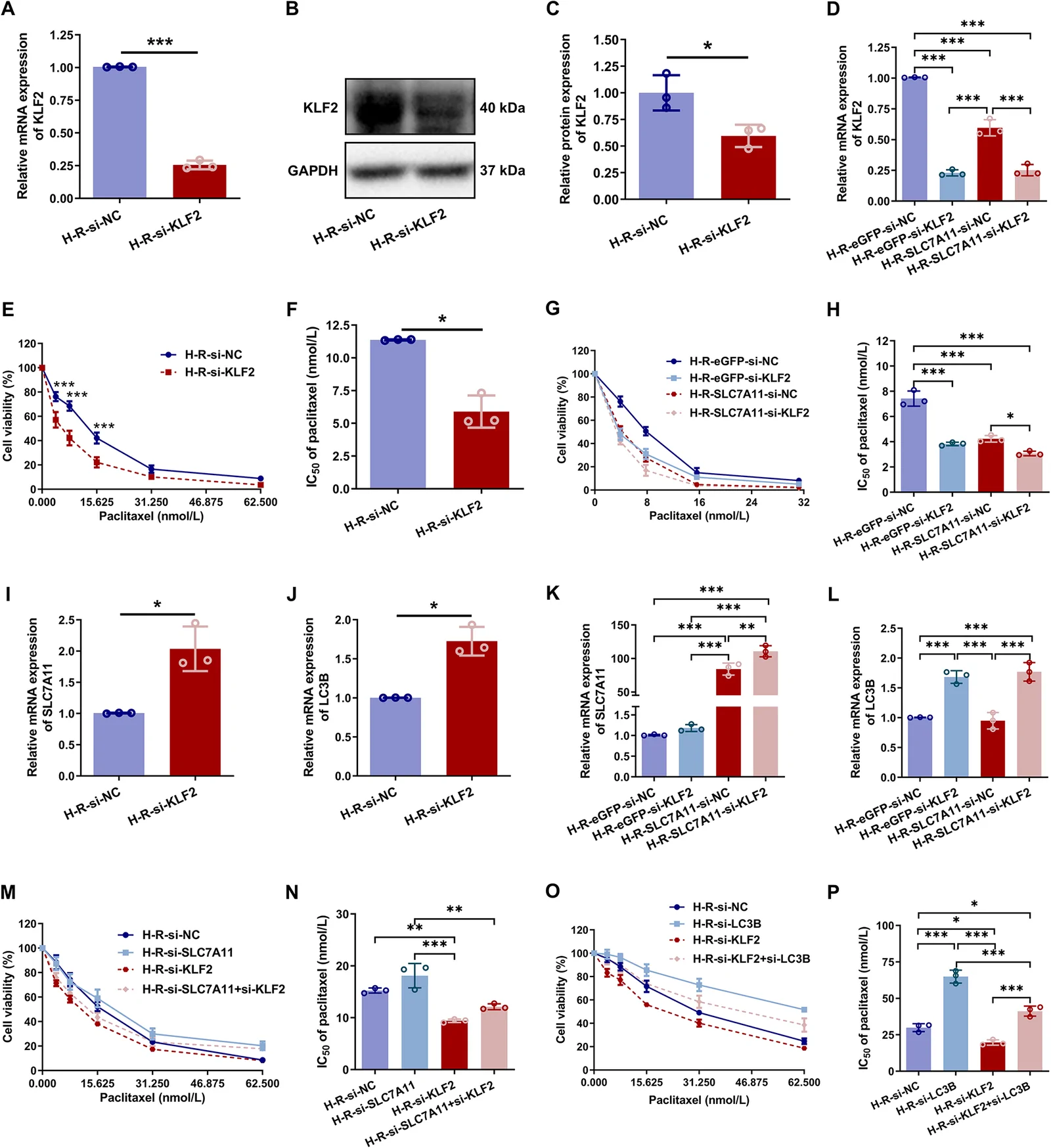
Effect of KLF2 knockdown on paclitaxel resistance, SLC7A11, and LC3B expression levels in paclitaxel-resistant ovarian cancer cells (HeyA8-R, H-R). (**A-C**) KLF2 expression in H-R-si-KLF2 vs. H-R-si-NC cells: (A) mRNA (RT-qPCR); (B) protein (Western blotting); (C) protein quantification (ImageJ). (**D**) KLF2 mRNA expression in H-R-eGFP-si-KLF2 and H-R-SLC7A11-si-KLF2 vs. respective si-NC controls. (**E-H**) Cell viability (E, G) and paclitaxel IC₅₀ (F, H) in H-R-si-KLF2 vs. H-R-si-NC (E-F) and H-R-eGFP-si-KLF2/H-R-SLC7A11-si-KLF2 vs. respective si-NC controls (G-H) treated with paclitaxel for 72 h, determined by CCK-8 assay. (**I-L**) RT-qPCR analysis of SLC7A11 (I, K) and LC3B (J, L) mRNA expression in H-R-si-KLF2 vs. H-R-si-NC (I-J) and H-R-eGFP-si-KLF2/H-R-SLC7A11-si-KLF2 vs. respective si-NC controls (K-L) cells. (**M-N**) Cell viability (M) and paclitaxel IC₅₀ (N) in H-R-si-NC, H-R-si-SLC7A11, H-R-si-KLF2, and H-R-si-SLC7A11 + si-KLF2 cells treated with paclitaxel for 72 h, determined by CCK-8 assay. (**O-P**) Cell viability (O) and paclitaxel IC₅₀ (P) in H-R-si-NC, H-R-si-LC3B, H-R-si-KLF2, and H-R-si-KLF2 + si-LC3B cells treated with paclitaxel for 72 h, determined by CCK-8 assay. Data are presented as mean ± SD. *, *P* < 0.05; **, *P* < 0.01; ***, *P* < 0.001

To further clarify the role of KLF2 in the autophagy pathway associated with SLC7A11, we conducted a series of functional validation experiments. KLF2 knockdown in H-R cells (Fig. 7A–C) significantly sensitized cells to paclitaxel, as evidenced by reduced cell viability (*P* < 0.001) and decreased IC₅₀ (*P* < 0.05) (Fig. 7E–F). Similar chemosensitization was observed when KLF2 was knocked down in both control (H-R-eGFP) and SLC7A11‑overexpressing (H-R‑SLC7A11) cells (*P* < 0.05) (Fig. 7D and G–H). Mechanistically, KLF2 knockdown upregulated both SLC7A11 and LC3B mRNA expression (*P* < 0.05) (Fig. 7I–L). To examine the regulatory relationship between KLF2 and SLC7A11, we performed dual‑knockdown experiments. Simultaneous knockdown of KLF2 and SLC7A11 in H‑R cells resulted in cell viability and IC₅₀ values intermediate between those of the individual knockdowns (*P* < 0.01) (Fig. 7M–N). Similarly, knockdown of KLF2 and LC3B produced an intermediate phenotype (*P* < 0.001) (Fig. 7O-P). These findings suggest that SLC7A11 negatively regulates KLF2, and the two factors may jointly influence paclitaxel resistance in OC cells through autophagy modulation.

## Discussion

The development of drug resistance remains a major cause of treatment failure in OC. Although chemotherapy is the mainstay of treatment for patients with OC before and after cytoreductive surgery [4], initial responsiveness often diminishes due to acquired drug resistance, leading to disease progression [4]. Consequently, elucidating the molecular mechanisms governing chemoresistance is crucial. Notably, SLC7A11 has emerged as a promising therapeutic target to counteract chemoresistance across various cancers [9, 12, 16], and modulating autophagy represents another viable strategy [20]. SLC7A11 encodes a light chain subunit of the Xc⁻-system, functioning as a cystine/glutamate antiporter [43]. Our previous study provided preliminary evidence for SLC7A11’s role in paclitaxel resistance in vitro, with bioinformatics-based correlations to prognosis and autophagy, and Western blot validation of seven autophagy-related proteins [17, 18]. The present study addresses these gaps by incorporating additional cell lines, animal models, clinical samples, transmission electron microscopy, and proteomics to identify KLF2, thereby strengthening the conclusions and revealing a novel SLC7A11-KLF2 axis with potential clinical relevance.

Autophagy proceeds through four main stages: initiation, autophagosome formation, autophagosome-lysosome fusion, and degradation [44]. Growing evidence indicates that autophagy dysregulation is closely associated with tumor drug resistance [45, 46]. For instance, autophagy induction can inhibit paclitaxel resistance in nasopharyngeal carcinoma cells [21], cisplatin resistance in bladder cancer [47] and non-small cell lung cancer [48], gefitinib resistance in non-small cell lung cancer cells [49] and sorafenib resistance in liver cancer cells [50]. In contrast, autophagy inhibition can enhance paclitaxel resistance in lung cancer cells [51]. In this study, SLC7A11 overexpression was associated with significantly increased autophagosomes, autolysosomes, and apoptotic bodies in OC cells under paclitaxel treatment, accompanied by upregulation of autophagy-related proteins (LC3B-II/LC3B-I, LAMP1, Rab7, TFEB, and Beclin-1) and downregulation of autophagy inhibitory proteins (PI3K p110α, mTOR, and p-mTOR). Moreover, differentially expressed autophagy-related proteins were enriched in the Autophagy-animal pathway and three key upstream signaling pathways (PI3K-Akt, mTOR, and MAPK). The Akt-mTOR pathway negatively regulates autophagy. Inhibition of Akt/mTOR activity can promote autophagosome formation, thereby initiating autophagy and enhancing the anticancer effects of chemotherapeutic drugs [52, 53]. Increased LAMP1, Rab7, and LC3-II expression can enhance autophagy-associated activity and promote cell death in melanoma and OC cells [54, 55]. LC3 knockdown or 3-methyladenine (3-MA) treatment blocks autophagosome formation and reverses the enhancement of sorafenib sensitivity in hepatocellular carcinoma induced by CASK knockout [56–58]. We found that the inhibitory effect of SLC7A11 overexpression on paclitaxel resistance in OC was partially reversed by blocking autophagosome formation.

Previous studies have shown that paclitaxel exerts its anticancer effects by disrupting mitosis [38, 39] and inducing DNA double-strand breaks [38–42, 59], while increased drug efflux contributes to paclitaxel resistance [38]. Consistently, we found that SLC7A11 overexpression increased intracellular paclitaxel concentration, was accompanied by ABCB1 downregulation, and enhanced DNA damage, suggesting that autophagy may be associated with drug accumulation and apoptotic signaling. In vivo studies showed that SLC7A11 overexpression suppresses OC tumorigenicity and paclitaxel resistance, and this was accompanied by upregulated autophagy-related markers (LC3B, LC3B-II/I, and LAMP1). Collectively, these findings indicate that SLC7A11 enhances paclitaxel sensitivity in OC, and this effect is associated with the coordination of autophagy.

To further explore the molecular mechanism linking SLC7A11 and autophagy in drug resistance, we integrated proteomics, bioinformatics, and expression validation to identify KLF2 as a candidate downstream target of SLC7A11. We found that SLC7A11 overexpression downregulated KLF2 expression, and was functionally associated with KLF2. Clinically, high KLF2 expression predicted resistance to platinum-taxane chemotherapy in OC. The combination of high KLF2 and low SLC7A11 exhibited superior prognostic predictive value, highlighting the clinical relevance of this regulatory pair. Previous studies have characterized KLF2 as an oncogenic factor that reduces tumor cell sensitivity to multiple chemotherapeutic agents, serving as an independent prognostic risk factor in adrenocortical carcinoma, colon cancer, uveal melanoma, lung squamous cell carcinoma, cervical squamous cell carcinoma, and adenocarcinoma [28–31]. Furthermore, KLF2 has been shown to inhibit autophagy by decreasing autophagic vesicles and downregulating Beclin-1, ATG3, ATG5, and LC3B-II/I levels, while KLF2 knockdown promotes Beclin-1 expression [27], consistent with the autophagy-regulating role of SLC7A11 in our study. In OC cells, we observed that KLF2 knockdown inhibited paclitaxel resistance and upregulated SLC7A11 and LC3B expression. Notably, KLF2 negatively regulates SLC7A11 in prostate cancer [56], which is consistent with our finding that SLC7A11 overexpression downregulates KLF2. The inhibitory effect of KLF2 knockdown on paclitaxel resistance was attenuated by SLC7A11 or LC3B knockdown, indicating that SLC7A11 and KLF2 may jointly modulate autophagy to influence paclitaxel resistance in OC. Whether this constitutes a bona fide reciprocal negative feedback loop remains to be determined. This regulatory axis provides a novel framework for understanding the potential role of SLC7A11 in autophagy and drug sensitivity.

SLC7A11 is a well-established ferroptosis inhibitor that promotes GSH synthesis [6, 43]. Our finding that SLC7A11 overexpression nevertheless sensitizes OC cells to paclitaxel suggests that its autophagy-promoting function may dominate over its anti-ferroptotic role in this context. Notably, crosstalk between autophagy and ferroptosis has been reported, with autophagy capable of promoting ferroptosis through ferritinophagy [60, 61]. We propose that these opposing effects coexist, and that the pro-death autophagy function dominates under paclitaxel treatment. Direct experimental investigation of this balance, including GSH/GSSG, ROS, and lipid peroxidation measurements, will be needed to clarify the underlying mechanism.

Consistent with our previous analyses [17, 18], clinical tissue analysis in this study demonstrated that SLC7A11 is significantly downregulated in chemoresistant OC tissues and correlates with poor OS and PFS of OC patients. Furthermore, our findings indicate that SLC7A11 expression showed significant predictive value for OC chemotherapy response. The clinical relevance of SLC7A11 is further supported by cross-tumor evidence. For example, SLC7A11 is downregulated in doxorubicin-resistant breast cancer cells, and its overexpression increases doxorubicin sensitivity [12]. Inhibition of MYH9 expression has been shown to promote SLC7A11 expression and induce disulfidptosis, thereby improving the sensitivity of sorafenib-resistant liver cancer cells to sorafenib [16]. Collectively, these data suggest that SLC7A11 may serve as both a prognostic biomarker and a potential therapeutic target for overcoming OC chemoresistance.

Several limitations should be acknowledged. Definitive autophagic flux was not measured using lysosomal inhibitor-based assays or tandem fluorescent reporters; our conclusions of enhanced autophagy-associated activity are based on TEM and autophagy-related protein markers. LC3B knockdown and 3-MA only partially reversed SLC7A11-mediated paclitaxel sensitization, indicating that autophagy is an important but not the exclusive mechanism. The SLC7A11-KLF2 association was identified through proteomic profiling and bioinformatic prediction, and full validation as a feedback loop requires further experiments. The subcutaneous xenograft model does not fully recapitulate the peritoneal microenvironment. Furthermore, FIGO stage and residual disease were not included in the multivariate model due to incomplete data, and the independent prognostic value of SLC7A11 should be interpreted accordingly. The mechanism was characterized primarily in HeyA8/HeyA8-R cells, extending our prior work in this model [17]. Although the core phenotype was confirmed in SKOV3-R cells, validation in additional HGSOC-representative models is warranted. Finally, this study focused on autophagy and did not assess the canonical anti-ferroptotic function of SLC7A11; the interplay between autophagy and ferroptosis in paclitaxel resistance warrants future investigation.

## Conclusions

In summary, this study further establishes SLC7A11 as a pivotal suppressor of paclitaxel resistance in OC. SLC7A11 enhances chemosensitivity, which is associated with the coordination of autophagy, a process critical for its function in vitro and in vivo. Mechanistically, we identified a functional interplay between SLC7A11 and KLF2, in which SLC7A11 negatively regulates KLF2 and the two factors may jointly modulate autophagy to influence paclitaxel response. Clinically, low SLC7A11 and high KLF2 expression are associated with chemoresistance and poor prognosis. Collectively, our findings suggest that the SLC7A11/KLF2-autophagy axis may contribute to paclitaxel resistance, supporting SLC7A11 as a potential prognostic biomarker and therapeutic target for overcoming chemoresistance in OC.

## Supplementary Information

Below is the link to the electronic supplementary material.

Supplementary Material 1

Supplementary Material 2

Supplementary Material 3

Supplementary Material 4

Supplementary Material 5

Supplementary Material 6

Supplementary Material 7

## Data Availability

The mass spectrometry proteomics data have been deposited to the ProteomeXchange Consortium (https://proteomecentral.proteomexchange.org) via the iProX partner repository [62, 63] with the dataset identifier PXD080398. Other experimental data are available from the corresponding author upon reasonable request. Publicly available datasets analyzed in this study can be found in the GeneMANIA online tool (http://www.genemania.org/), TCGA database (https://portal.gdc.cancer.gov/), GEPIA (http://gepia.cancer-pku.cn/), Kaplan-Meier Plotter (https://kmplot.com/analysis/index.php?p=service&cancer=ovar), and ROC Plotter (https://rocplot.org/ovarian/index).

## Abbreviations

OC: Ovarian cancer
SLC7A11: Solute carrier family 7 member 11
KLF2: Krüppel-like factor 2
3-MA: 3-methyladenine
ABCB1: ATP-binding cassette subfamily B member 1
AUC: Area under the curve
CCK-8: Cell Counting Kit-8
CI: Confidence interval
DIA: Data independent acquisition
DNA: Deoxyribonucleic acid
FBS: Fetal bovine serum
FDR: False discovery rate
GEPIA: Gene Expression Profiling Interactive Analysis
GSH: Glutathione
HE: Hematoxylin-eosin
HGSOC: High-grade serous ovarian cancer
HPLC: High performance liquid chromatography
HR: Hazard ratio
IC₅₀: Half maximal inhibitory concentration
IHC: Immunohistochemical staining
LAMP1: Lysosomal-associated membrane protein 1
LC3B: Microtubule-associated protein 1 light chain 3 beta
MAPK: Mitogen-activated protein kinase
mTOR: Mammalian target of rapamycin
NACT: Neoadjuvant chemotherapy
NC: Negative control
OS: Overall survival
PBS: Phosphate-buffered saline
PFS: Progression-free survival
PI3K: Phosphatidylinositol 3-kinase
PPS: Post-progression survival
PTX: Paclitaxel
RI: Resistance index
ROC: Receiver operating characteristic
RT-qPCR: Real-time quantitative polymerase chain reaction
SD: Standard deviation
SEM: Standard error of the mean
siRNA: Small interfering RNA
TCGA: The Cancer Genome Atlas
TIR: Tumor inhibition rate
