# Supplementary material for: SLC7A11 suppresses paclitaxel resistance and improves survival via the KLF2 regulatory axis associated with autophagy in ovarian cancer
Source: Biol Direct. 2026 Jul 22;21:209. doi: 10.1186/s13062-026-00909-x (PMC13679285; doi:10.1186/s13062-026-00909-x)

Figure 1B： SLC7A11; β-actin

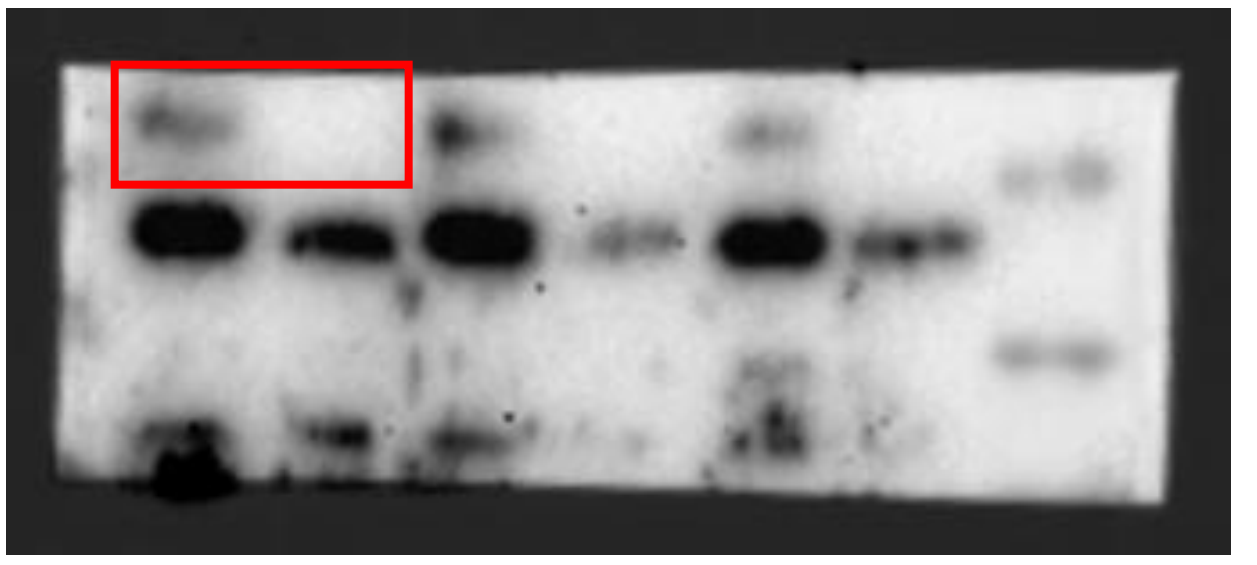

SLC7A11

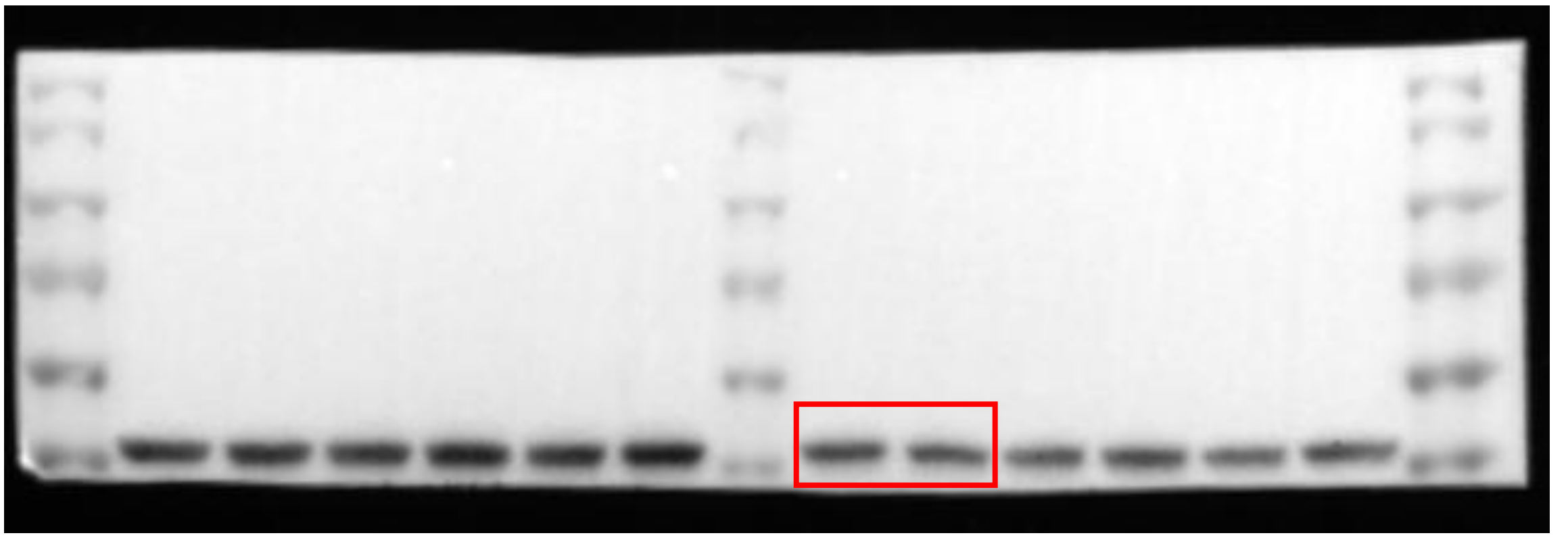

β-actin

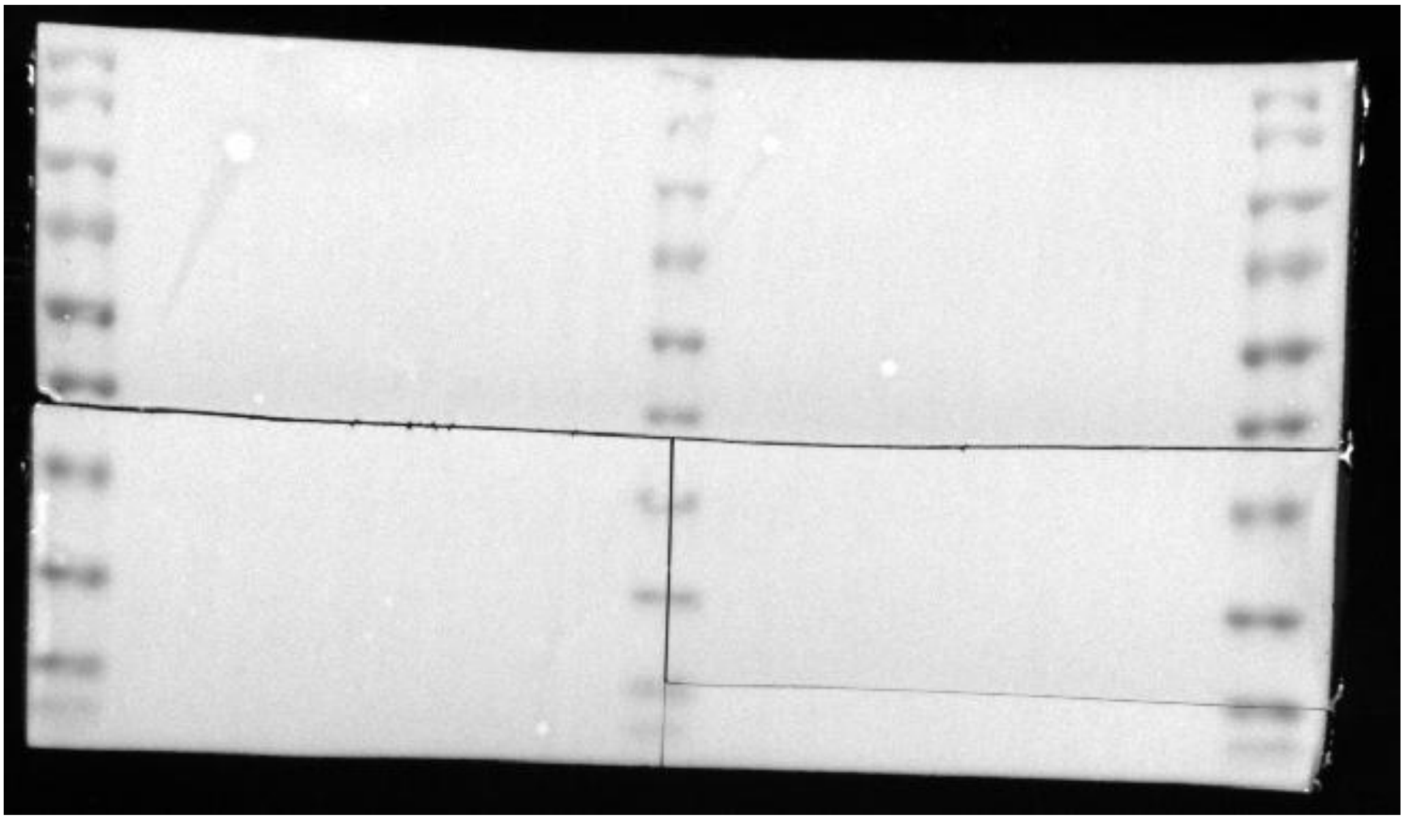

Figure 1F： SLC7A11; β-Tubulin

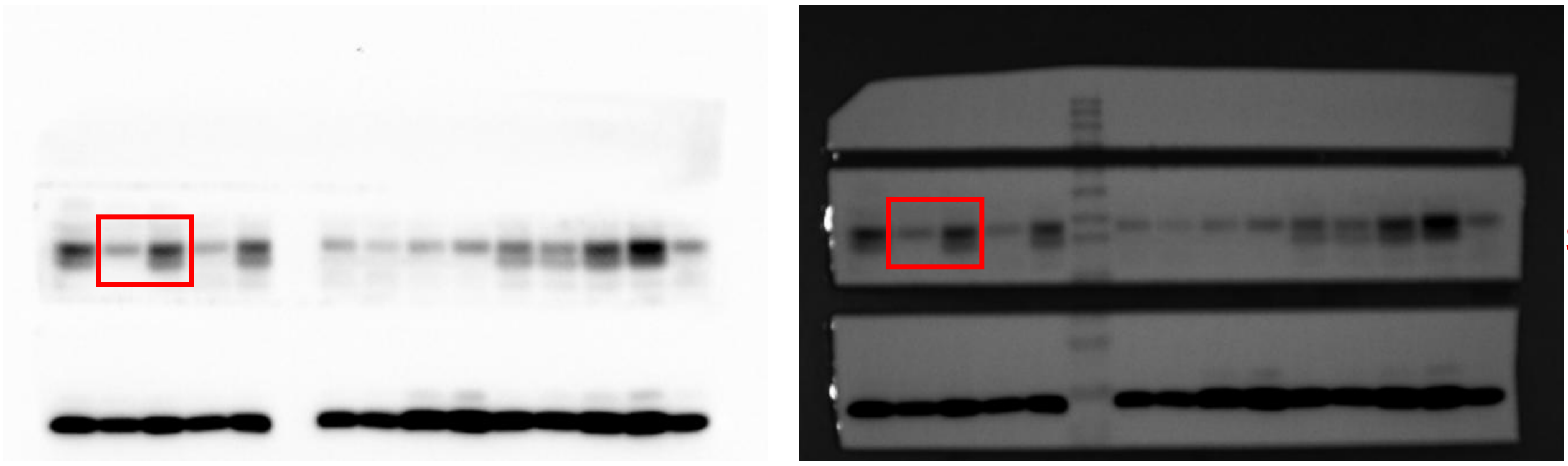

SLC7A11

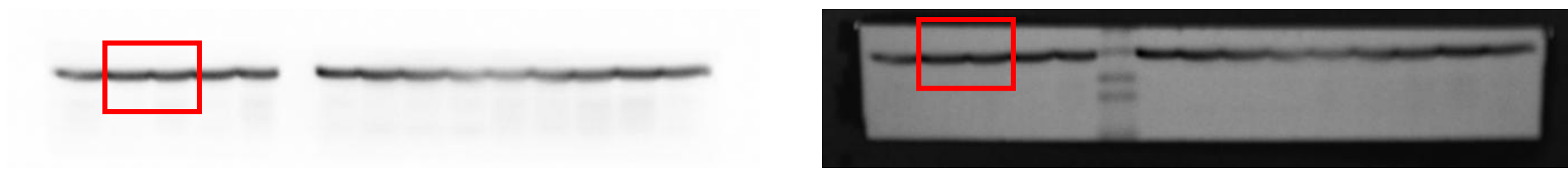

β-Tubulin

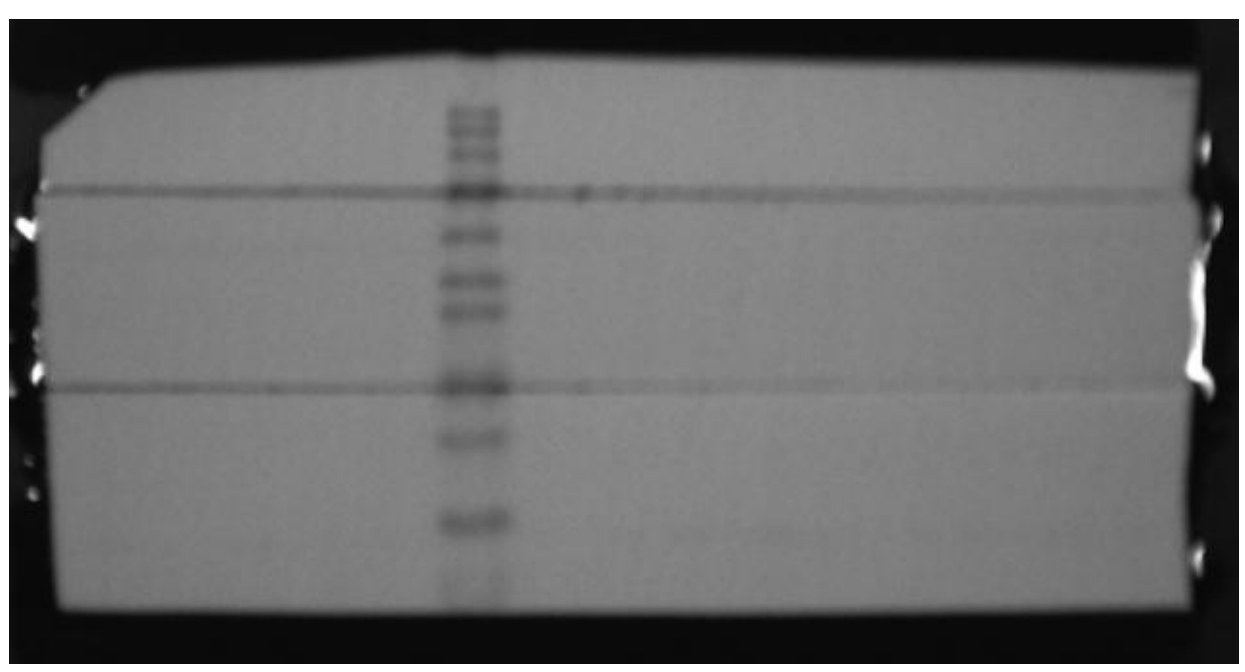

Figure 1P： **ABCB1; β-Tubulin**

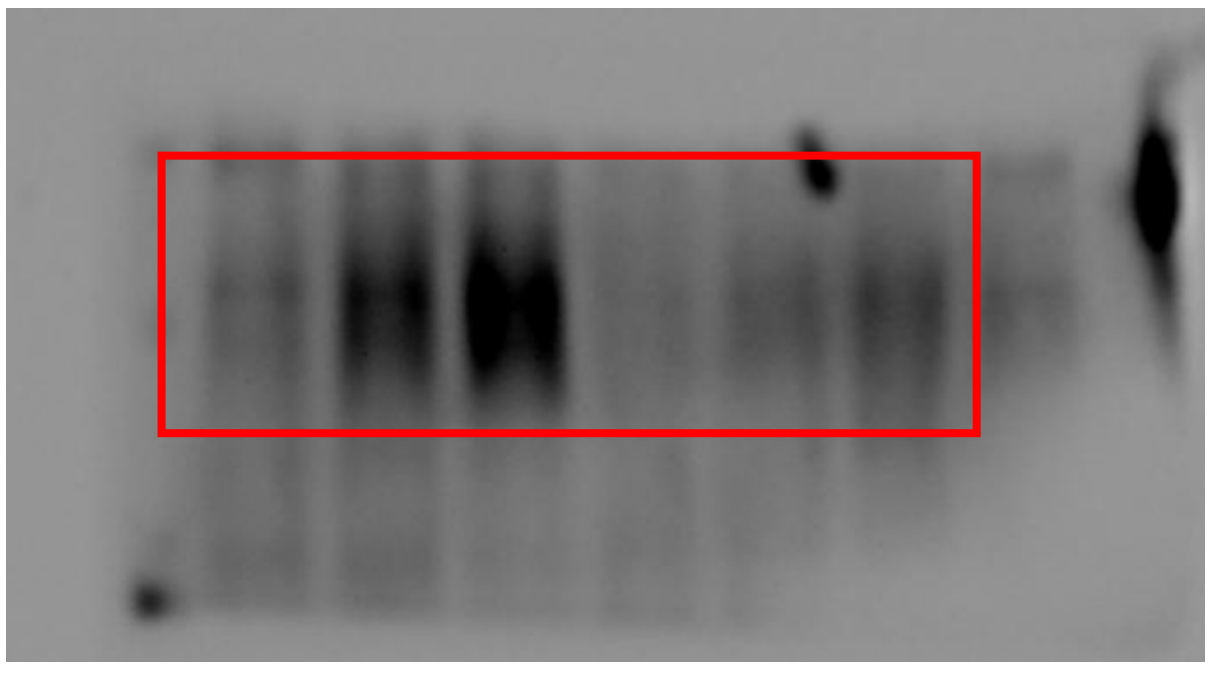

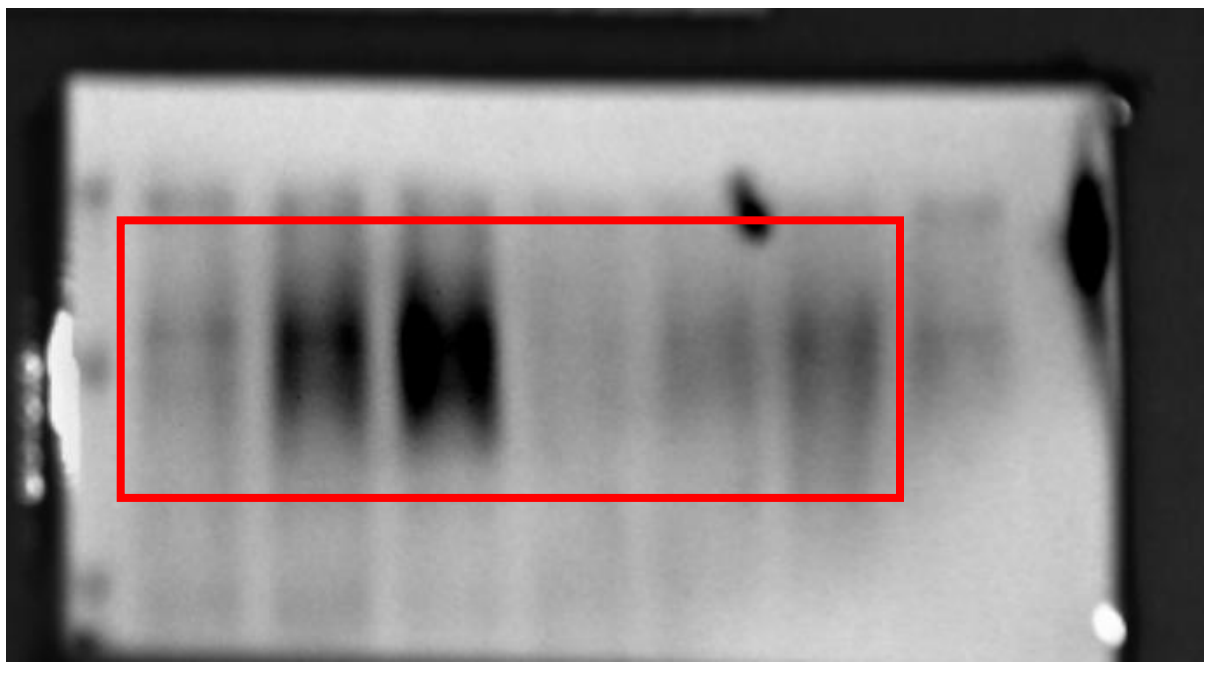

ABCB1

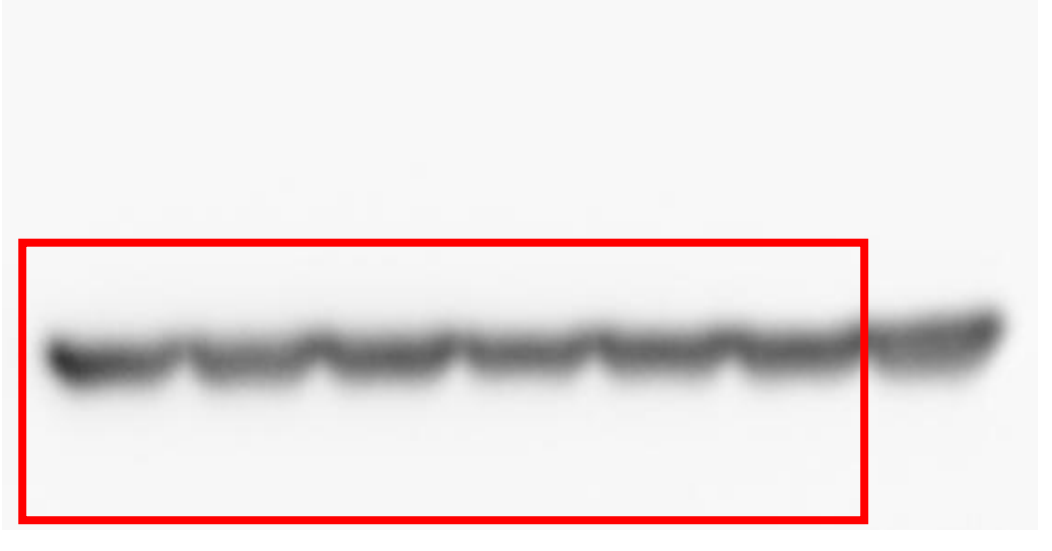

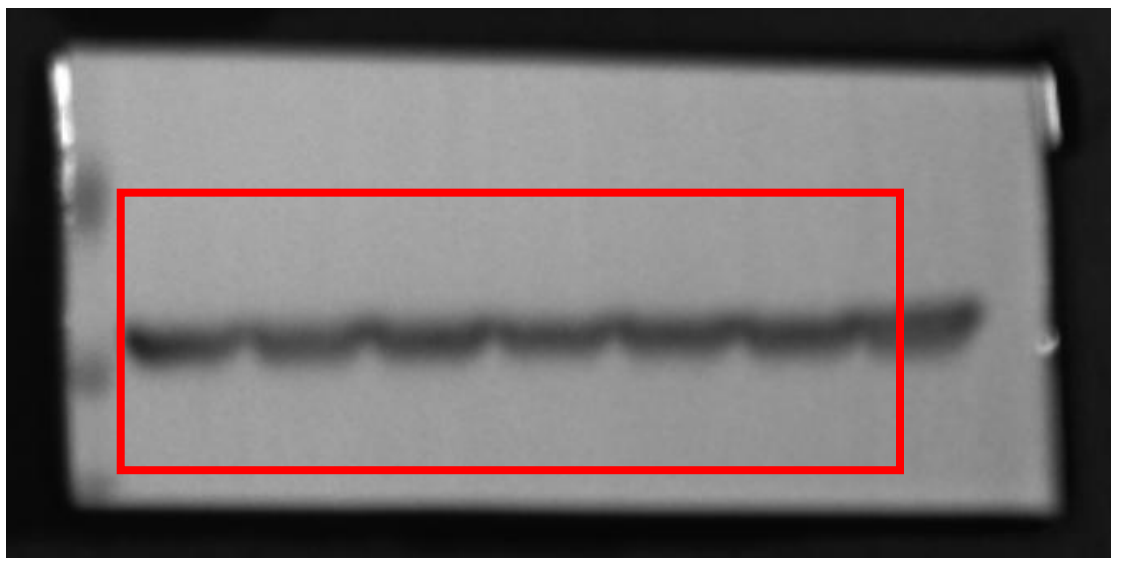

β-Tubulin

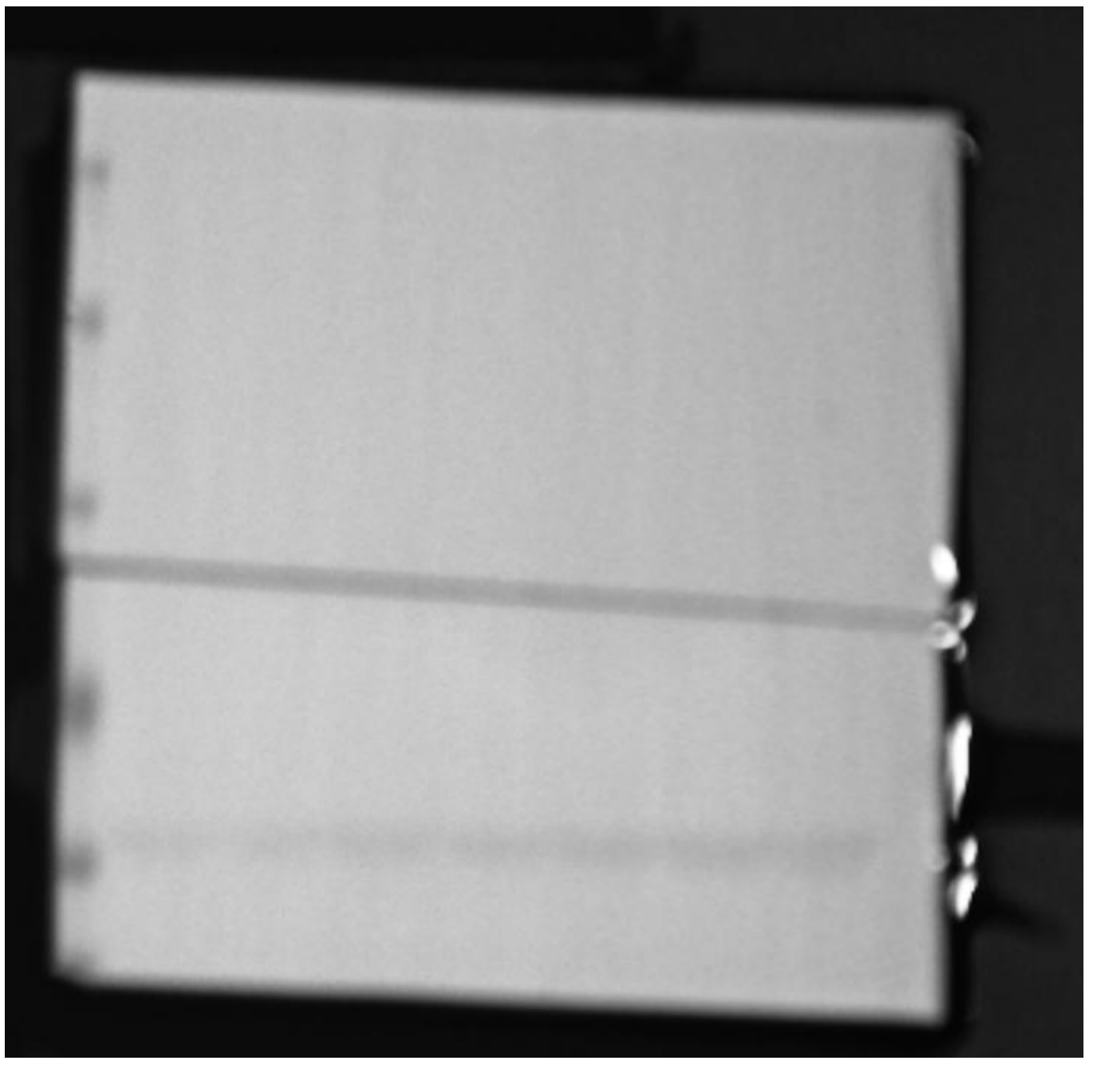

Figure 1Q: γ-H2A.X; β-actin

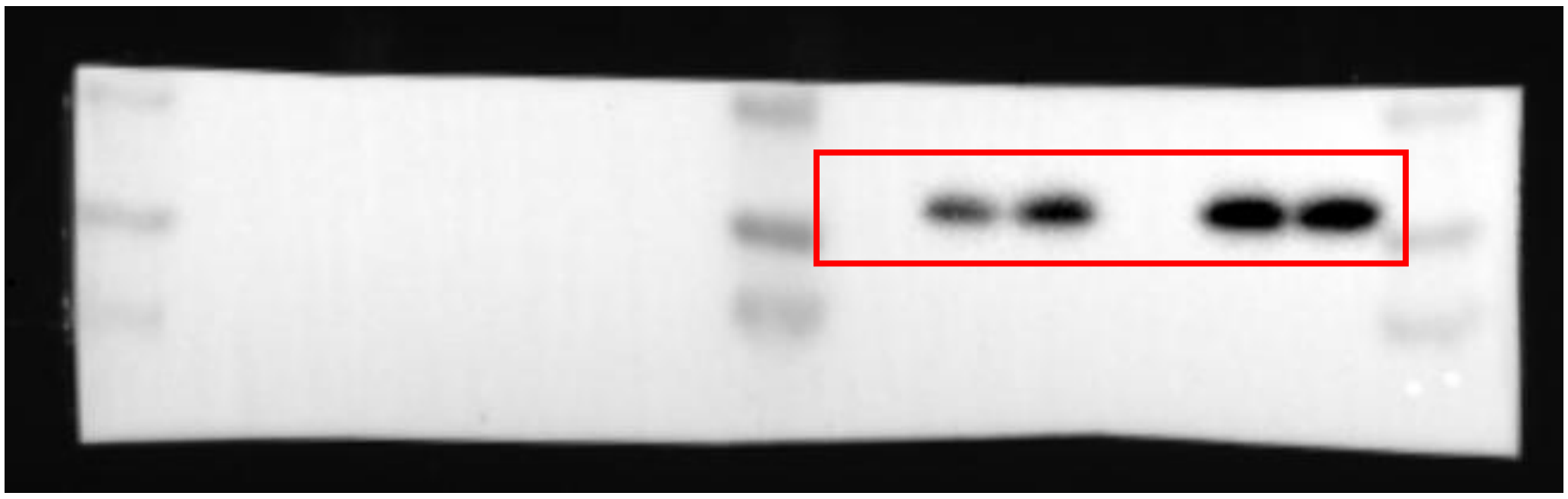

γ-H2A.X

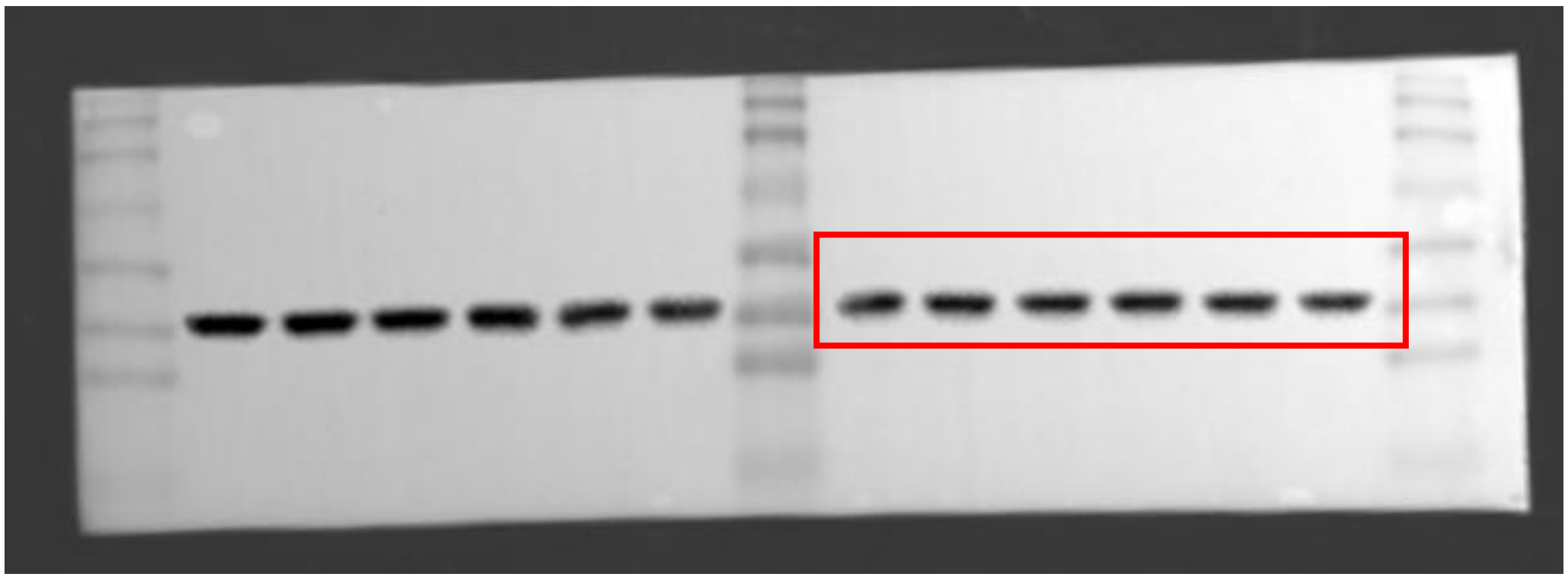

β-actin

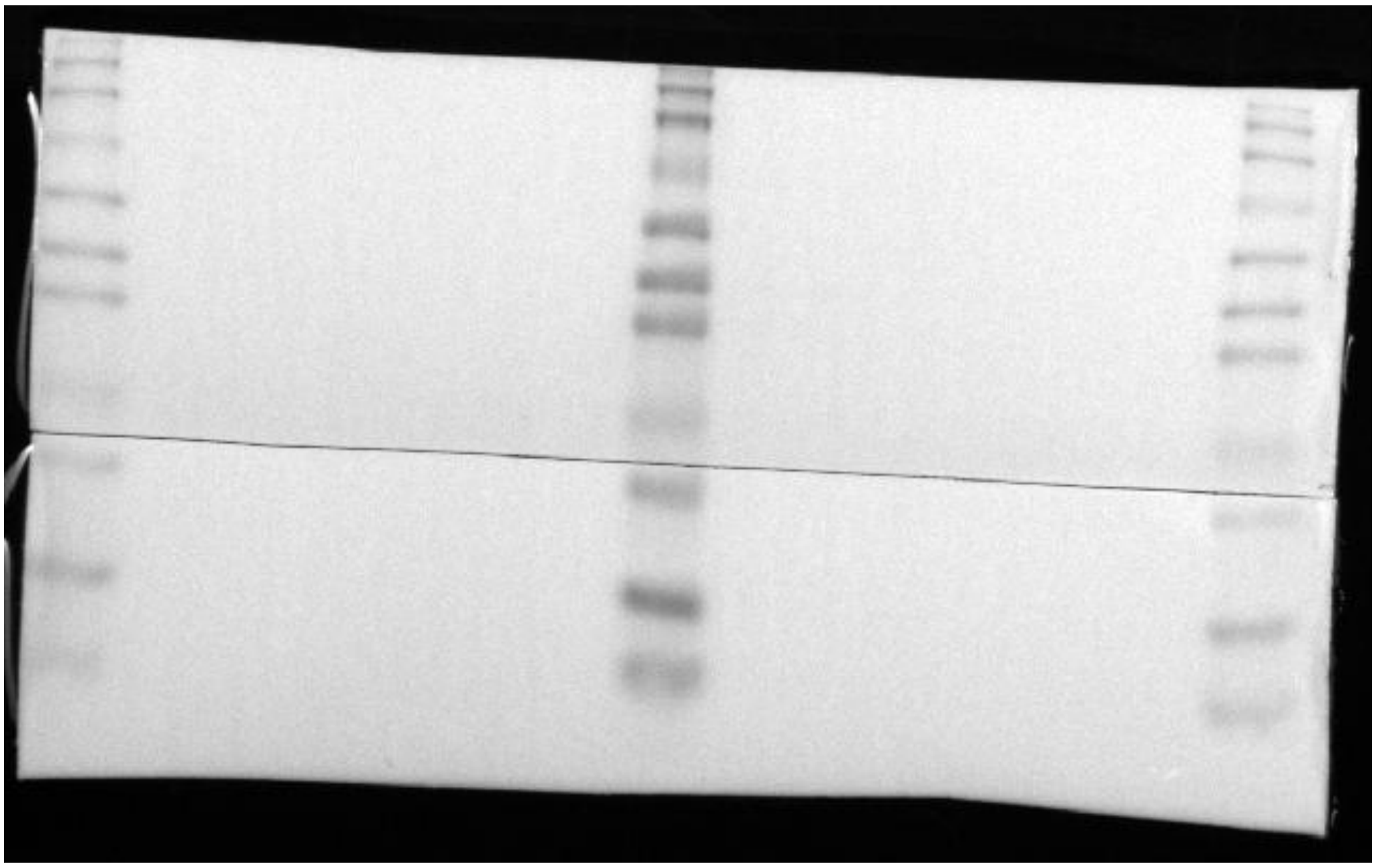

Figure 2D: LC3B; β-Tubulin
Cell: H-R-eGFP vs. H-R-SLC7A11

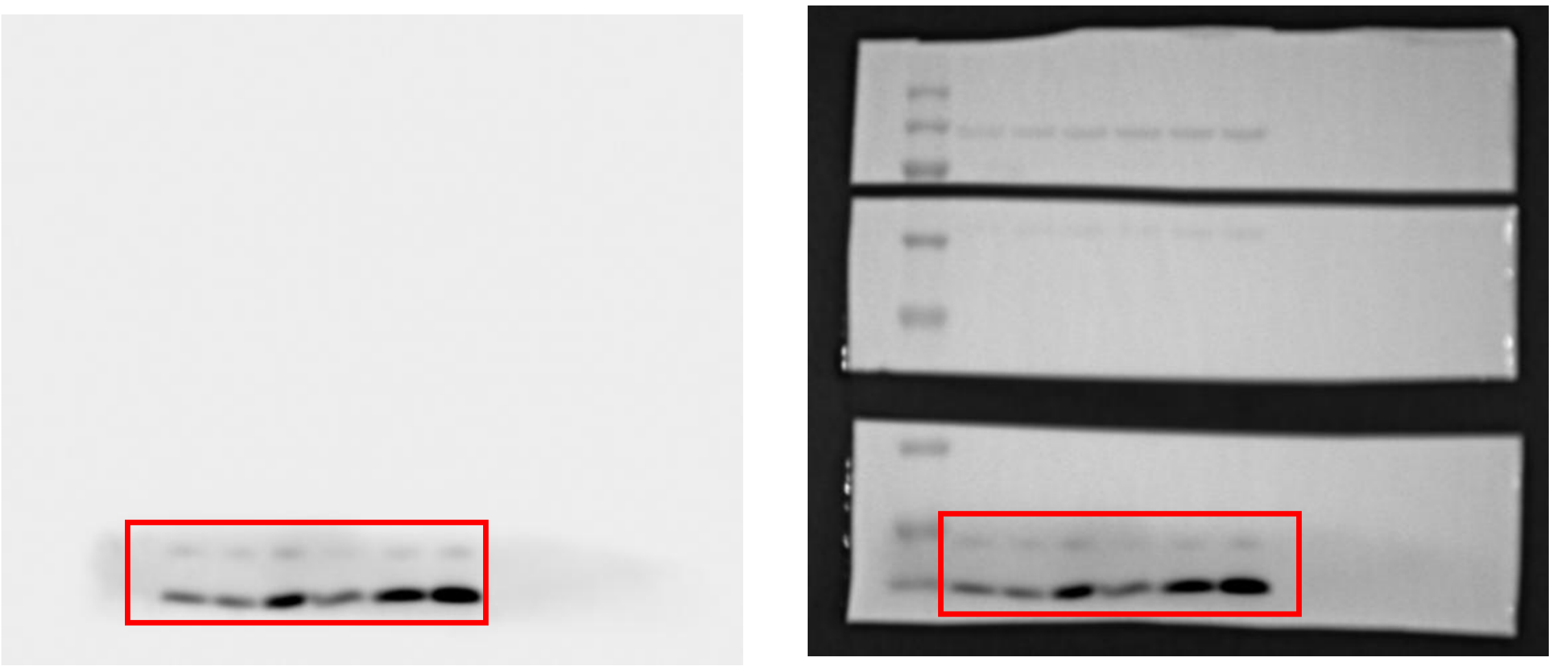

LC3B

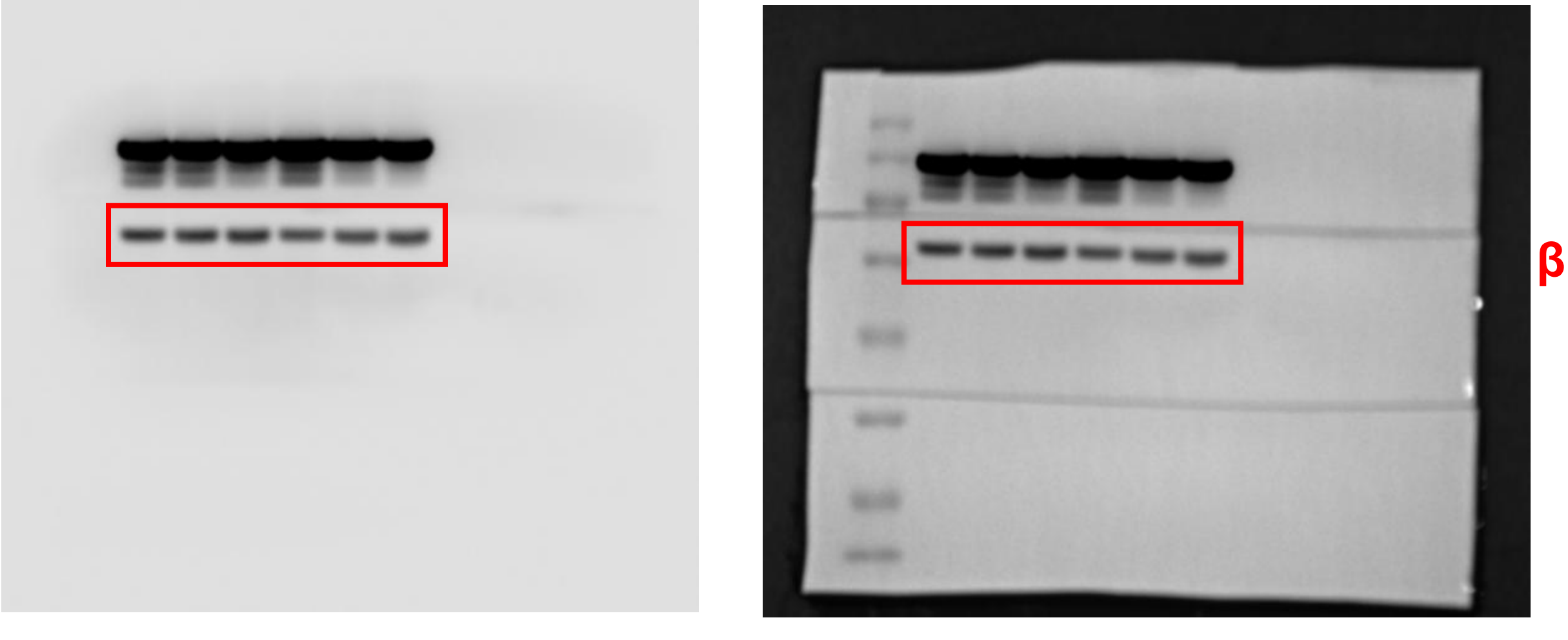

β-Tubulin

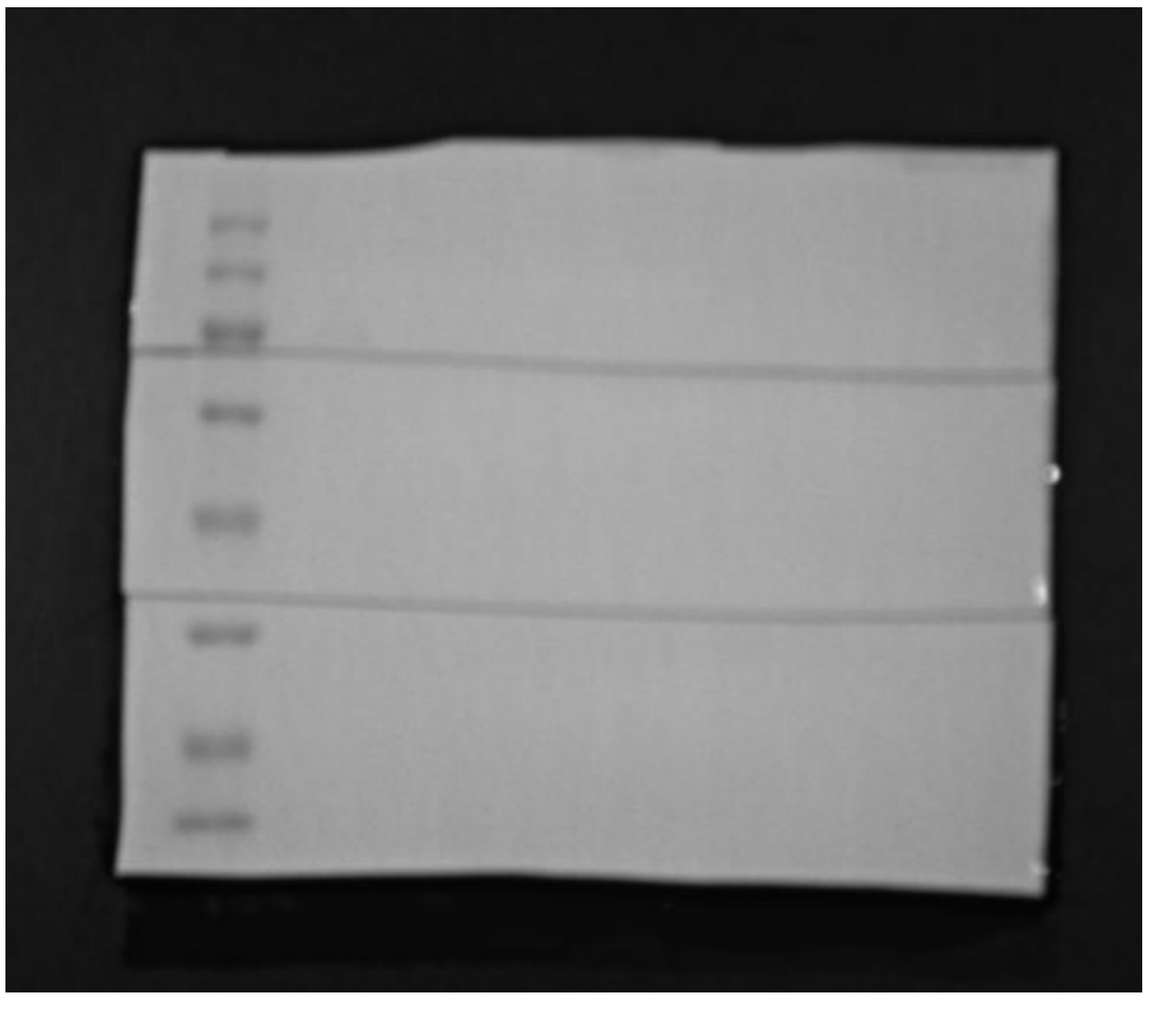

Figure 2D： **LC3B; β-Tubulin**

Cell: H-eGFP vs. H-SLC7A11

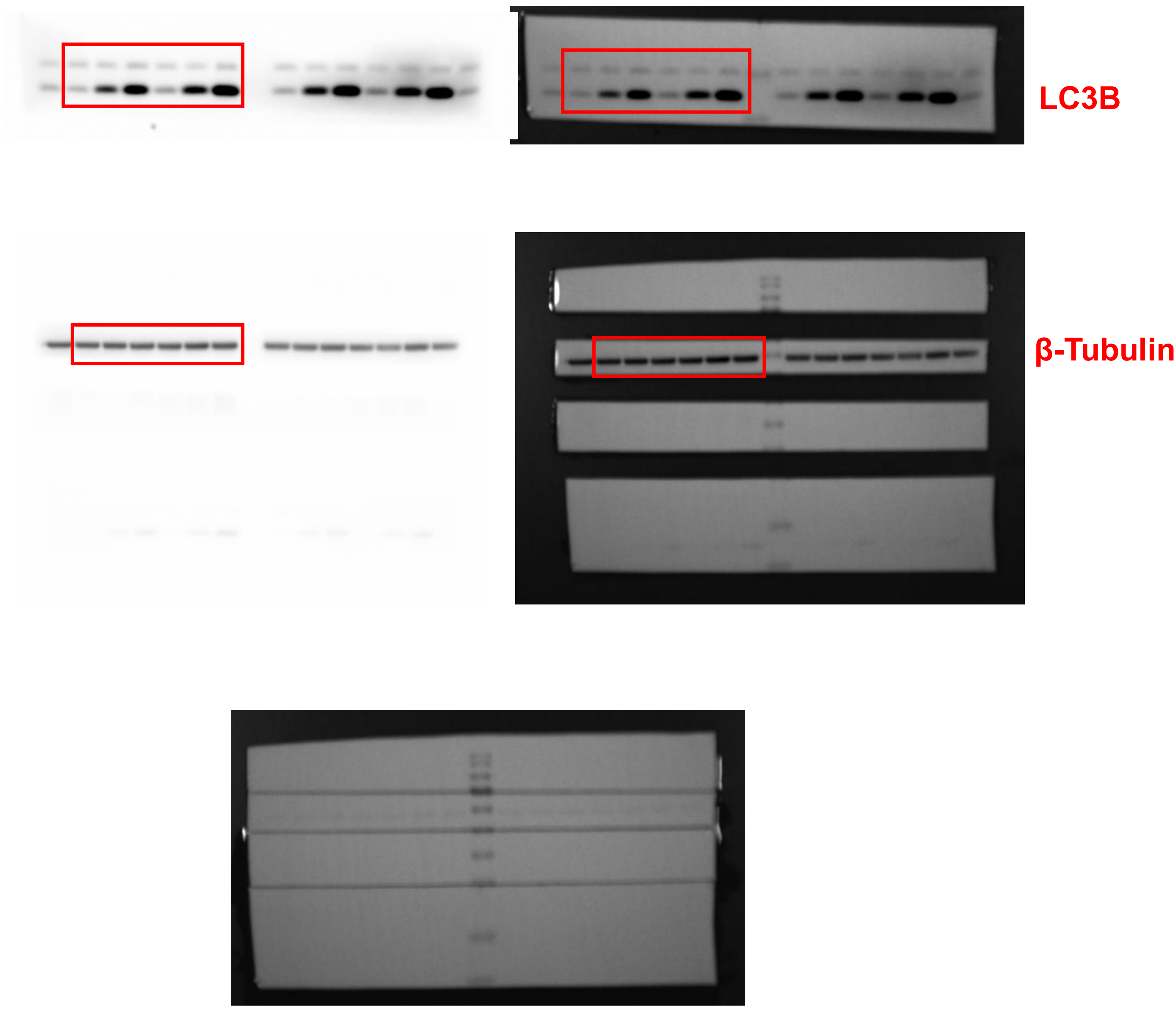

Figure 2D: LAMP1; β-Tubulin

Cell: H-R-eGFP vs. H-R-SLC7A11

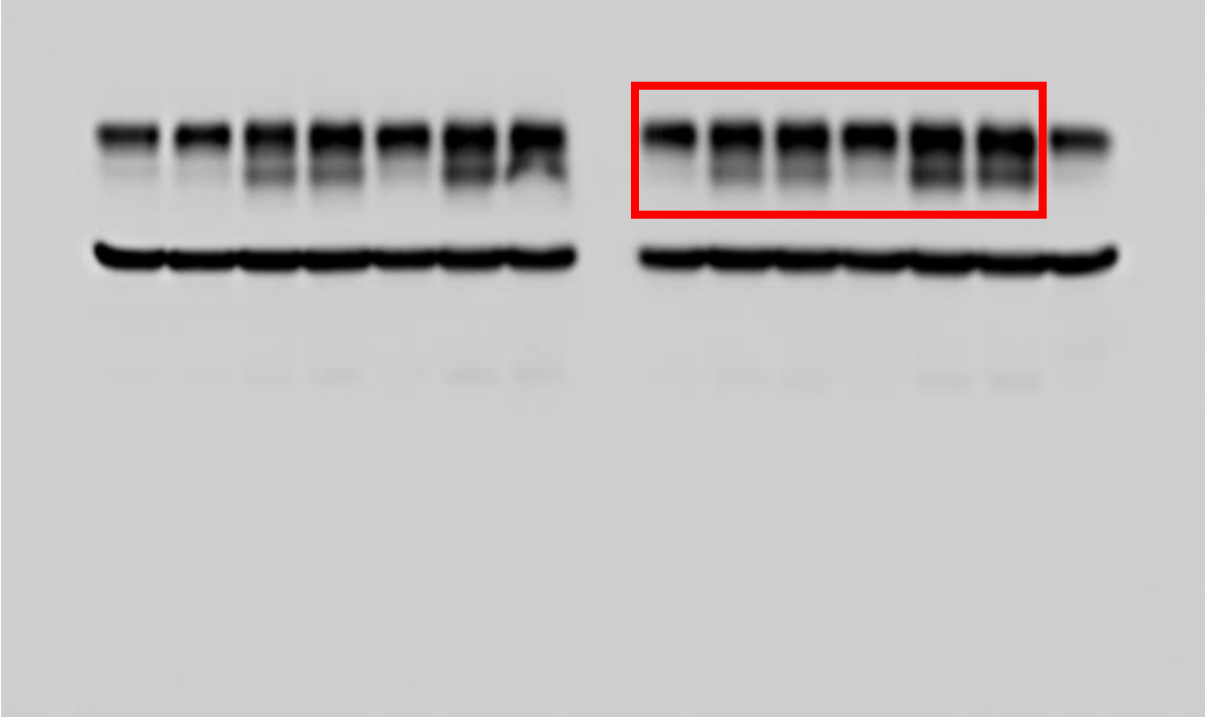

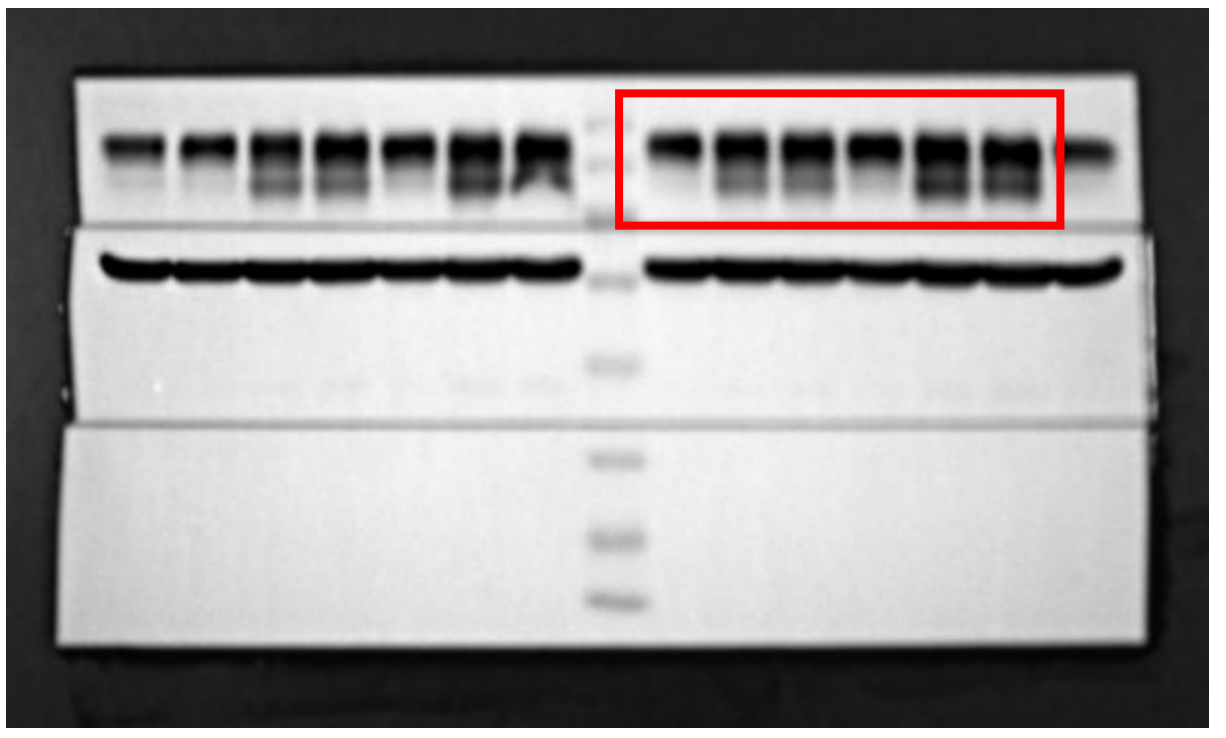

LAMP1

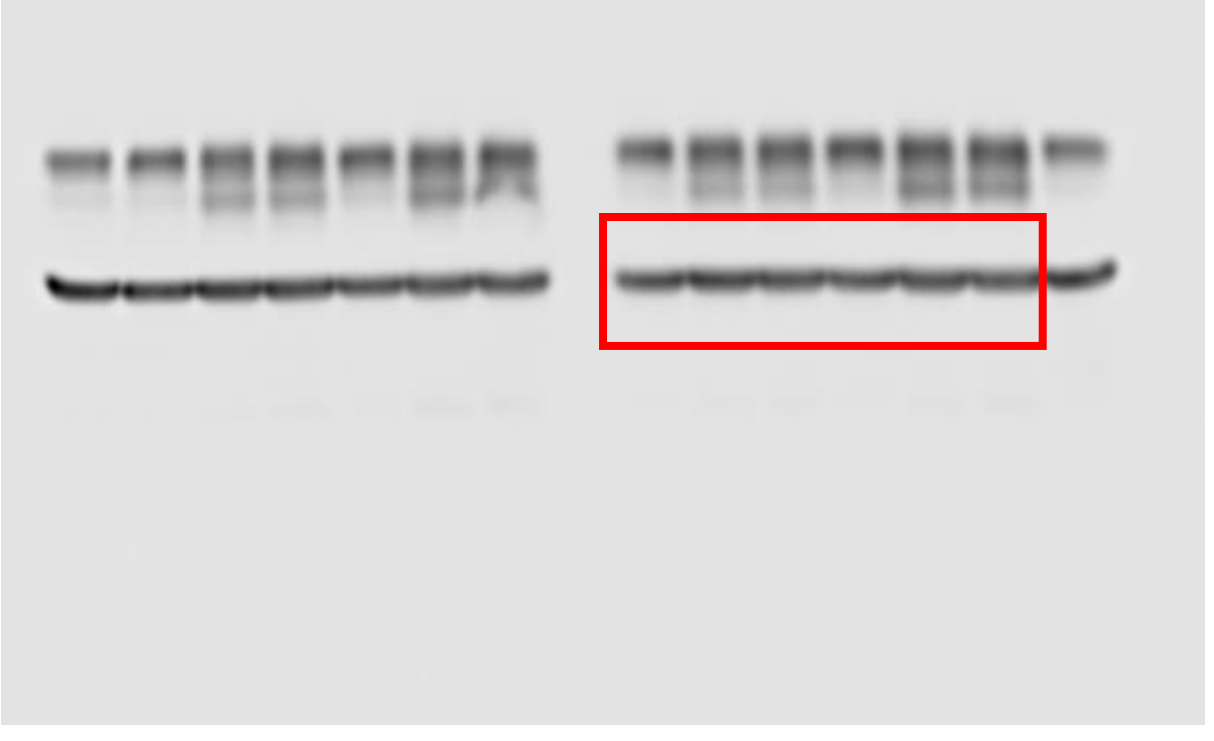

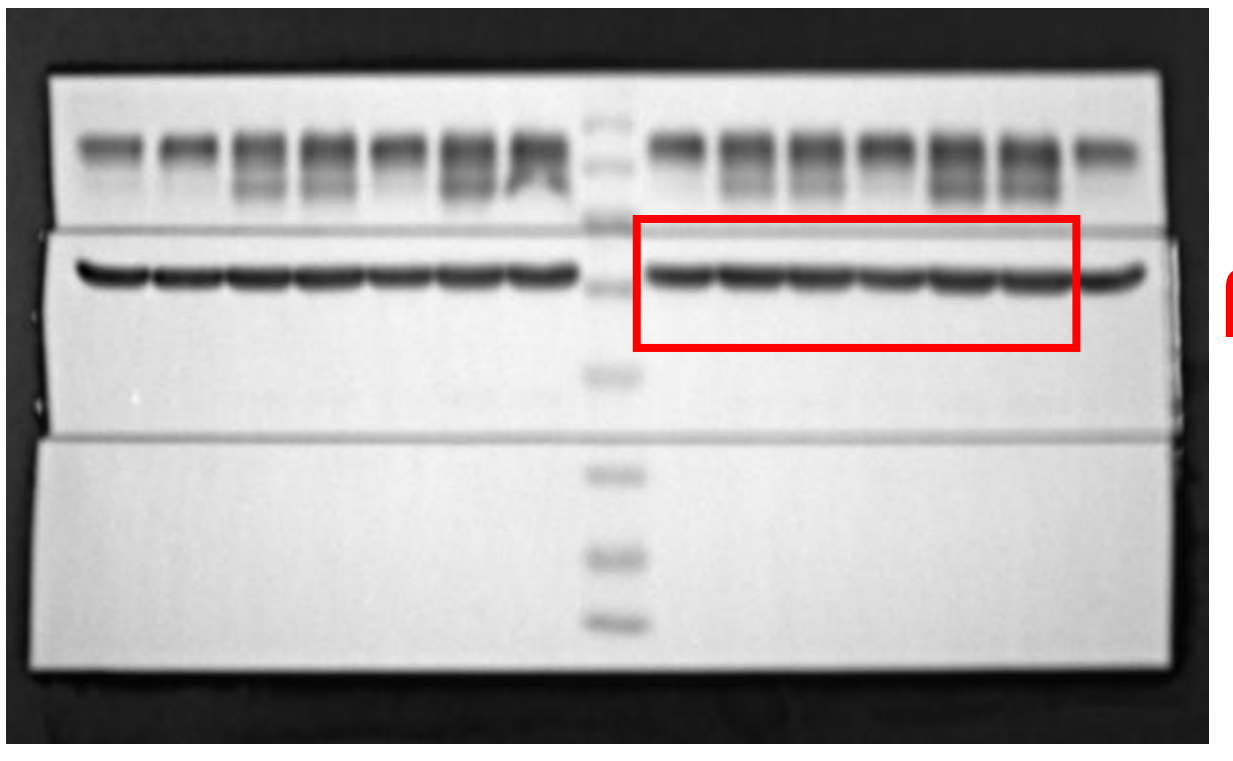

β-Tubulin

Figure 2D： **LAMP1; β-Tubulin**

Cell: H-eGFP vs. H-SLC7A11

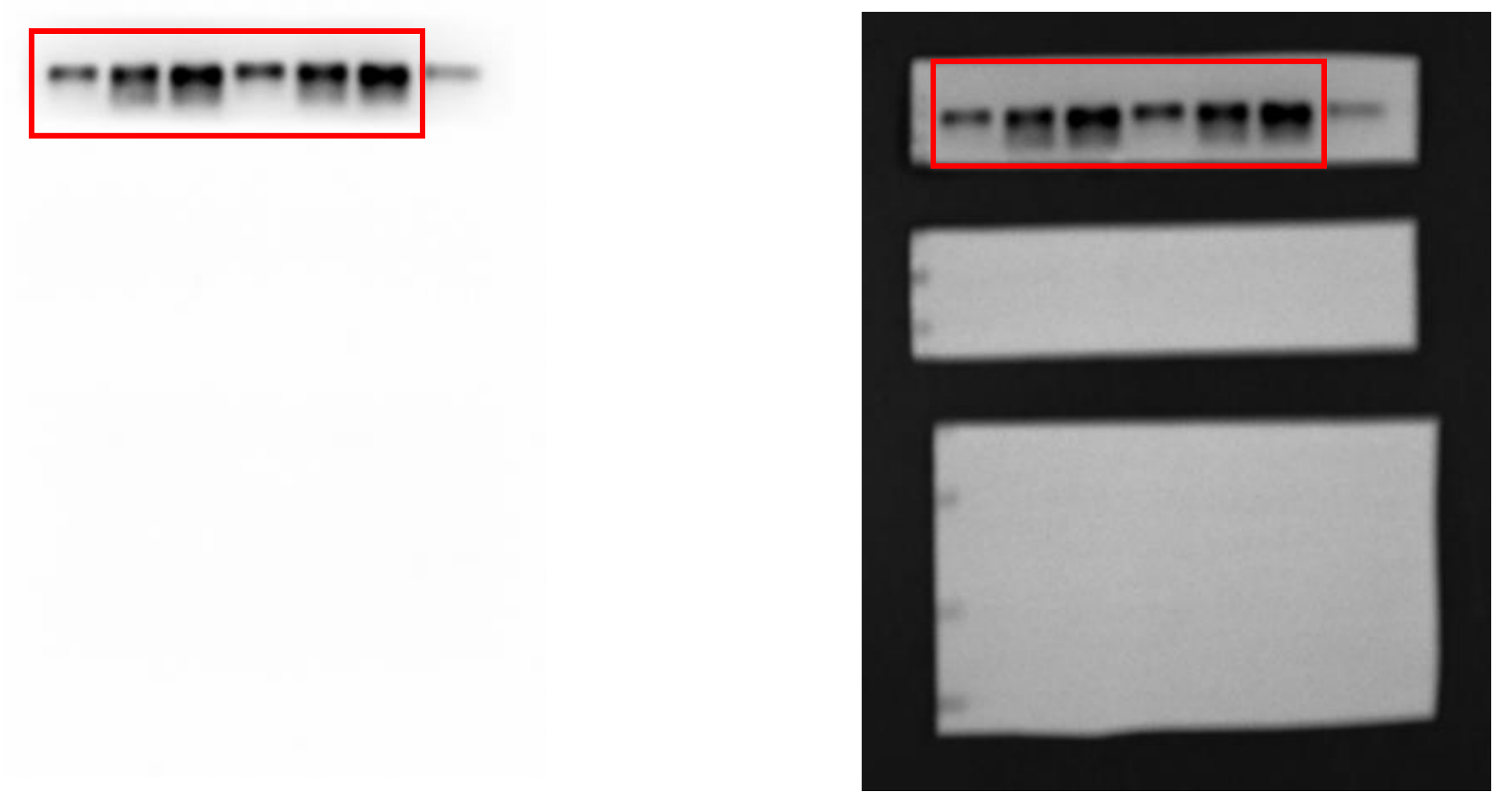

LAMP1

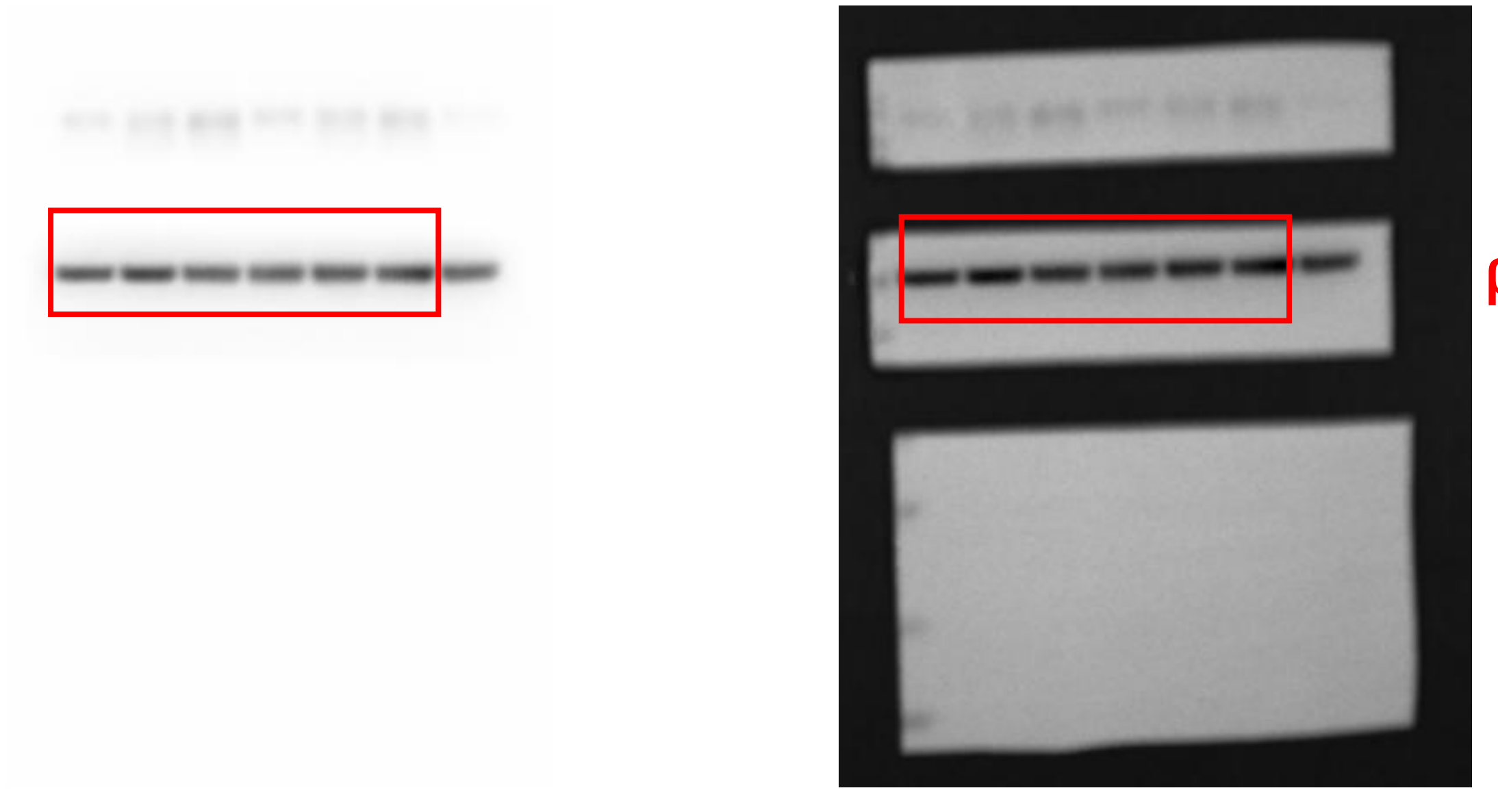

β-Tubulin

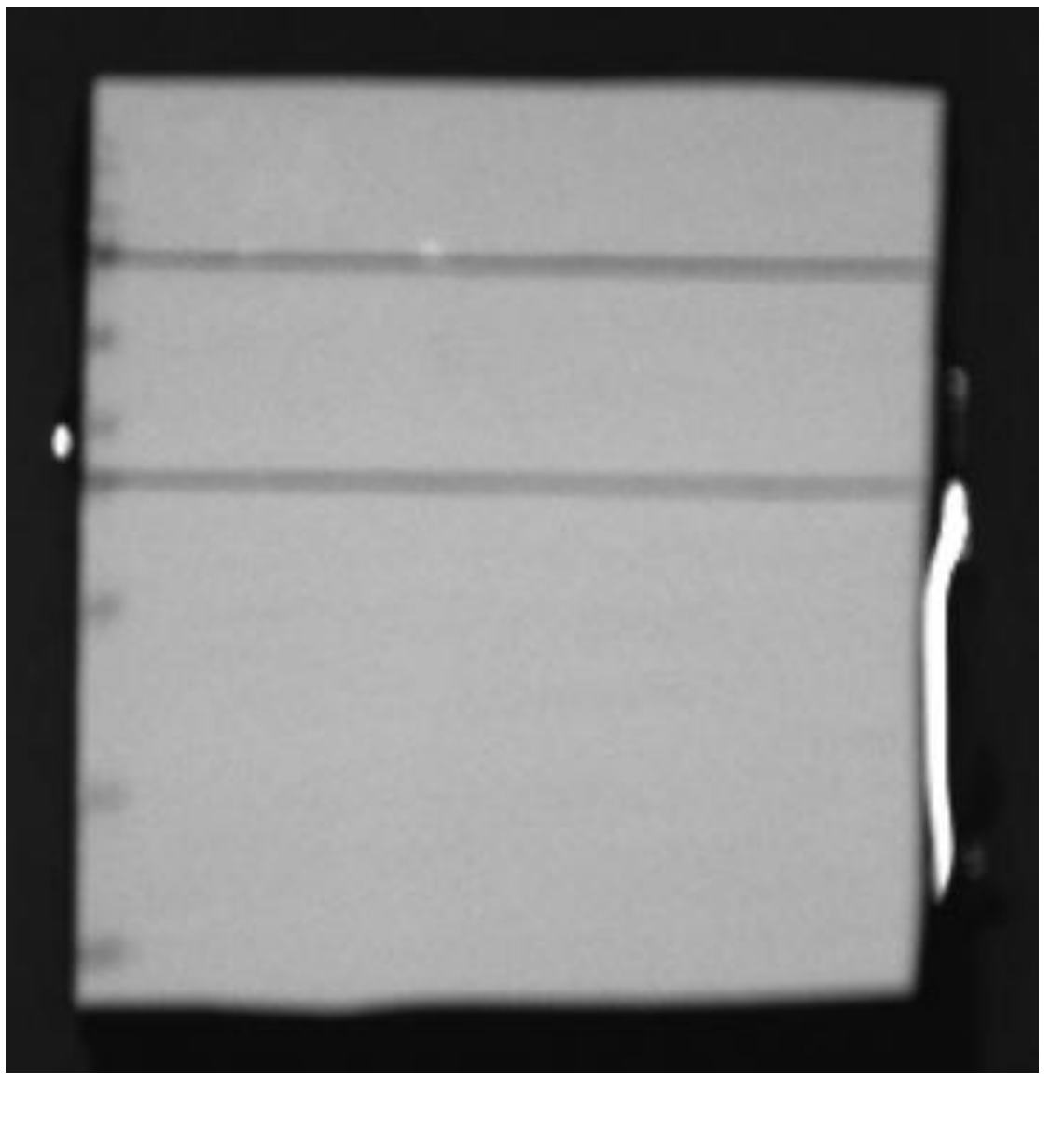

Figure 2F： PI3K p110a; mTOR

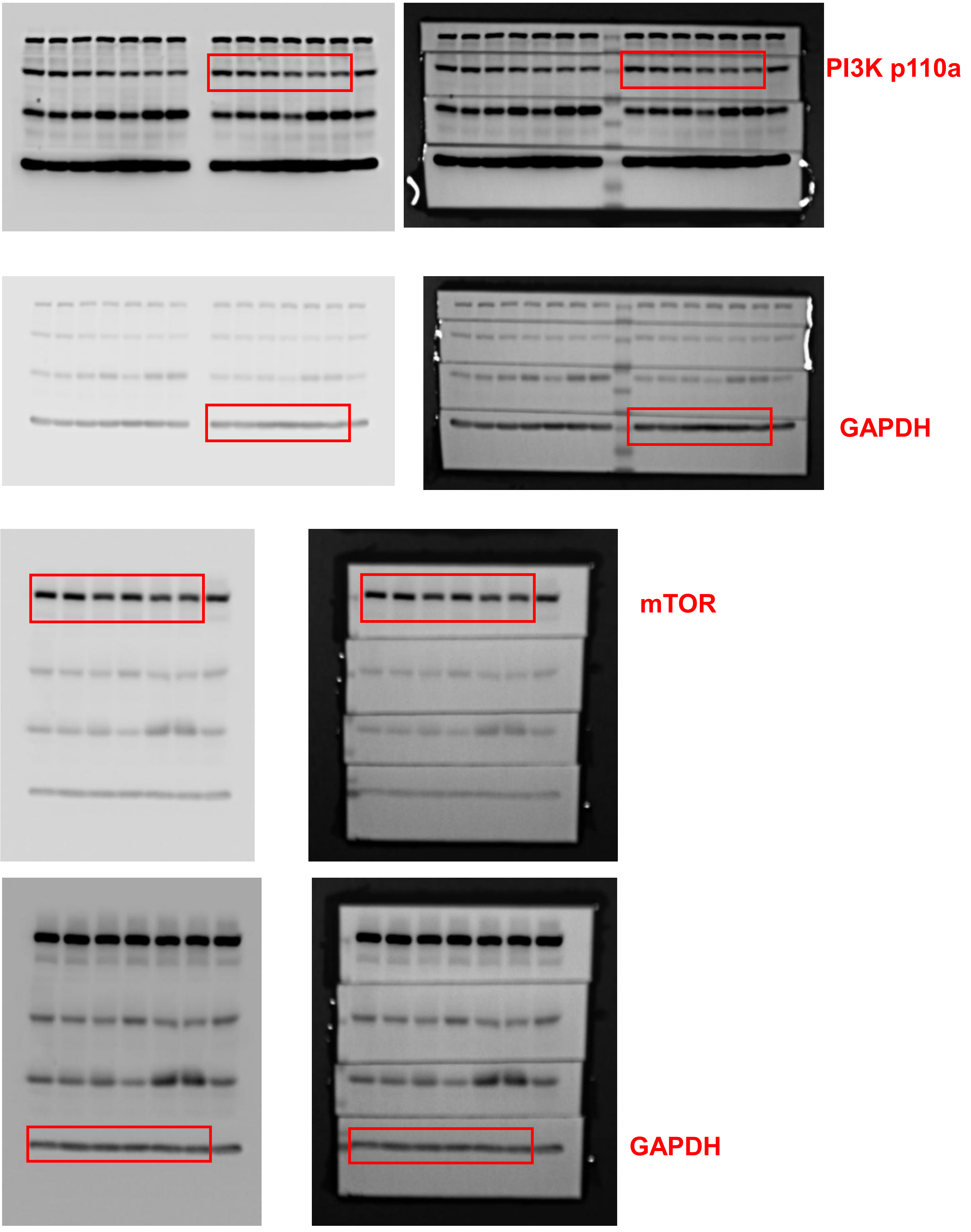

Figure 2F： p-mTOR; β-actin

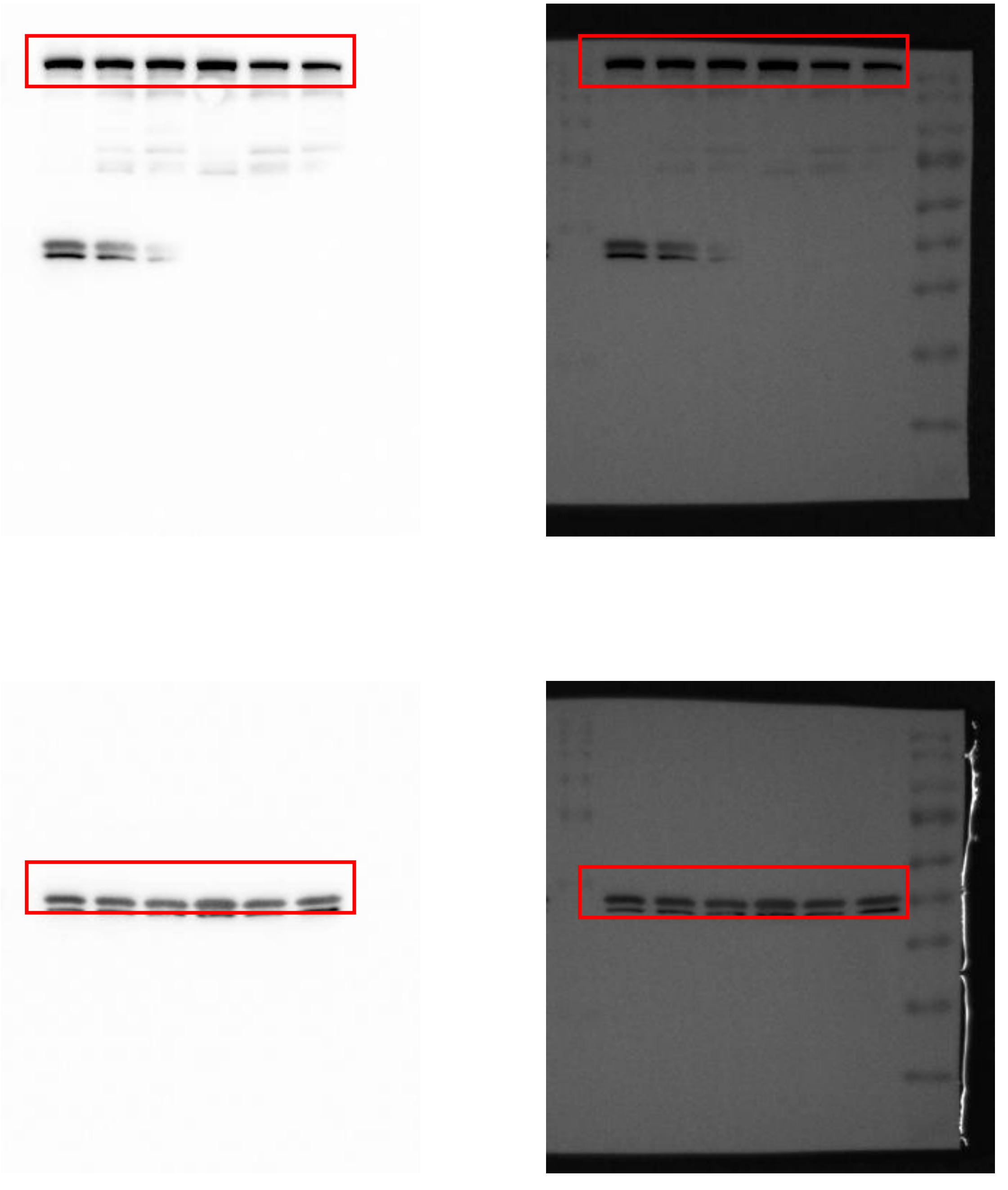

p-mTOR

β-actin

Figure 2F：Beclin-1

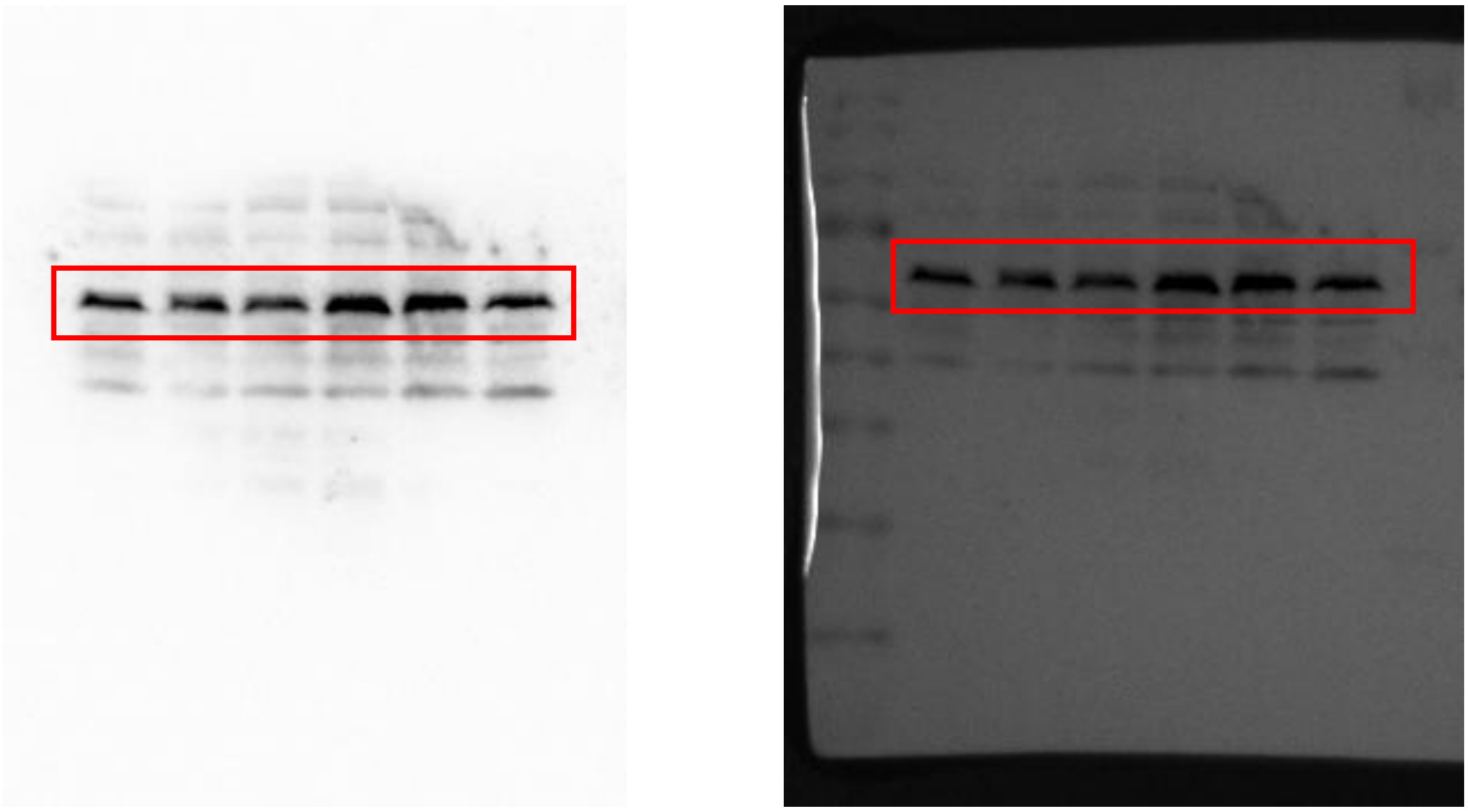

Beclin-1

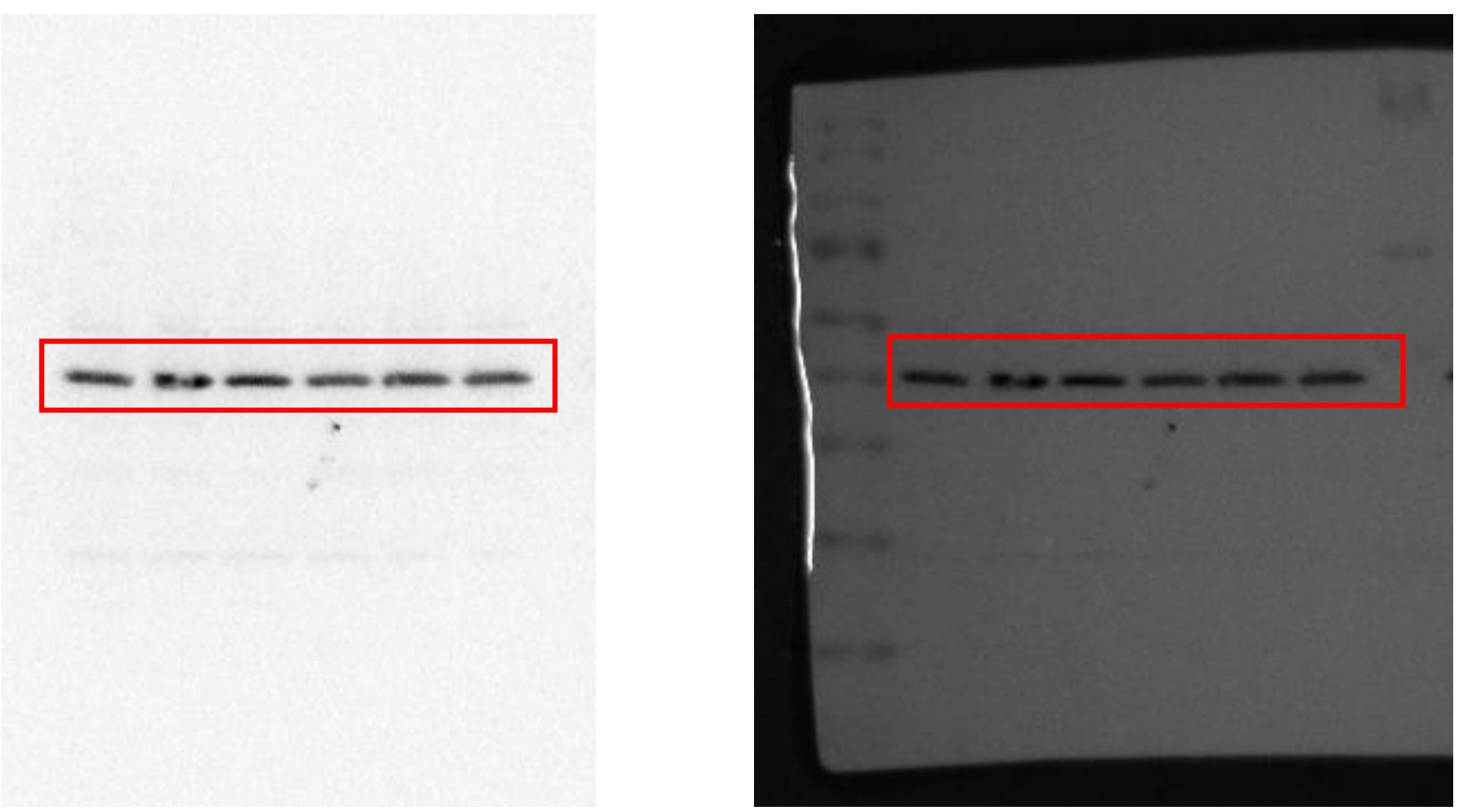

β-actin

Figure 2F：Rab7

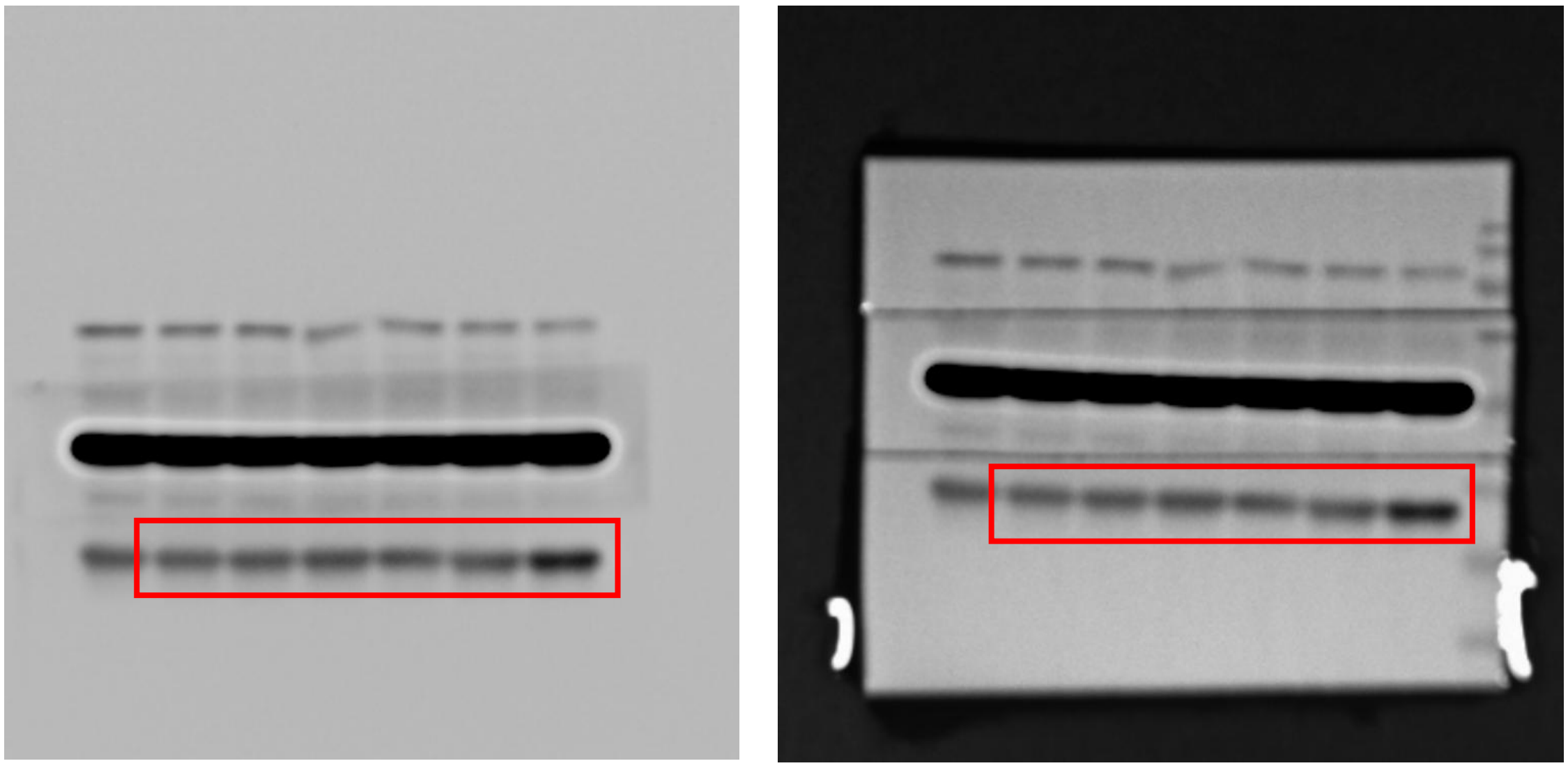

Rab7

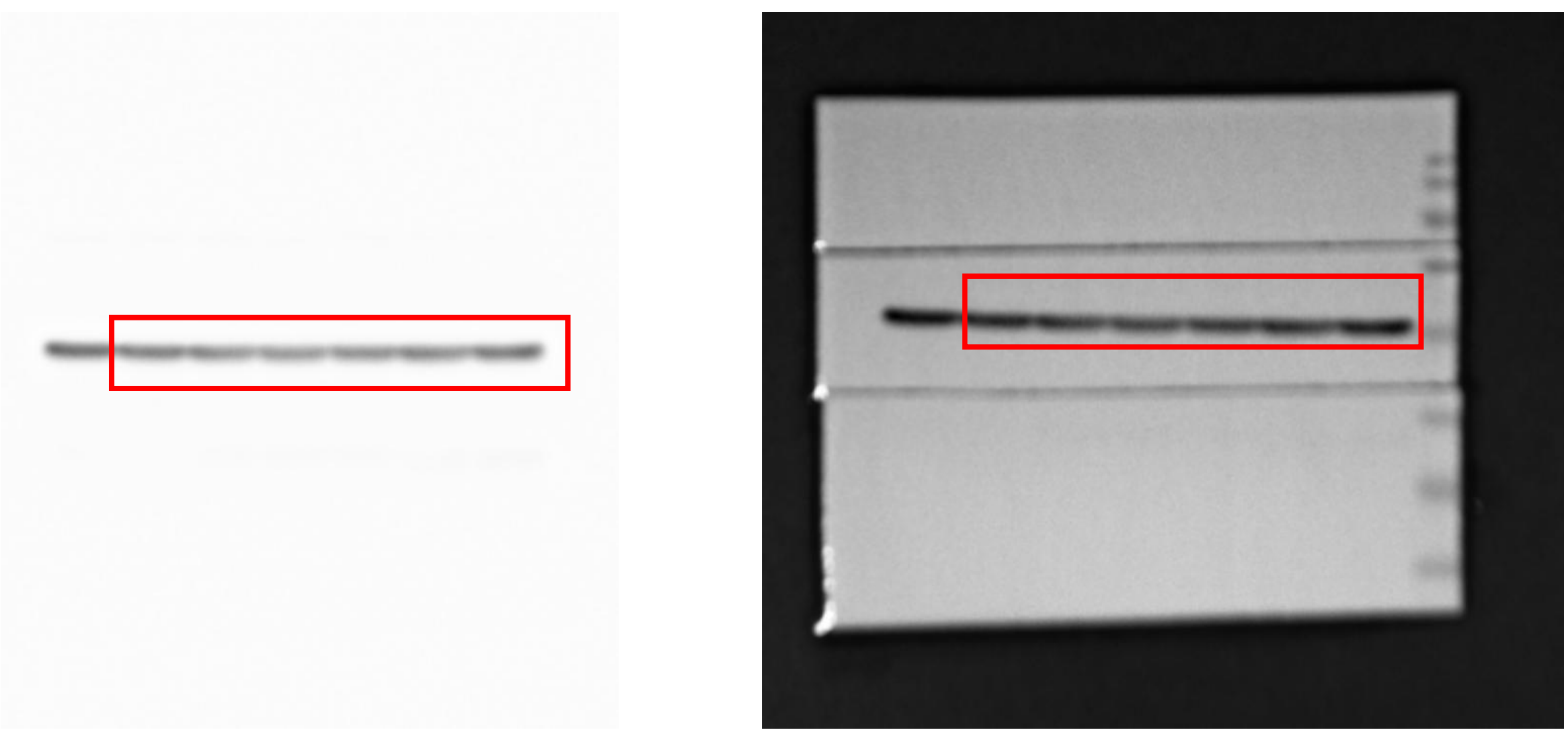

GAPDH

Figure 3G： SLC7A11; LC3B; LAMP1; β-actin

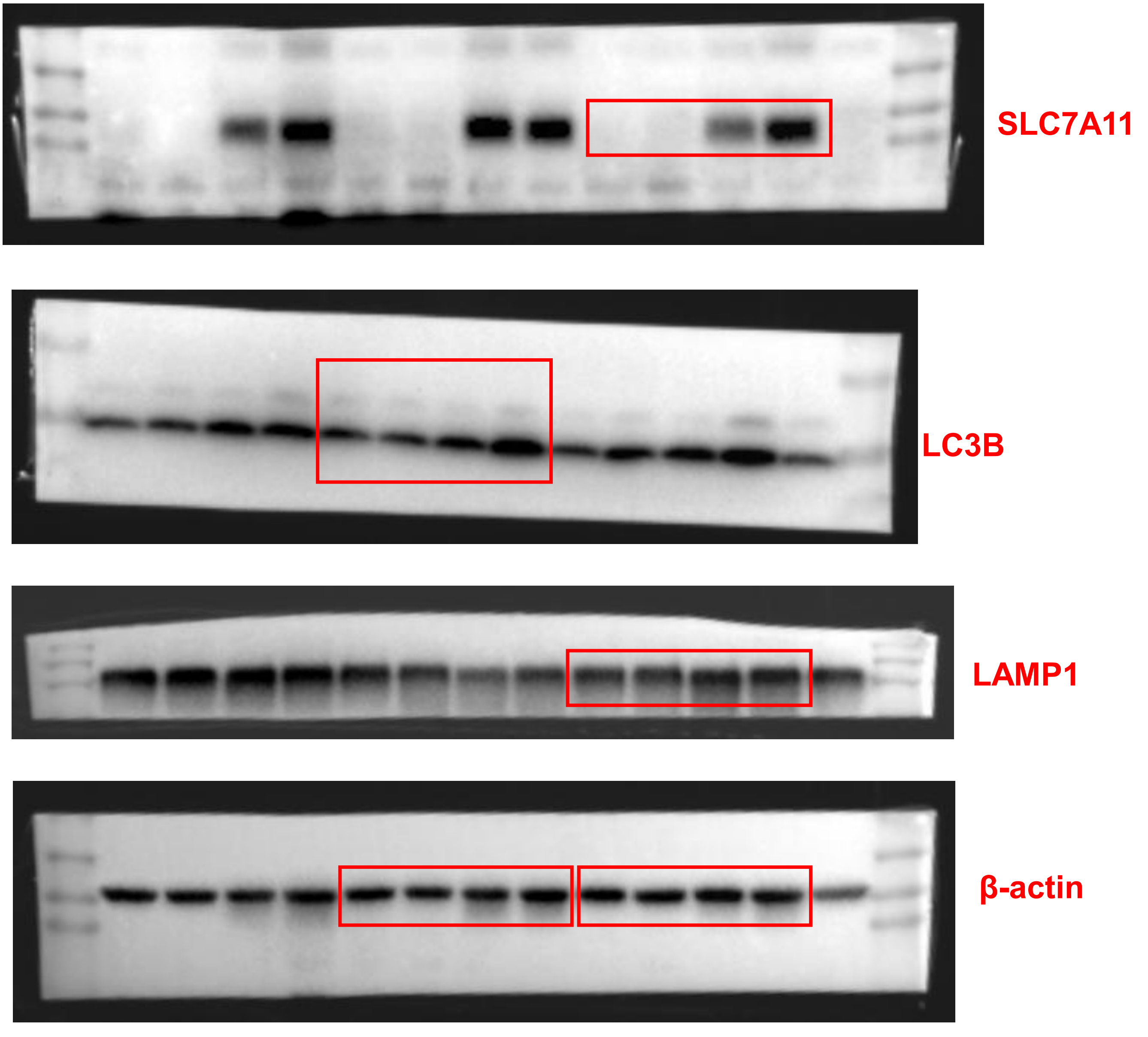

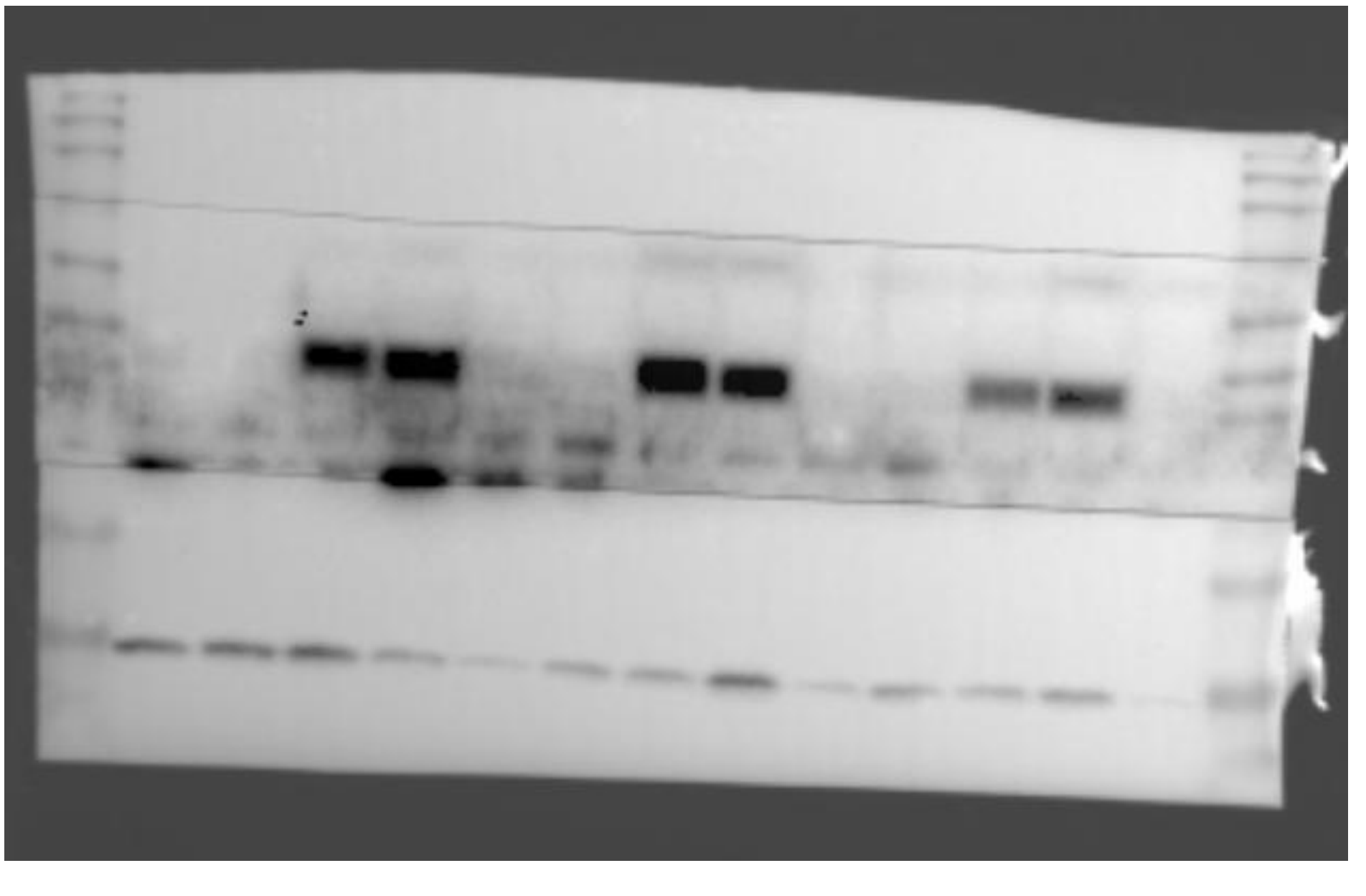

Figure 6J: KLF2; β-actin

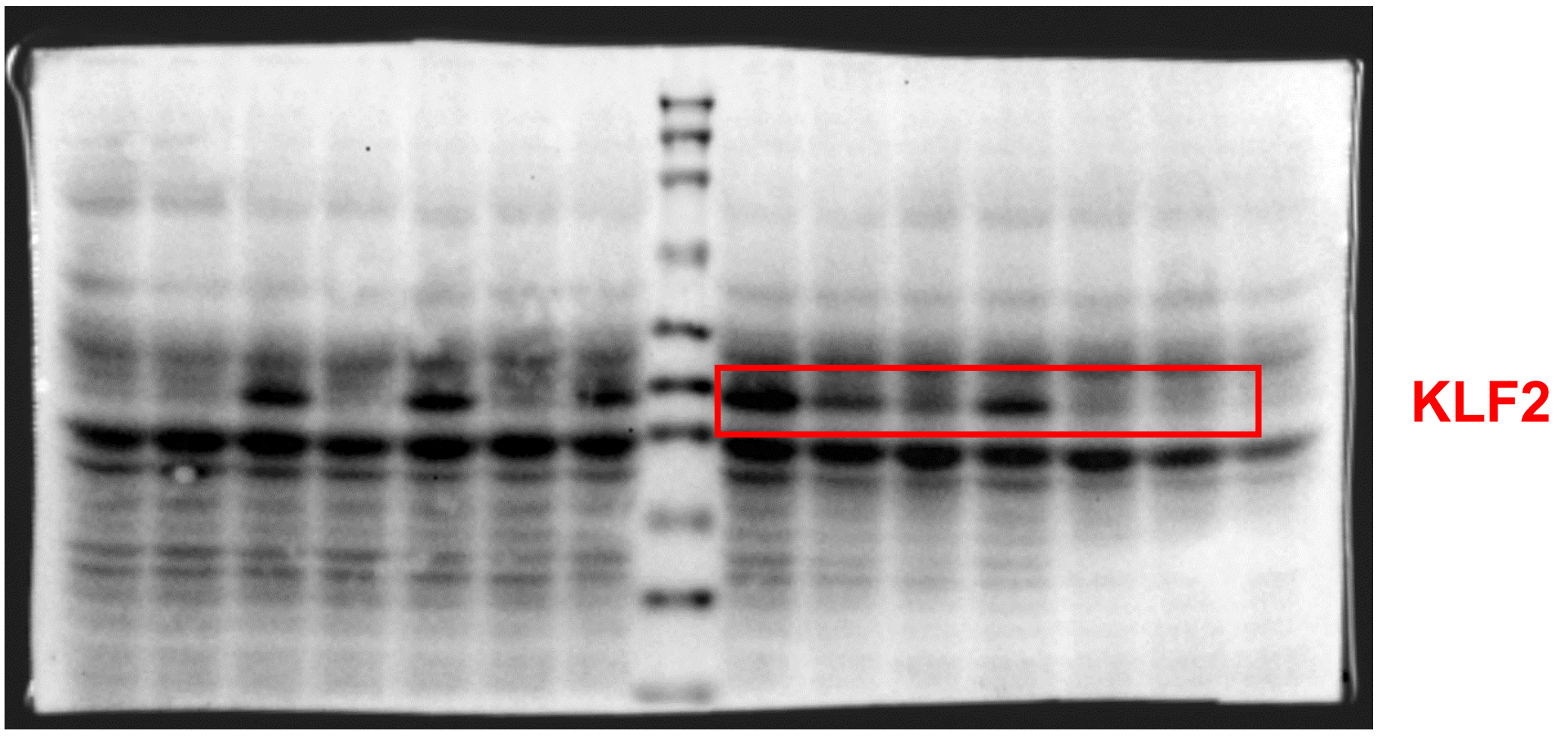

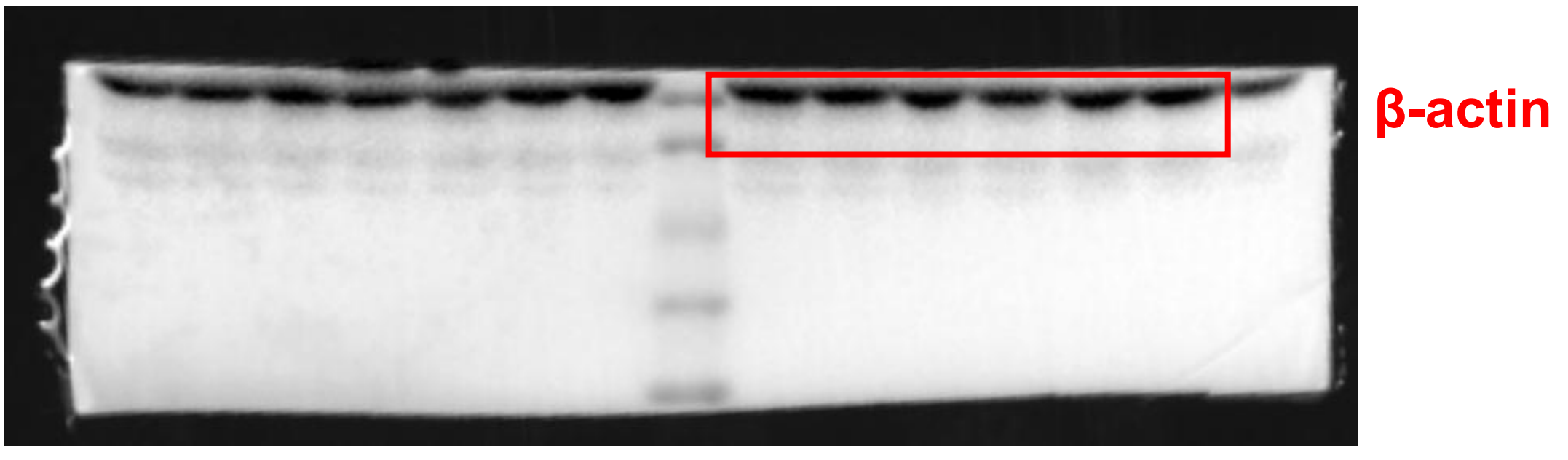

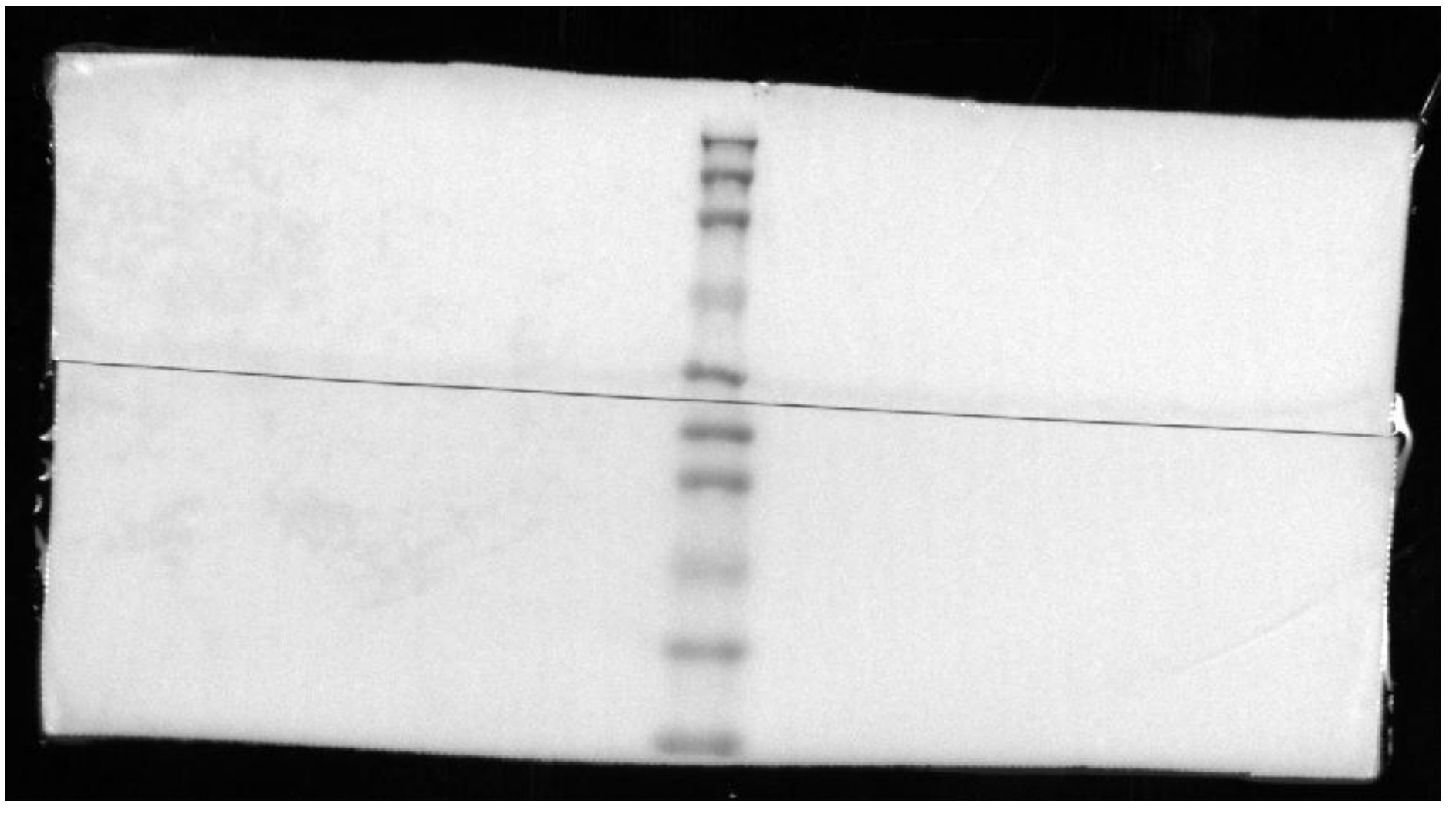

Figure 6J： **TFCP2; GAPDH**

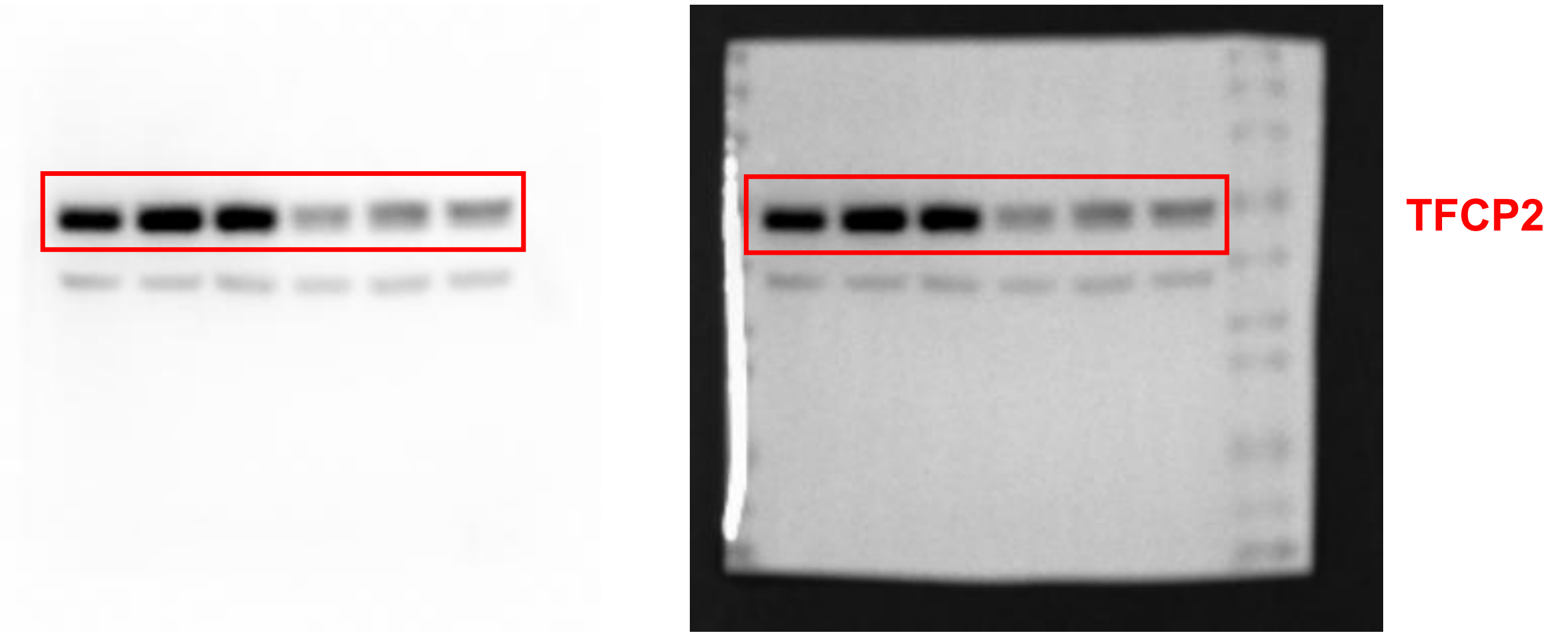

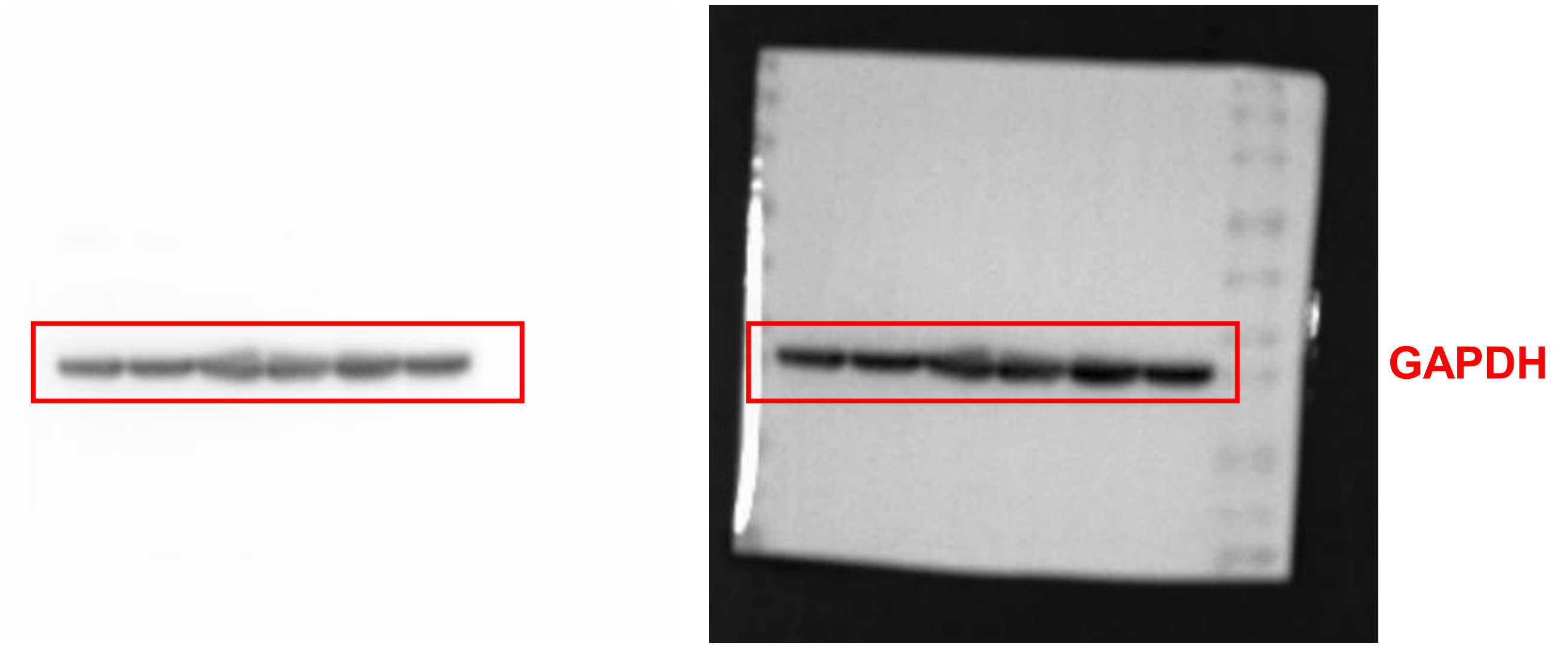

Figure 6L: KLF2; β-actin

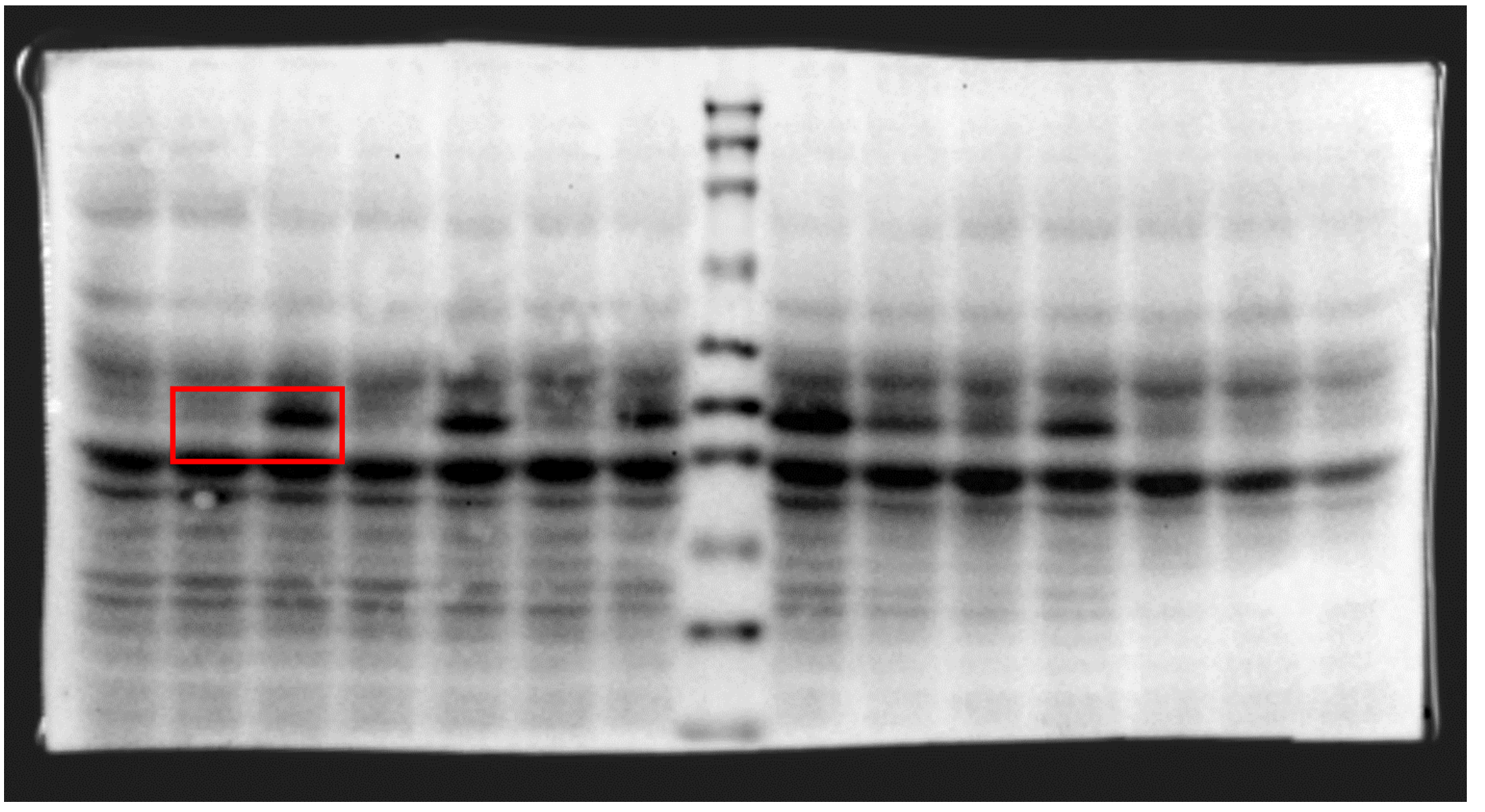

KLF2

β-actin

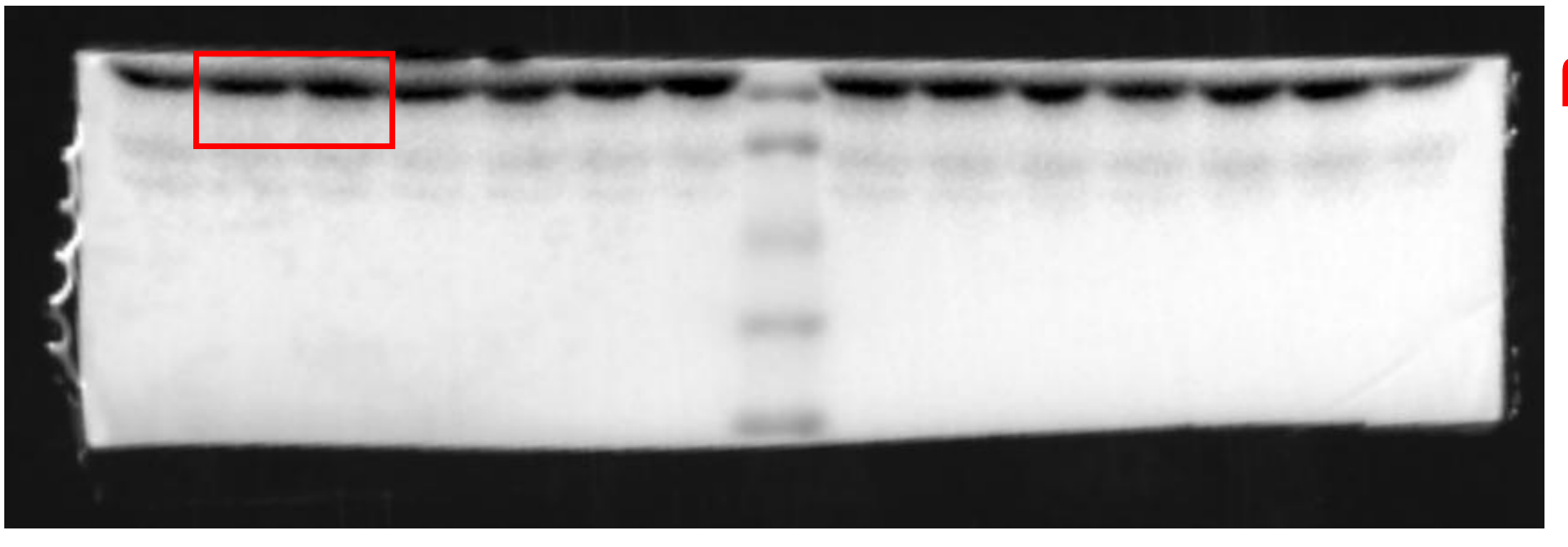

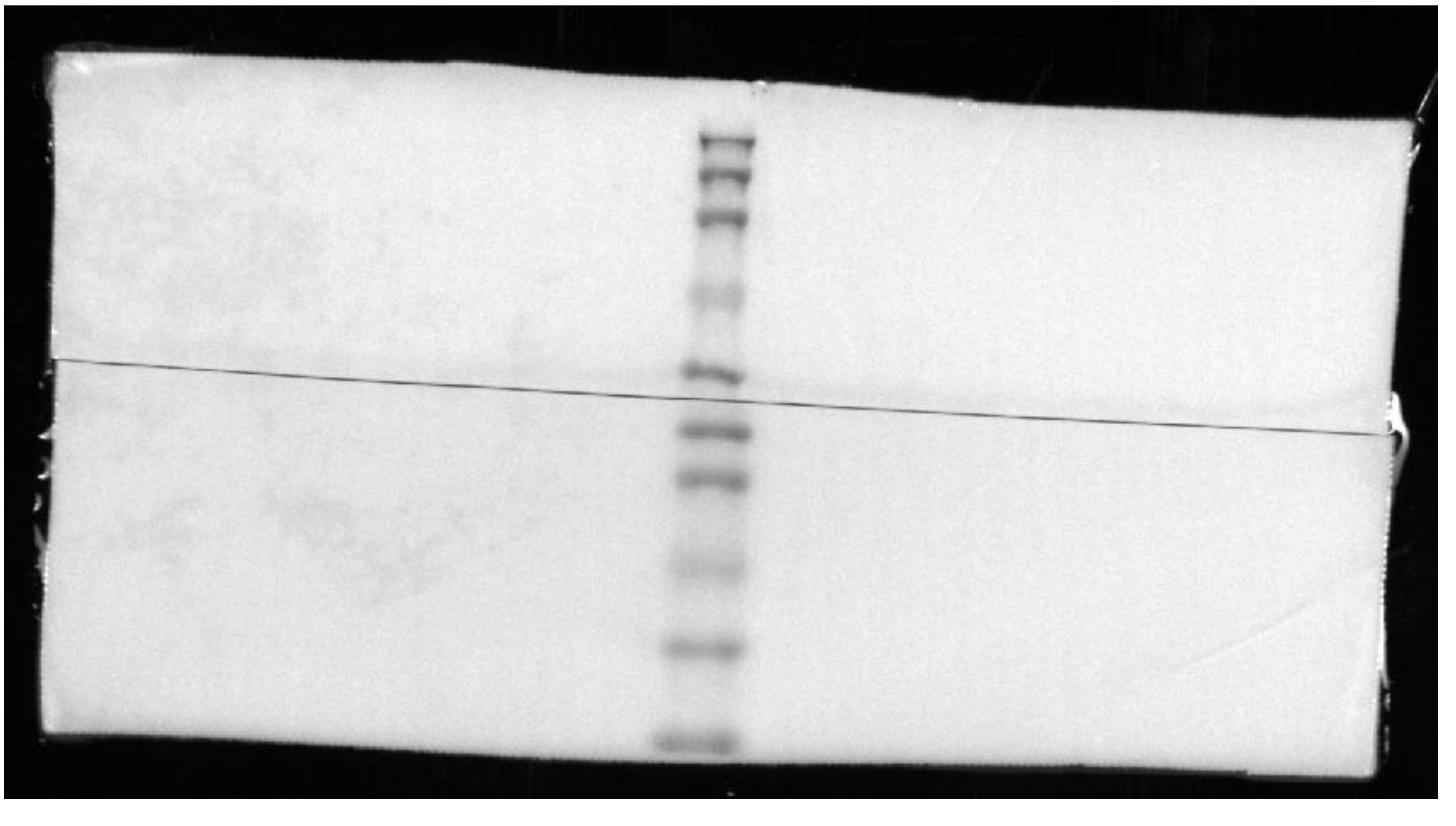

Figure 6L： TFCP2; GAPDH

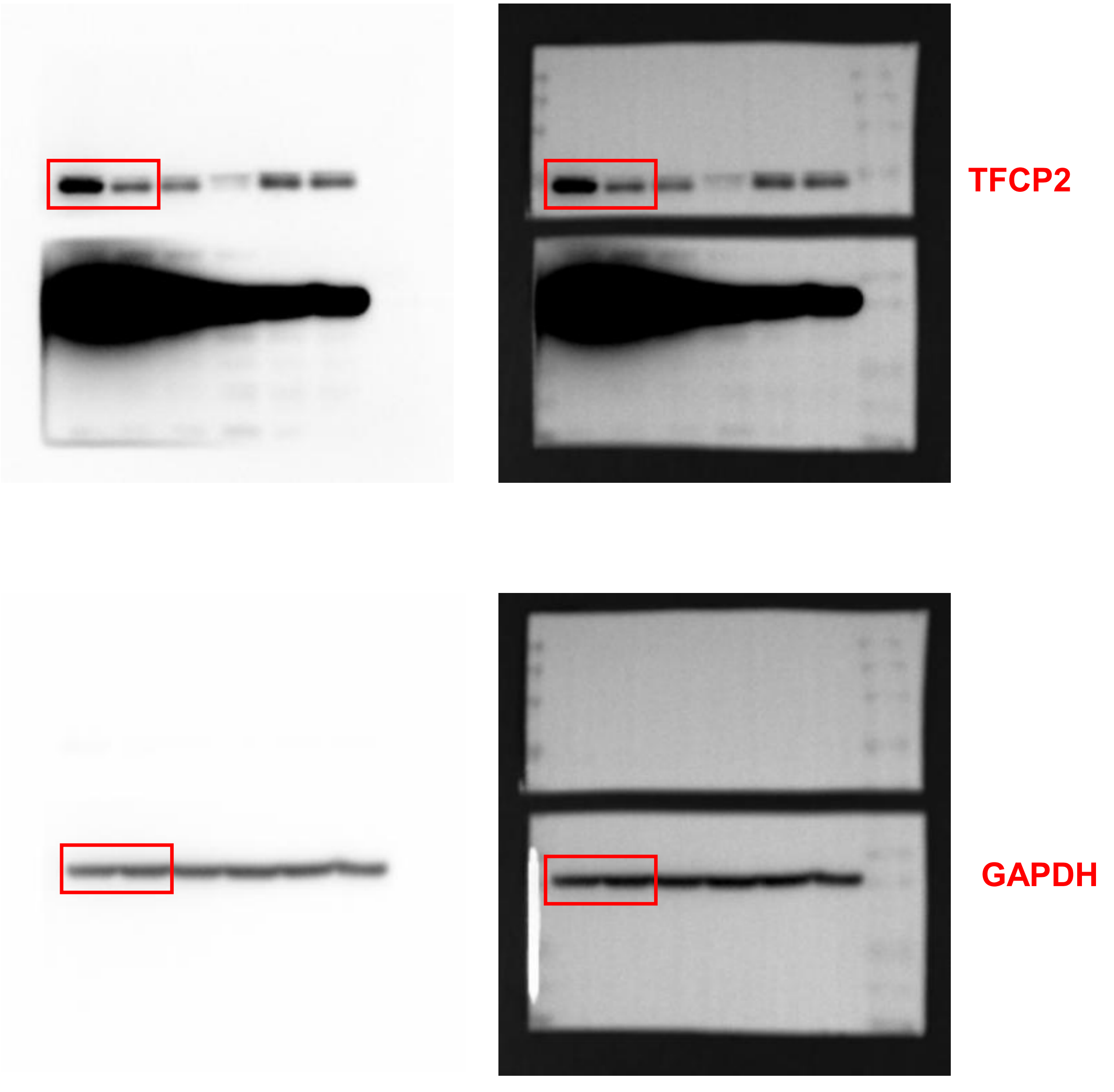

Figure 7B: KLF2; GAPDH

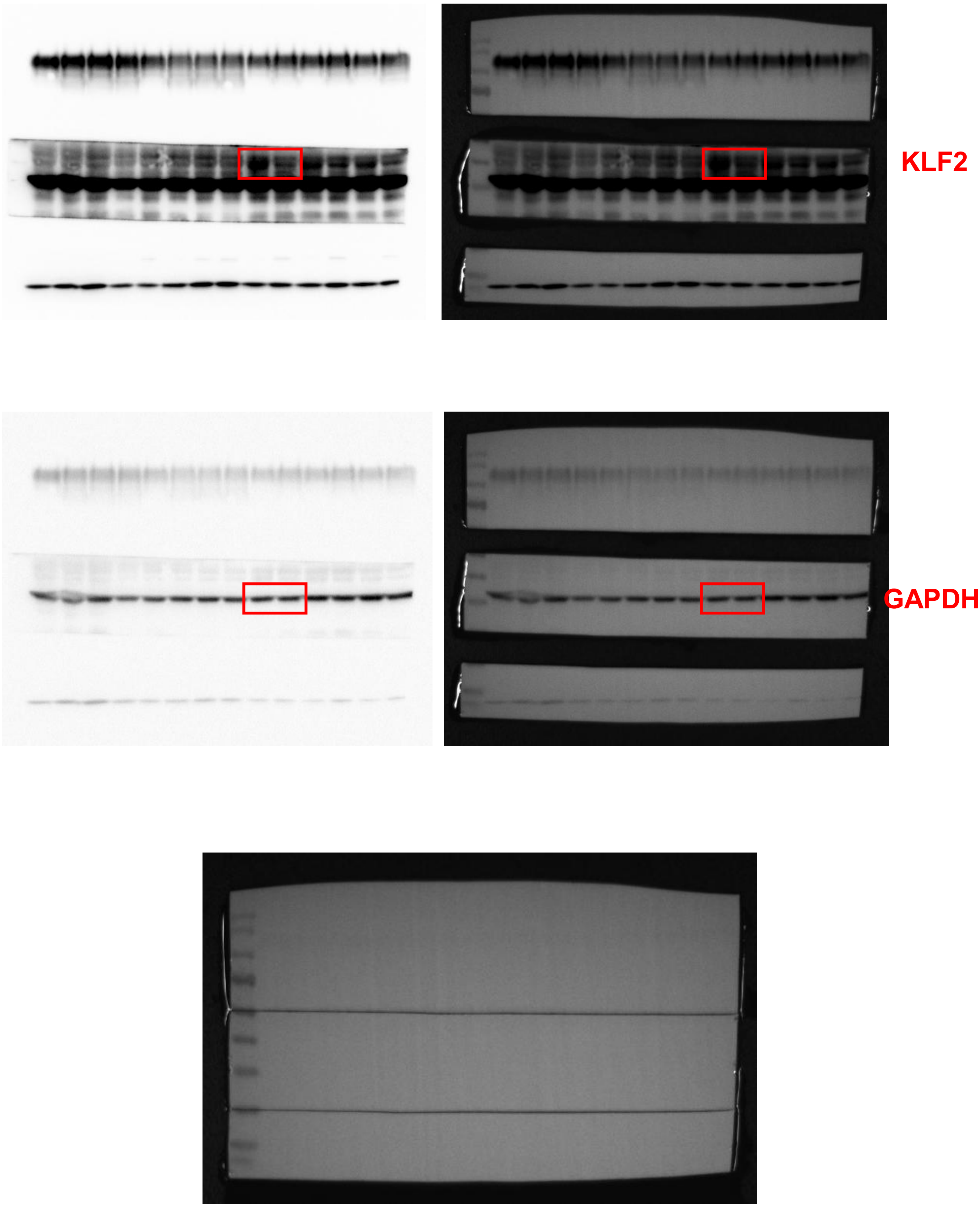

Figure S1B：SLC7A11; β-actin

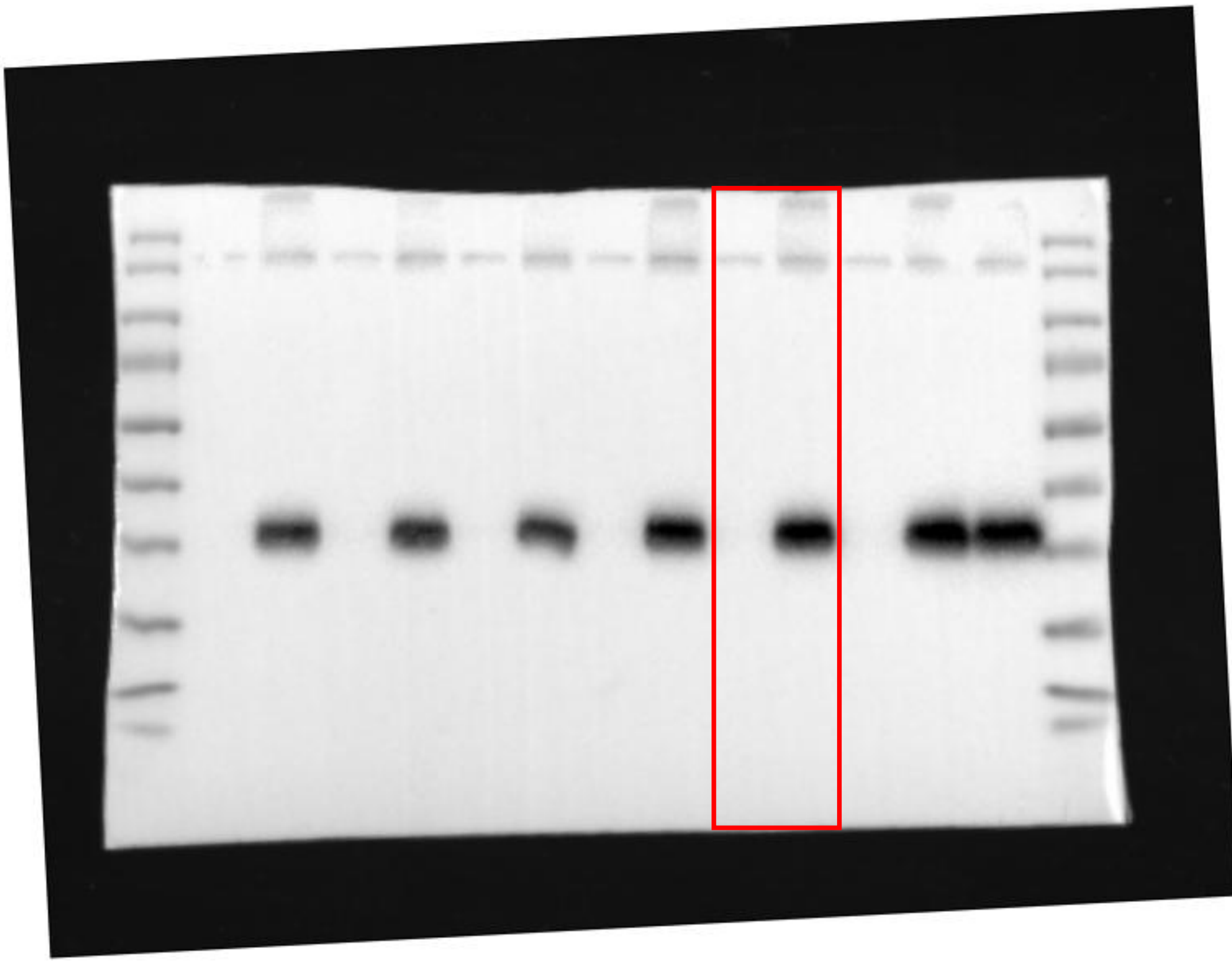

SLC7A11

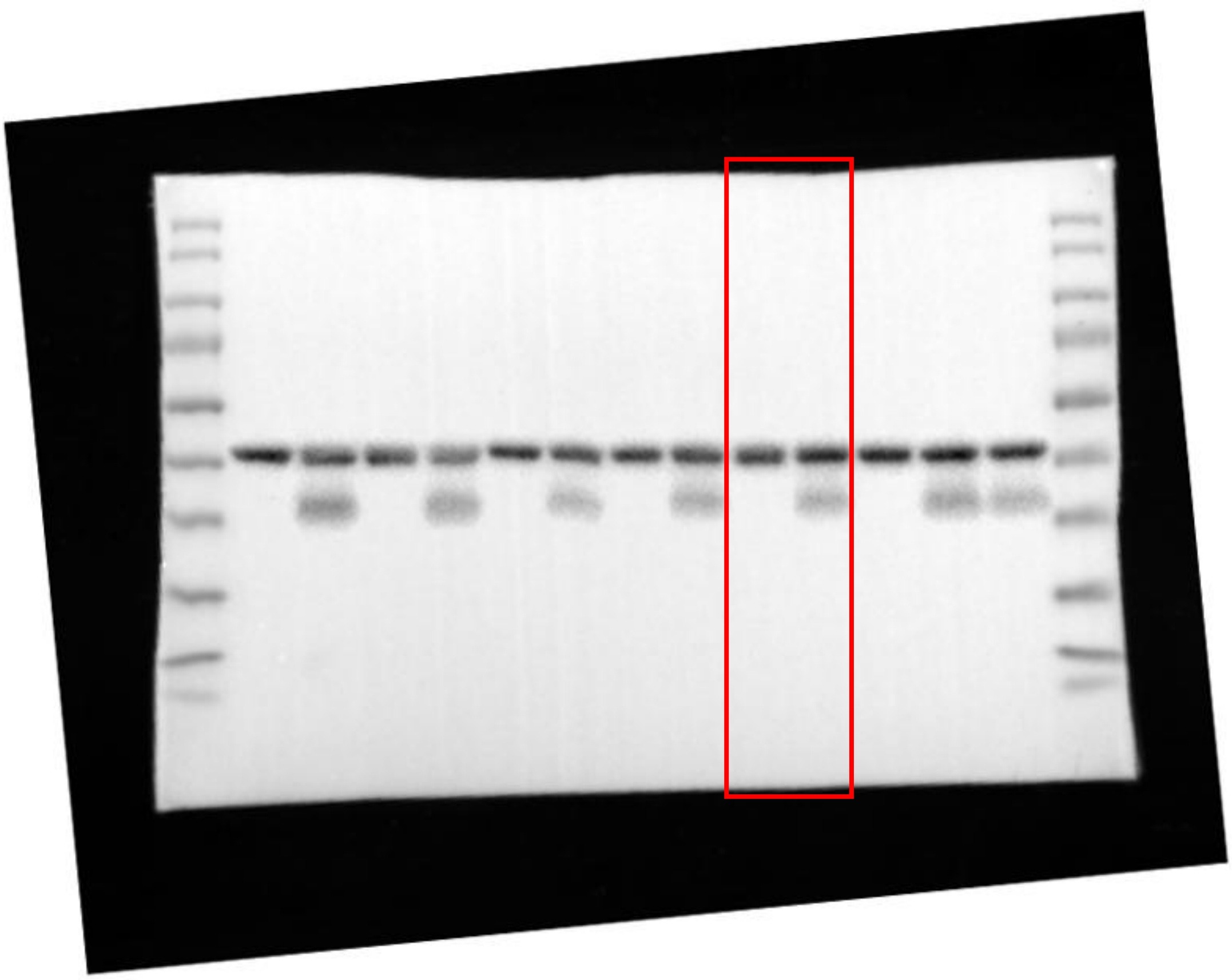

β-actin

Figure S2A : SLC7A11

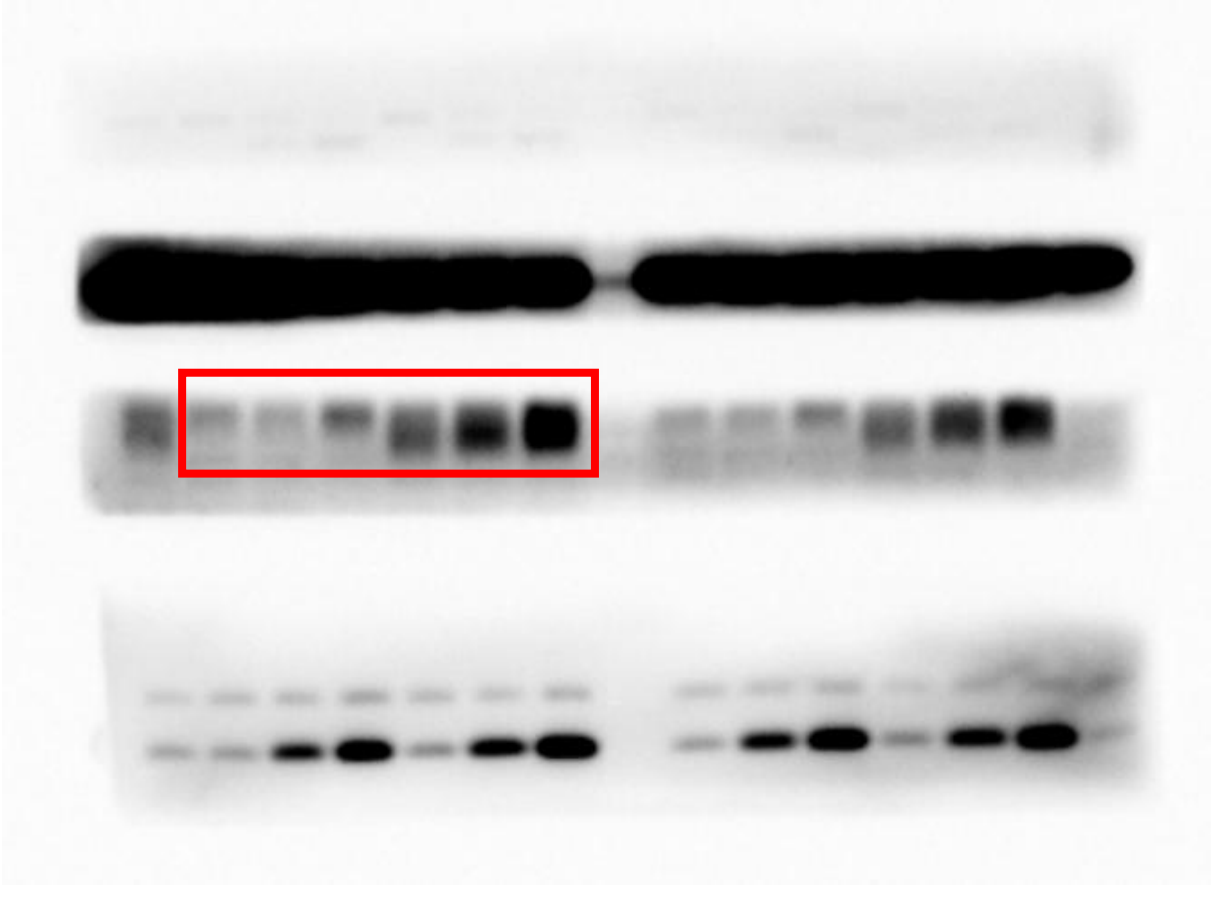

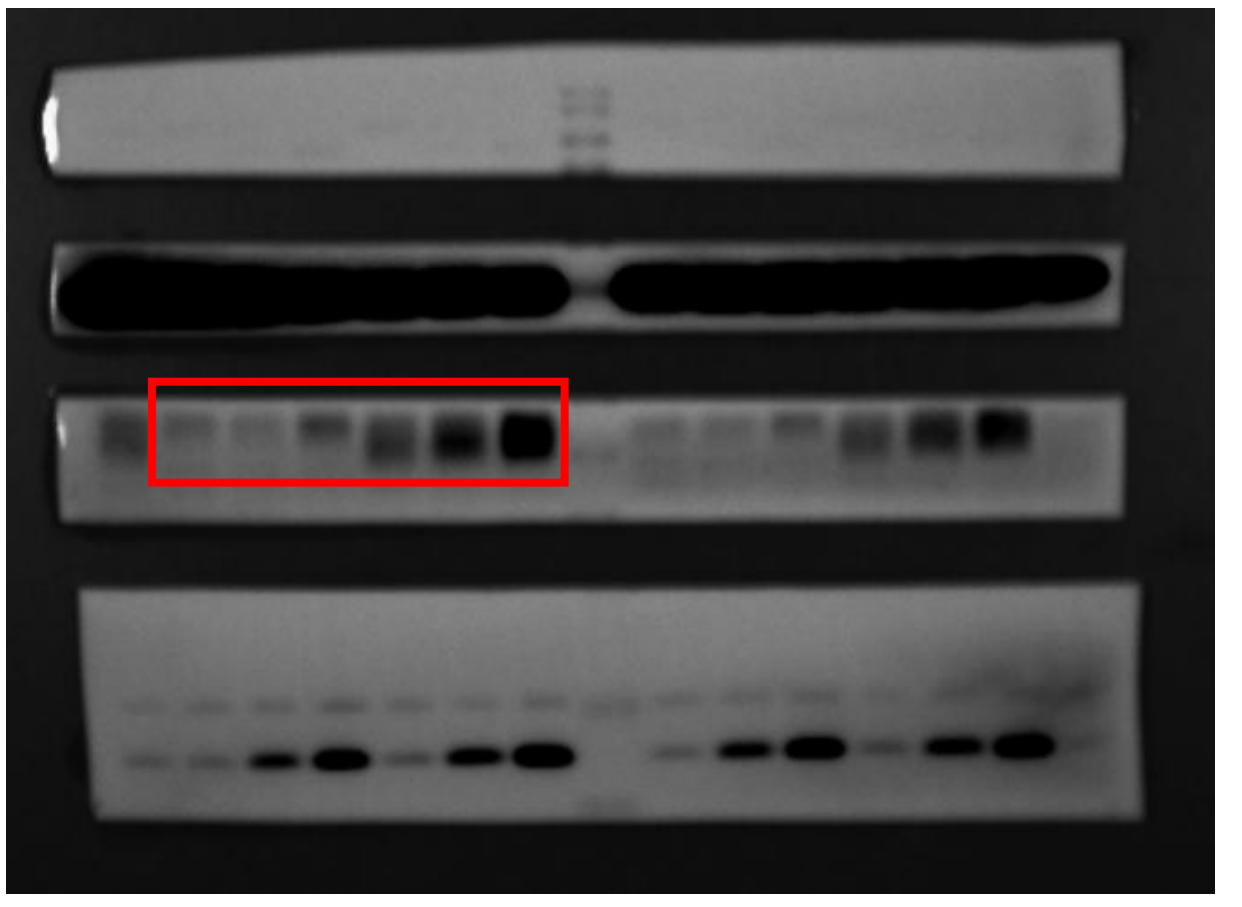

SLC7A11

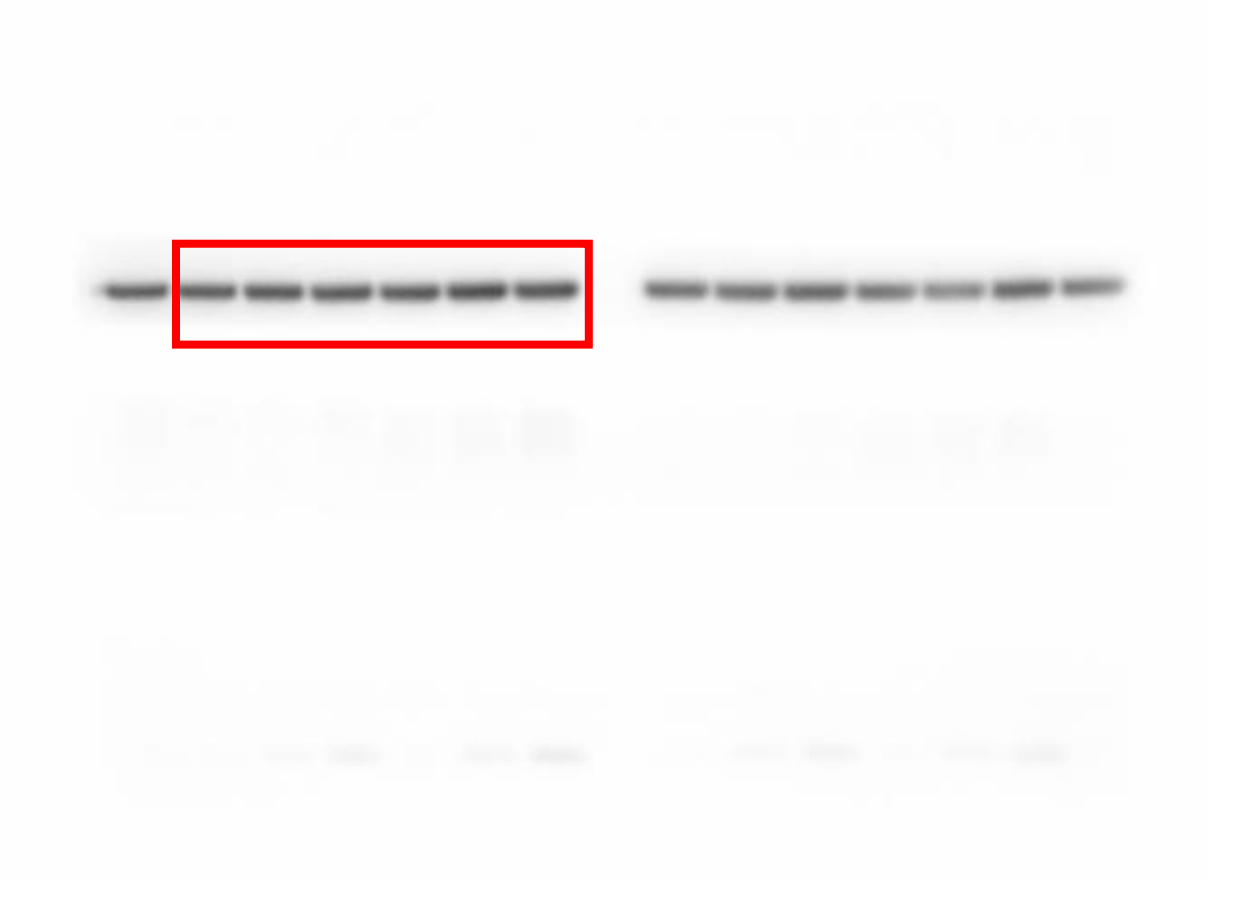

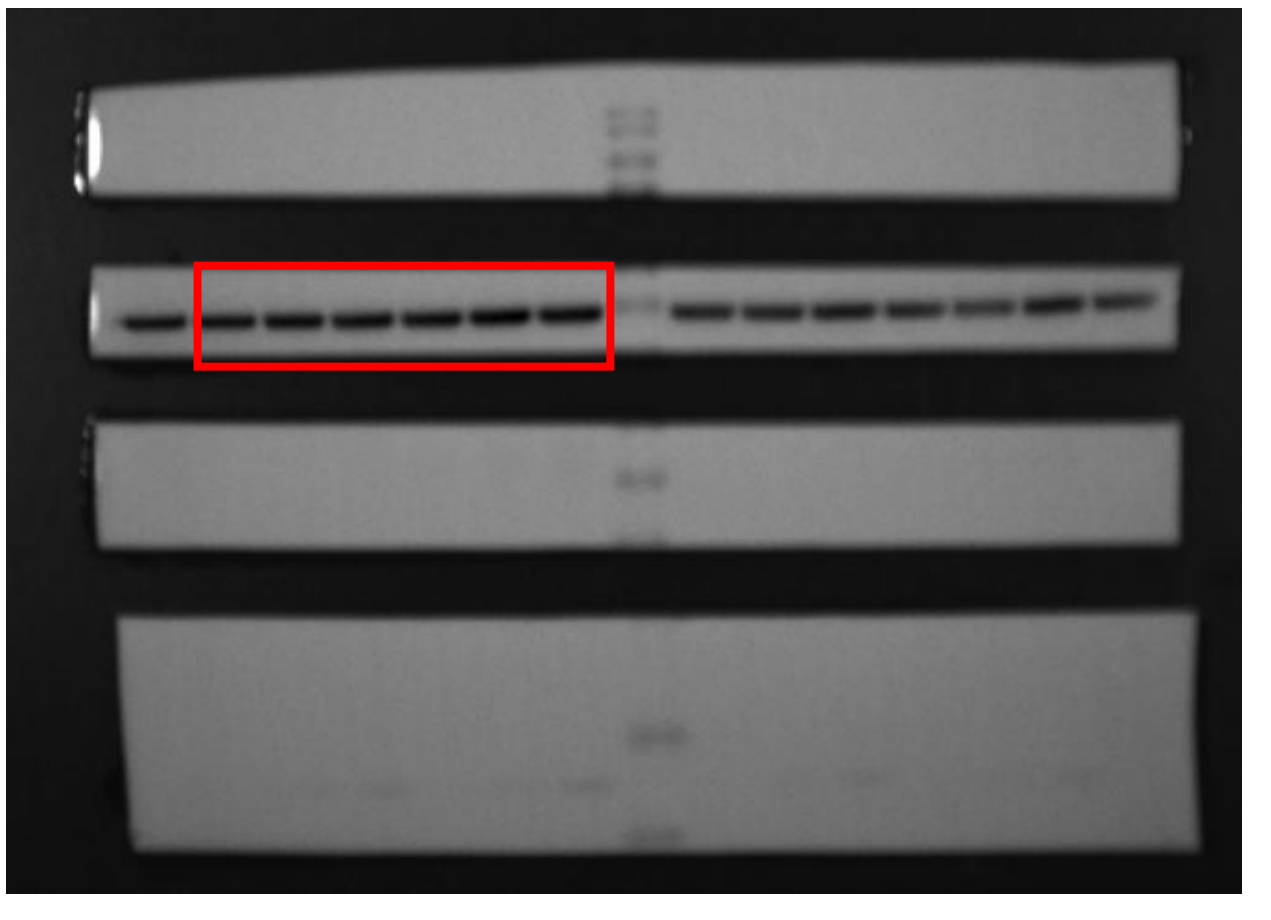

β-Tubulin

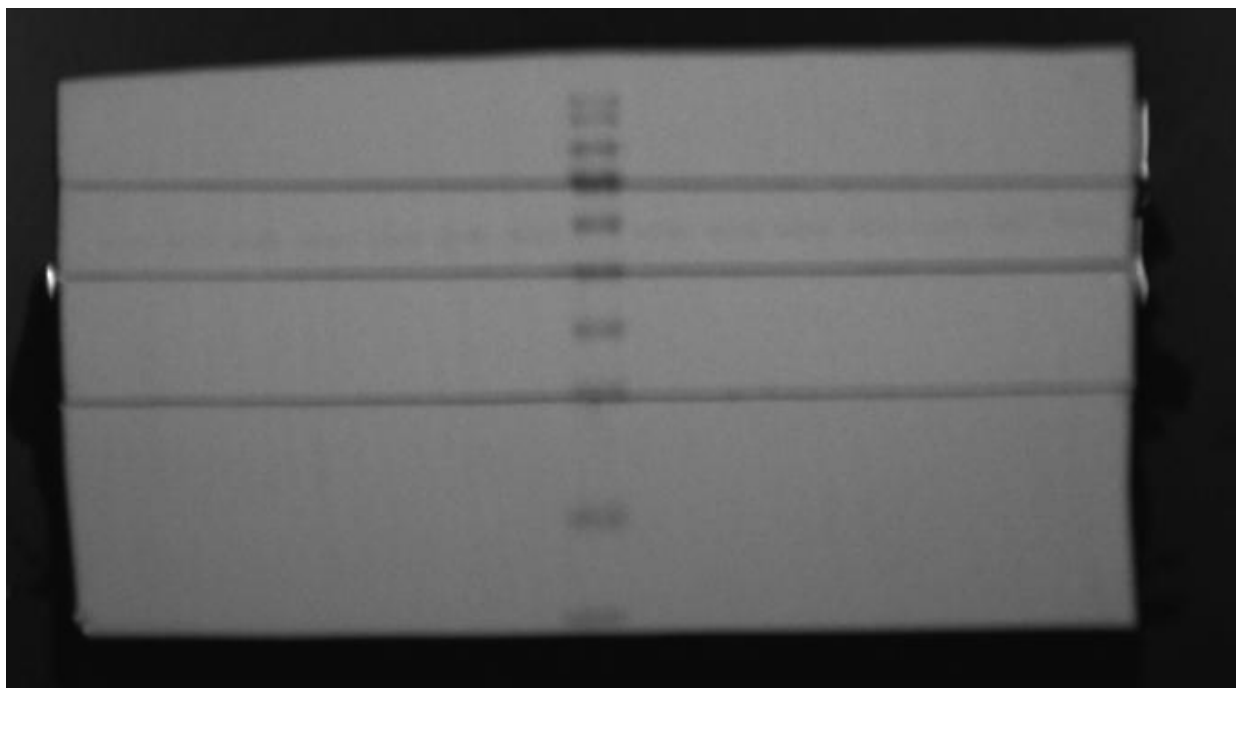

Figure S2A : p21 Waf1/Cip1; GAPDH

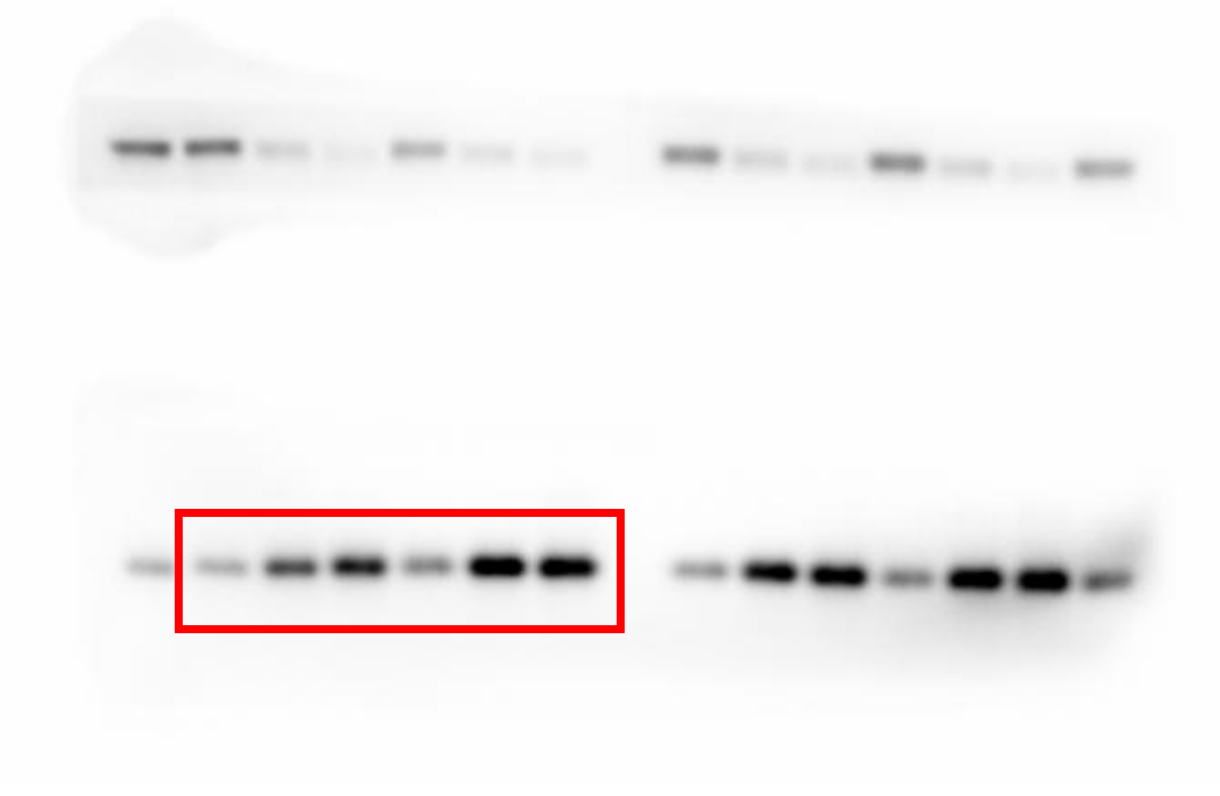

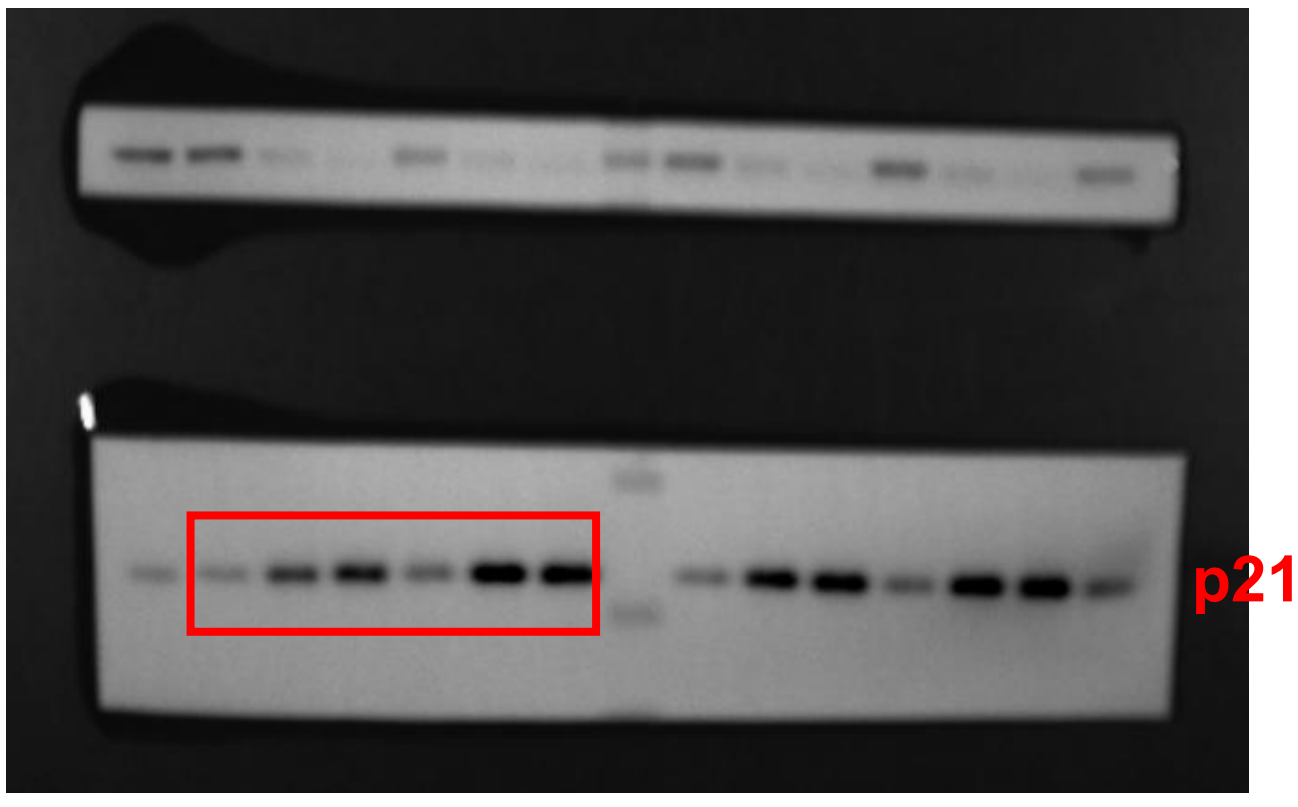

p21 Waf1/Cip1

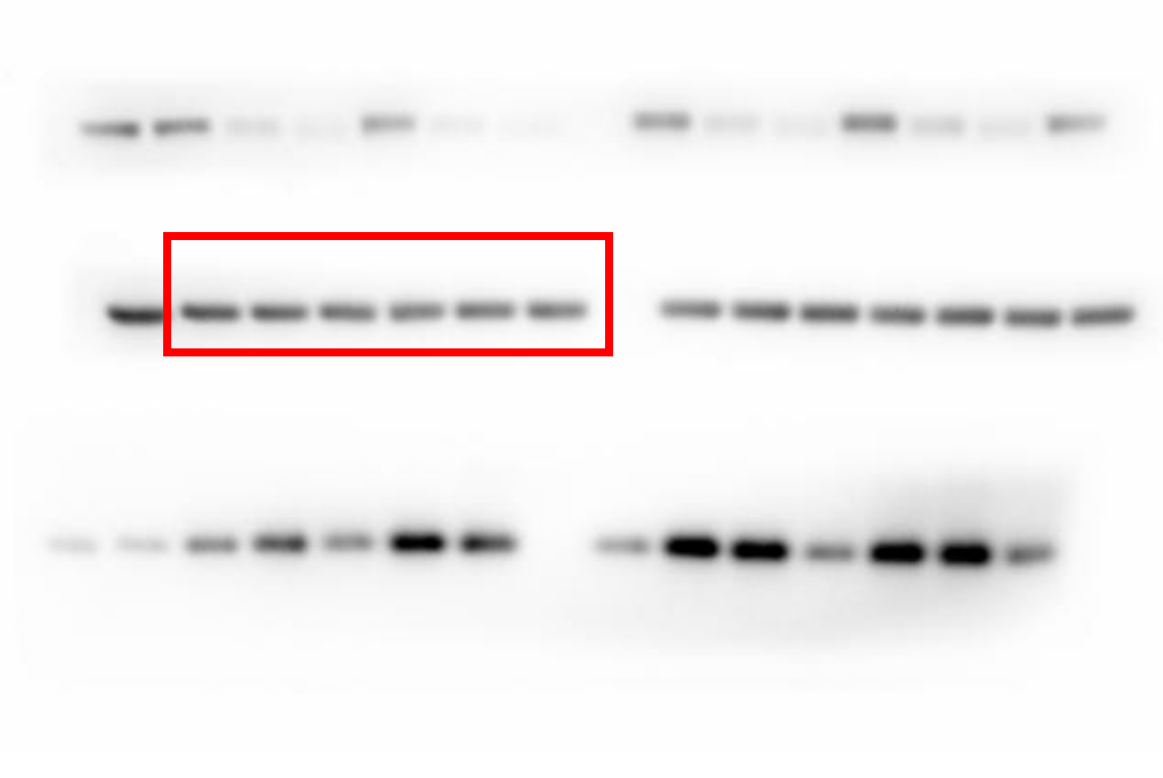

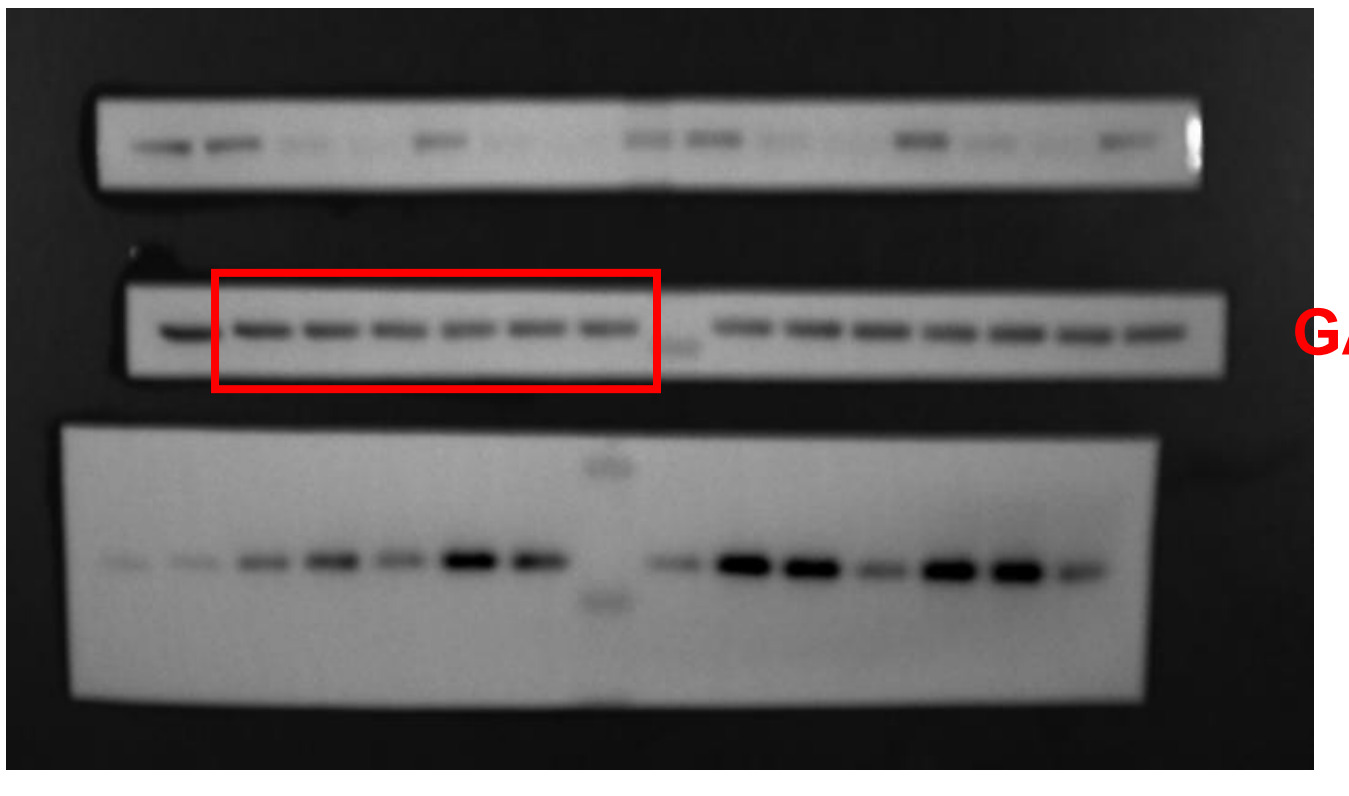

GAPDH

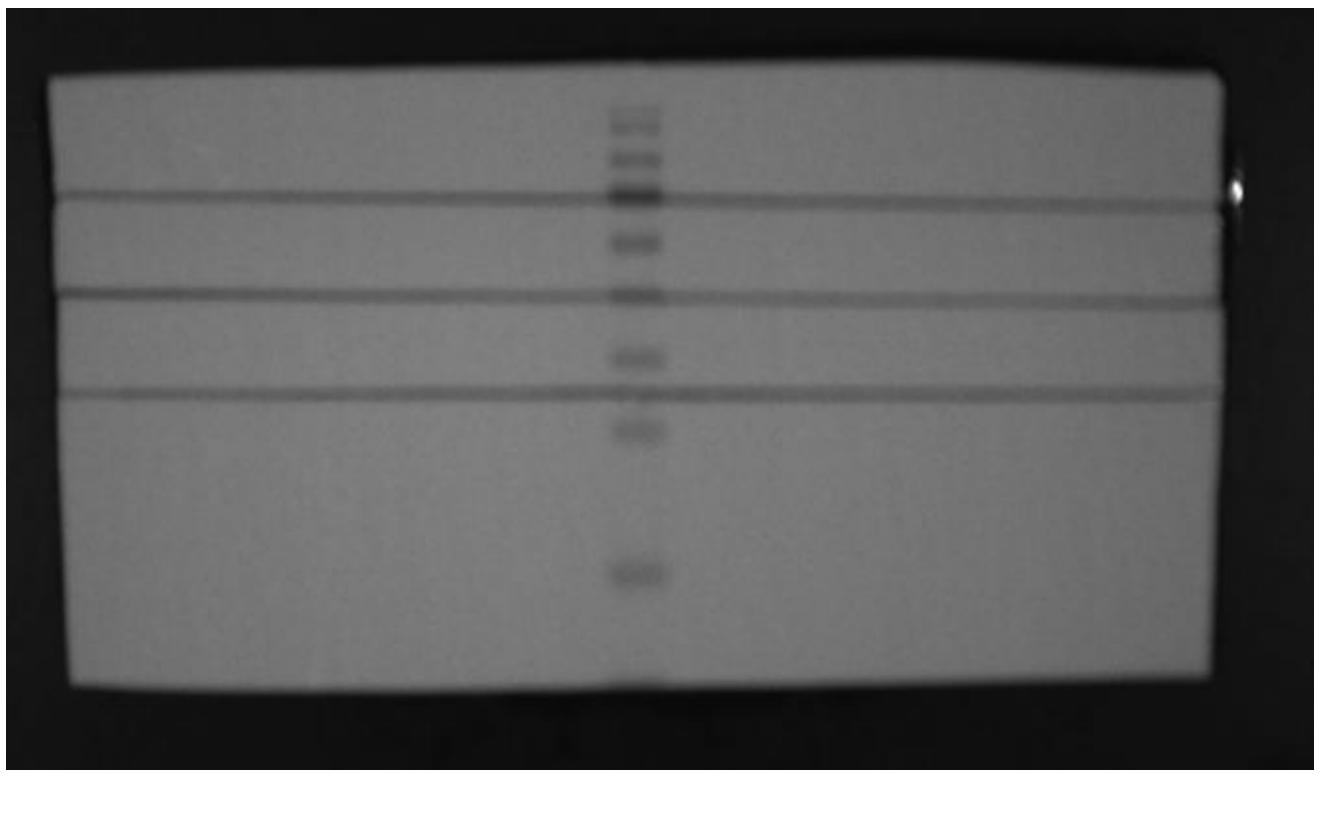

Figure S2A : p27 Kip1

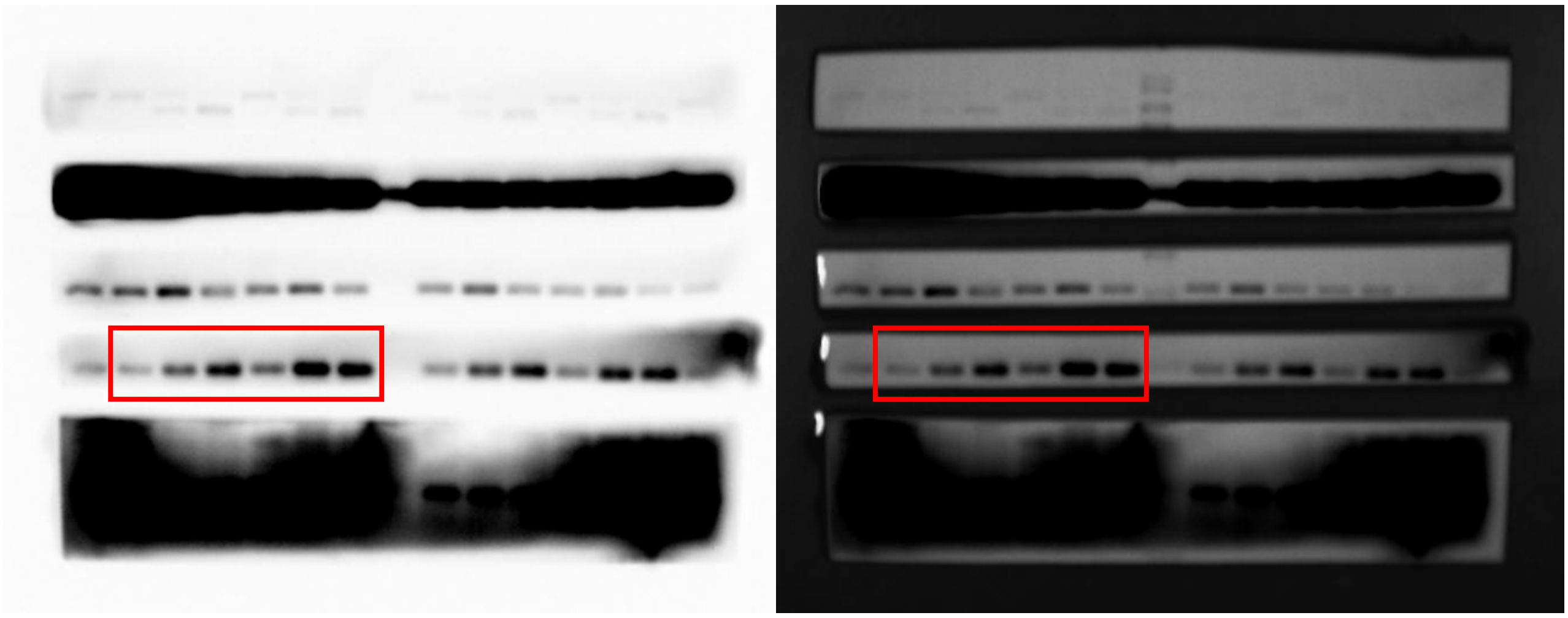

p27 Kip1

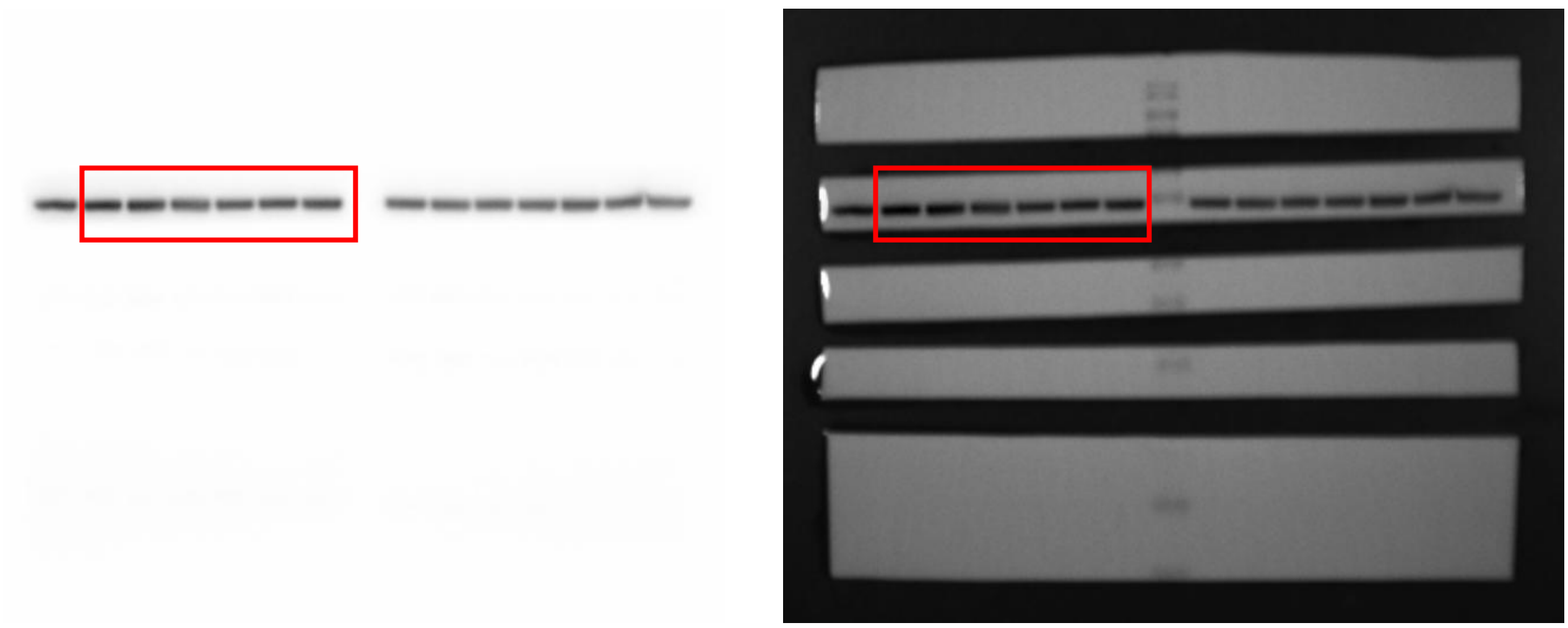

β-Tubulin

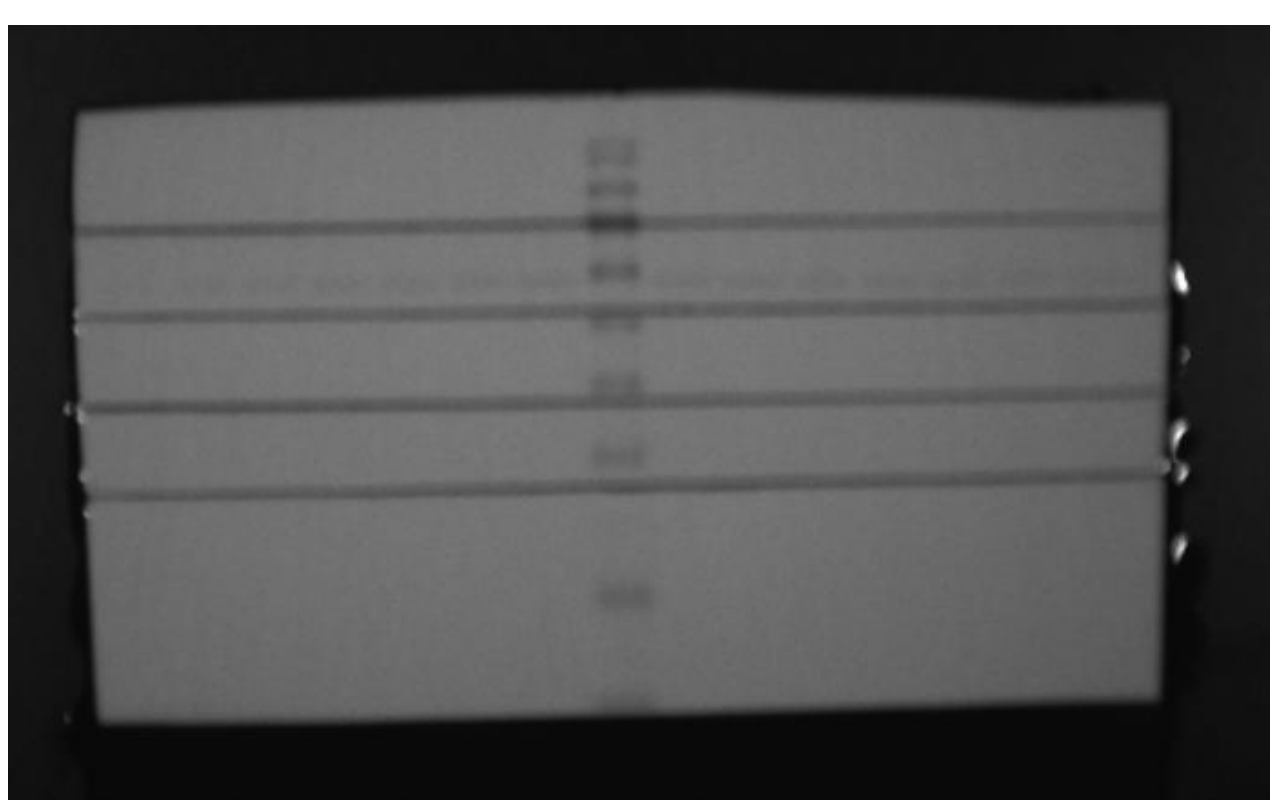

Figure S2A : p18 INK4C; β-Tubulin

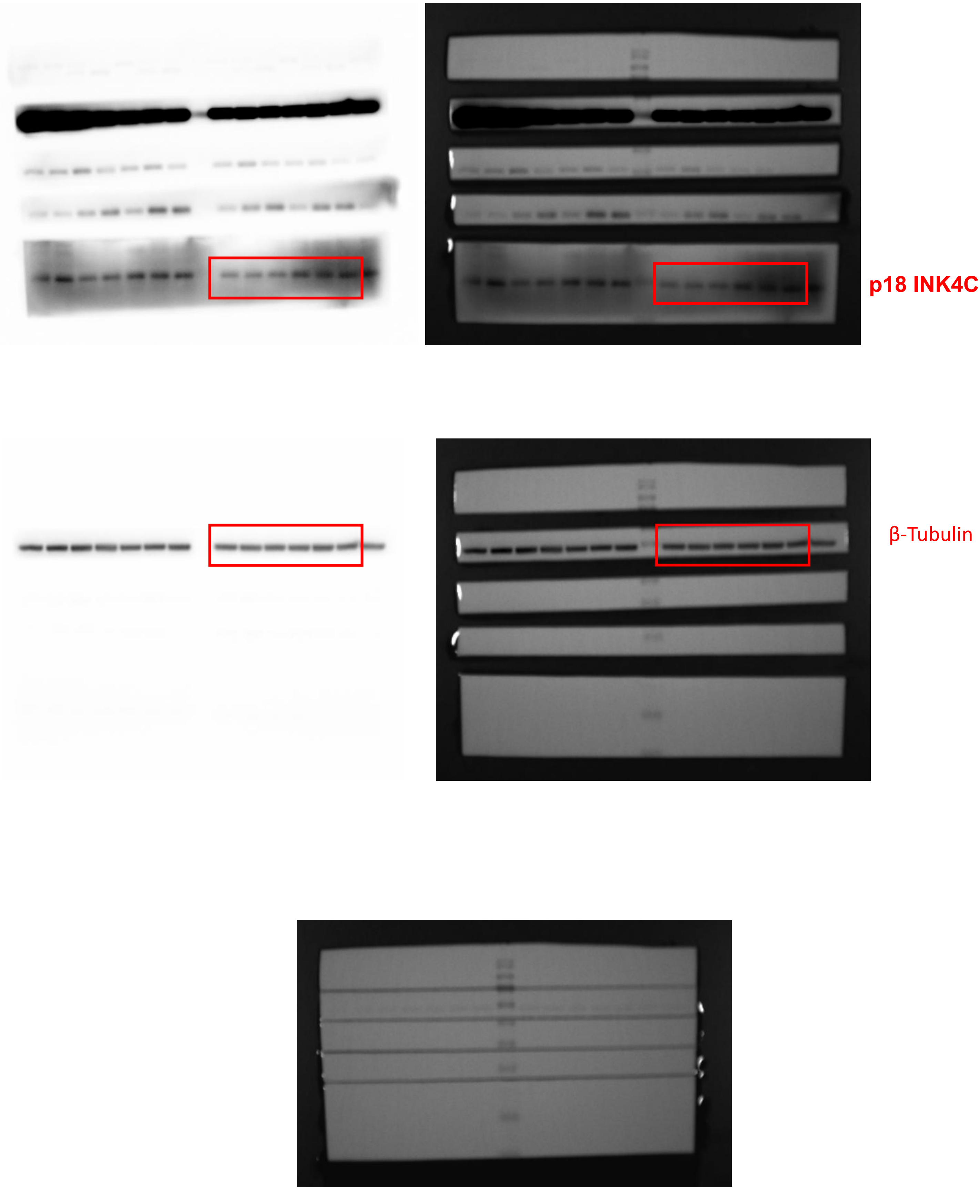

Figure S2A : Cyclin B1

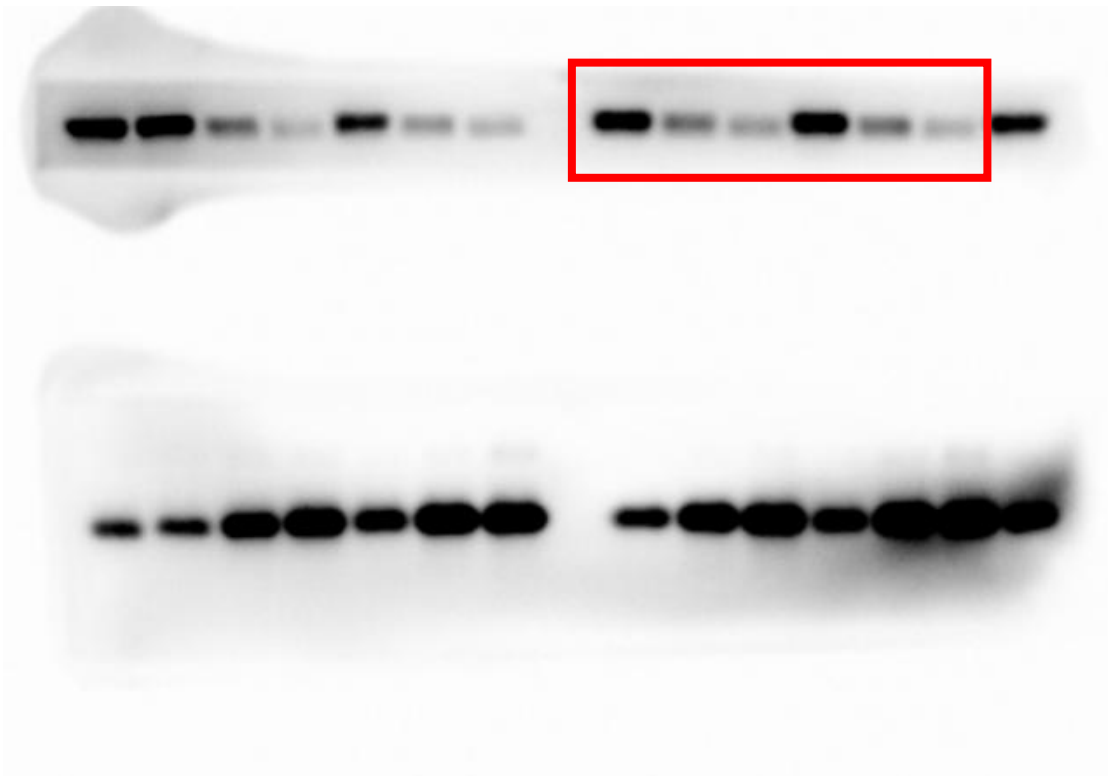

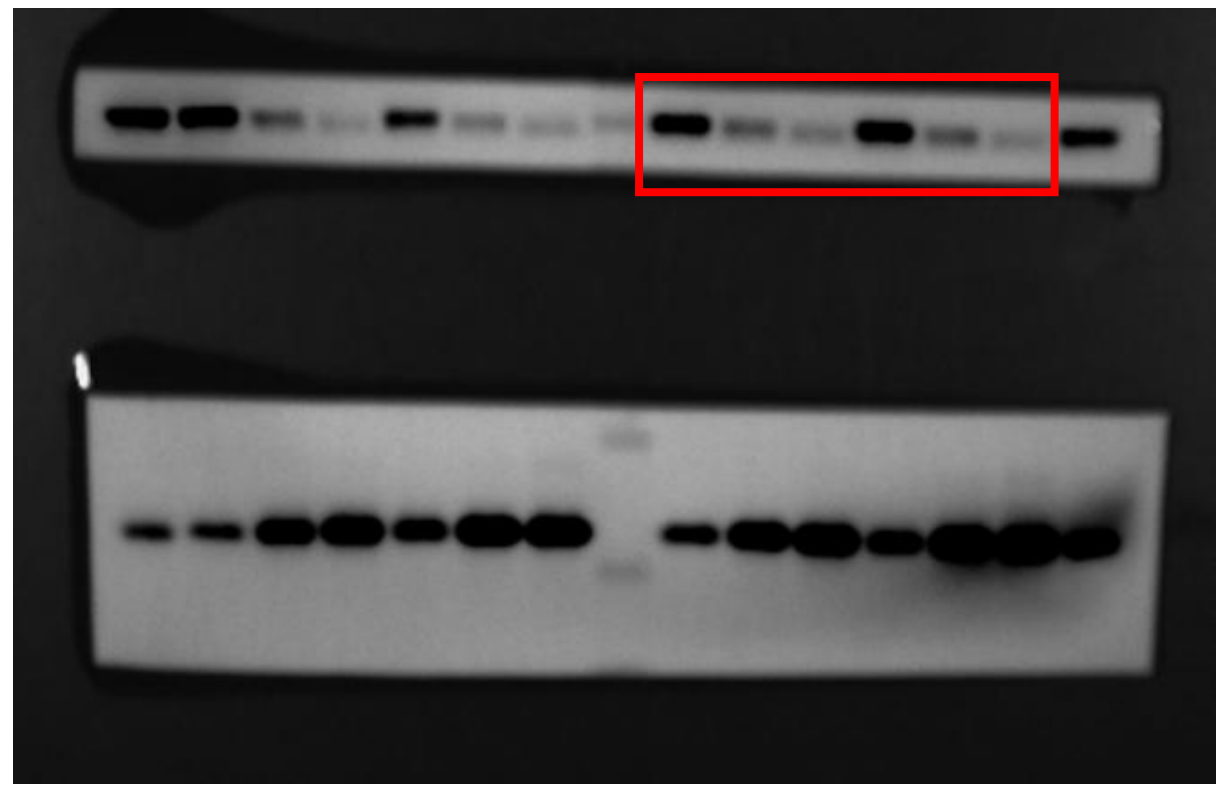

Cyclin B1

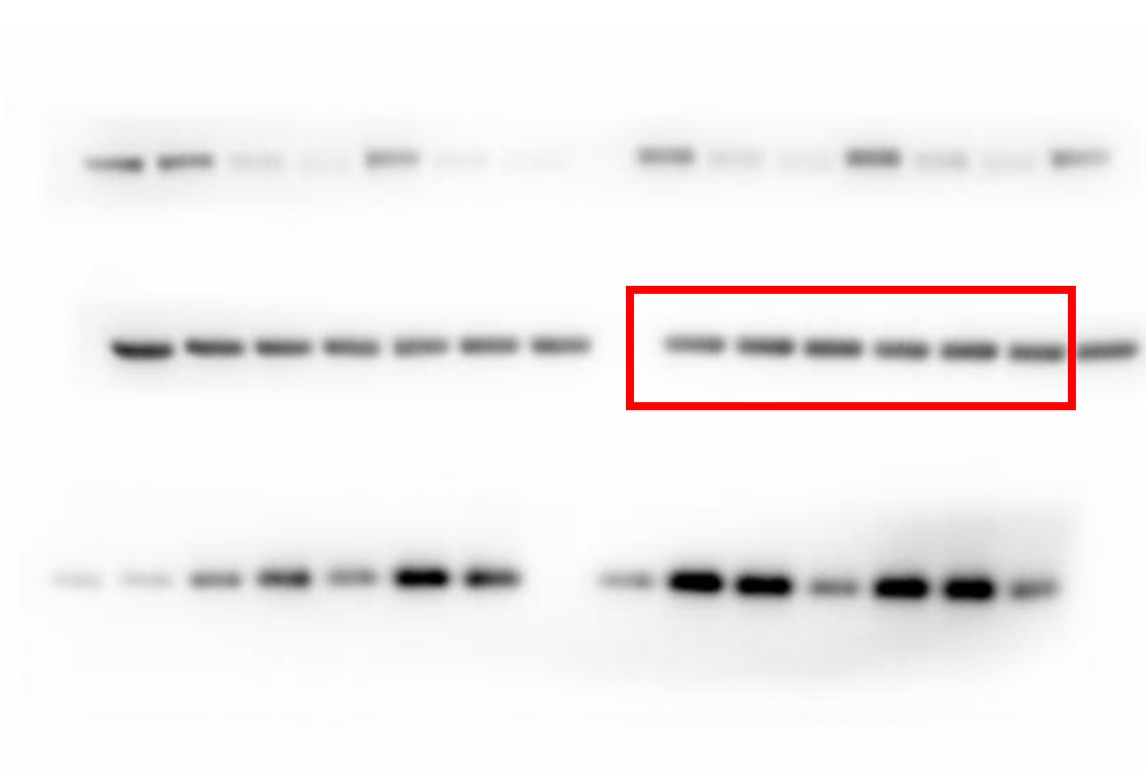

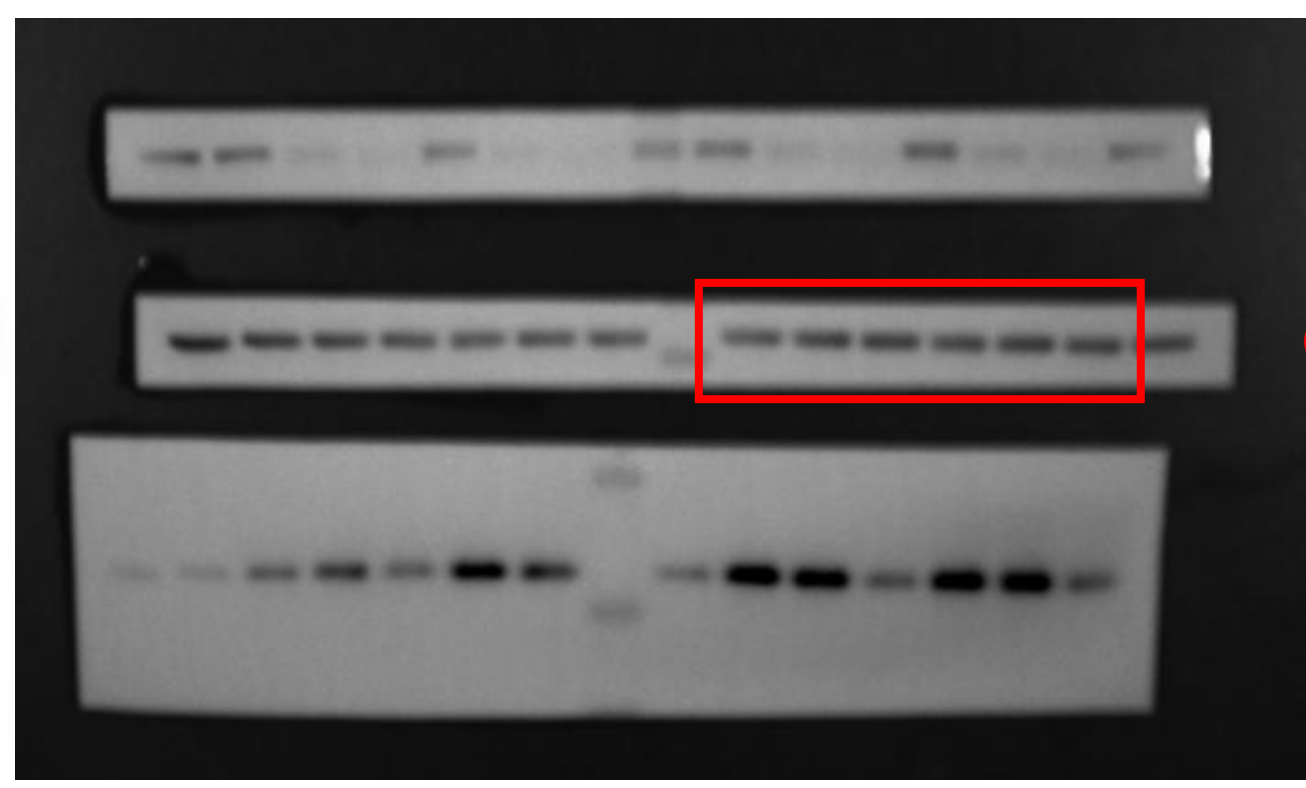

GAPDH

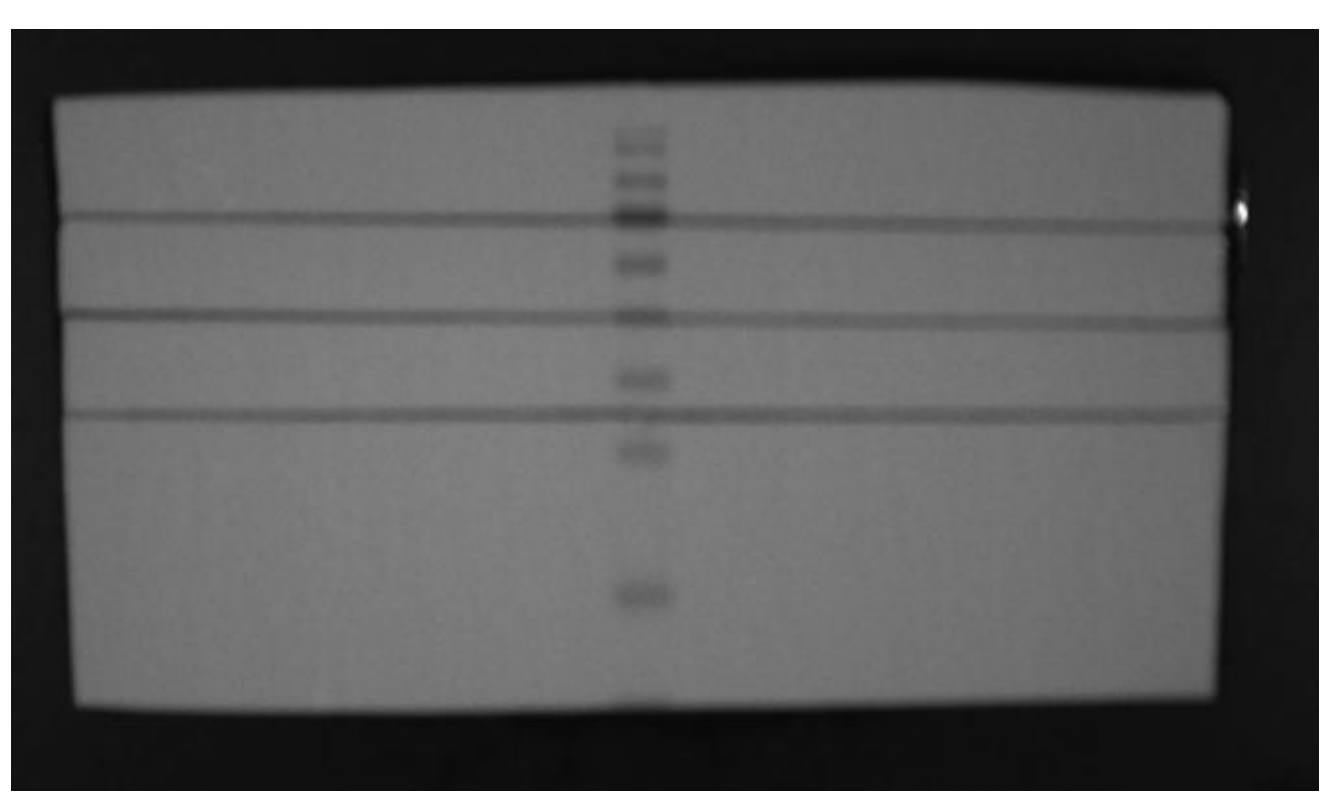

Figure S2A : Cyclin D1

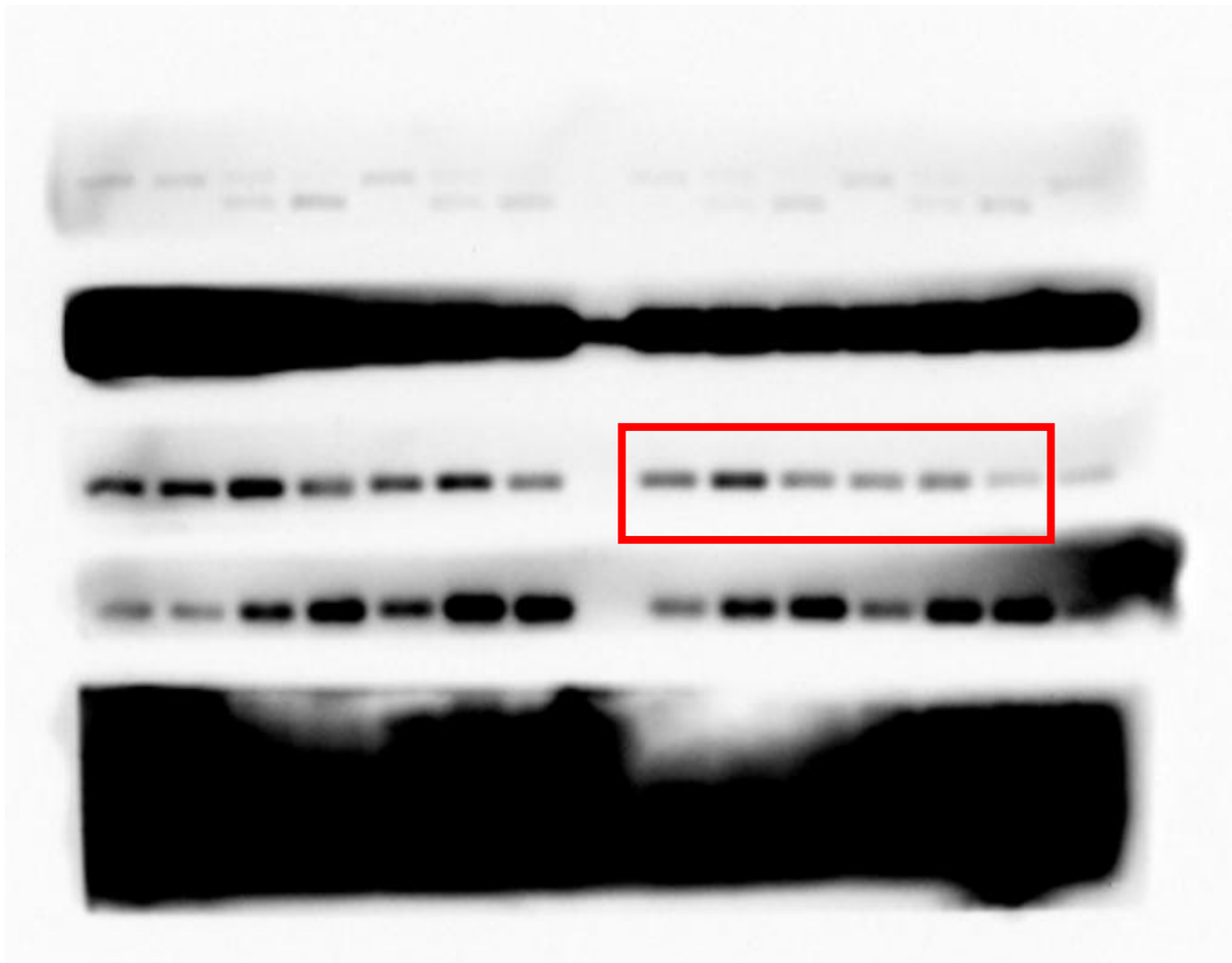

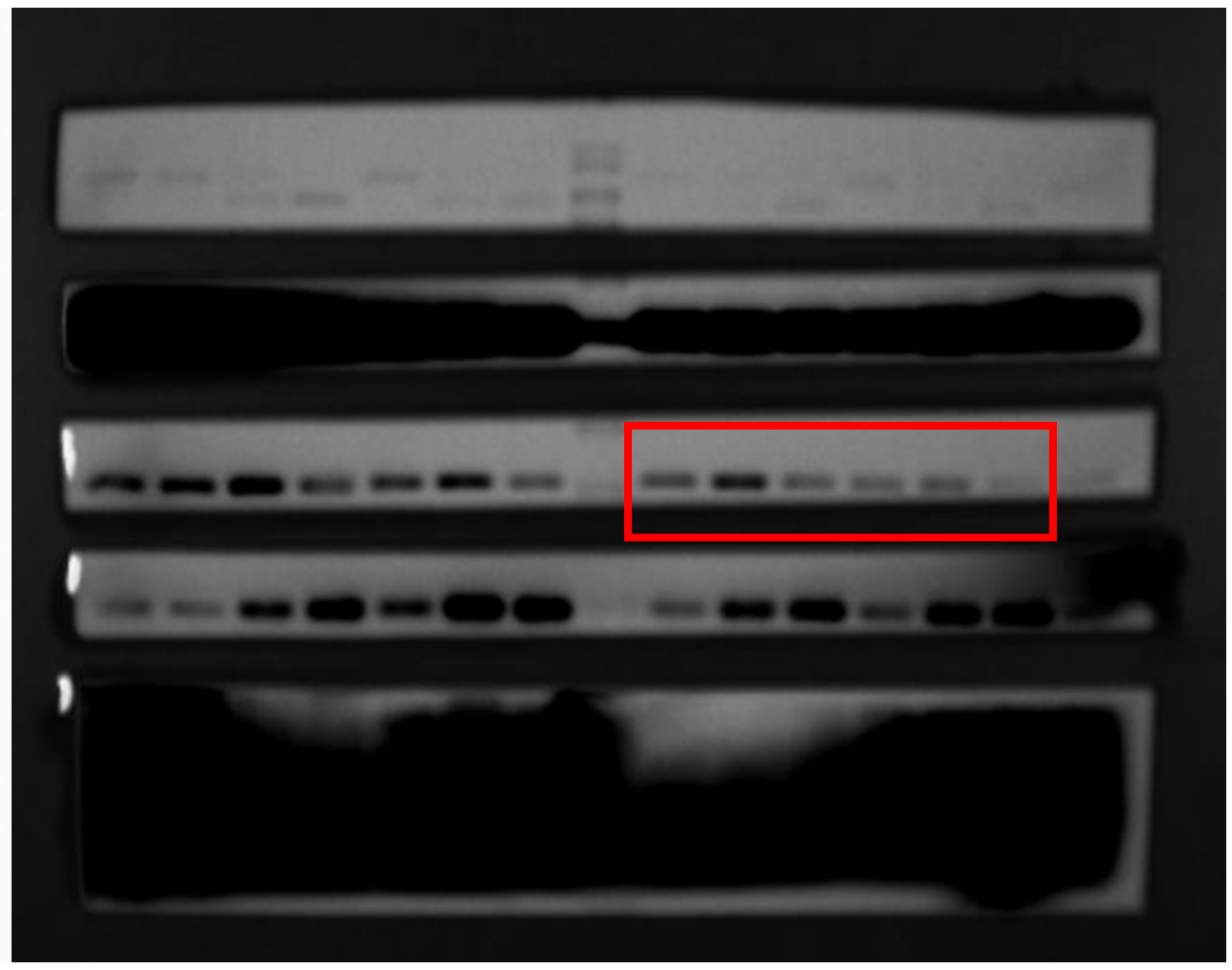

Cyclin D1

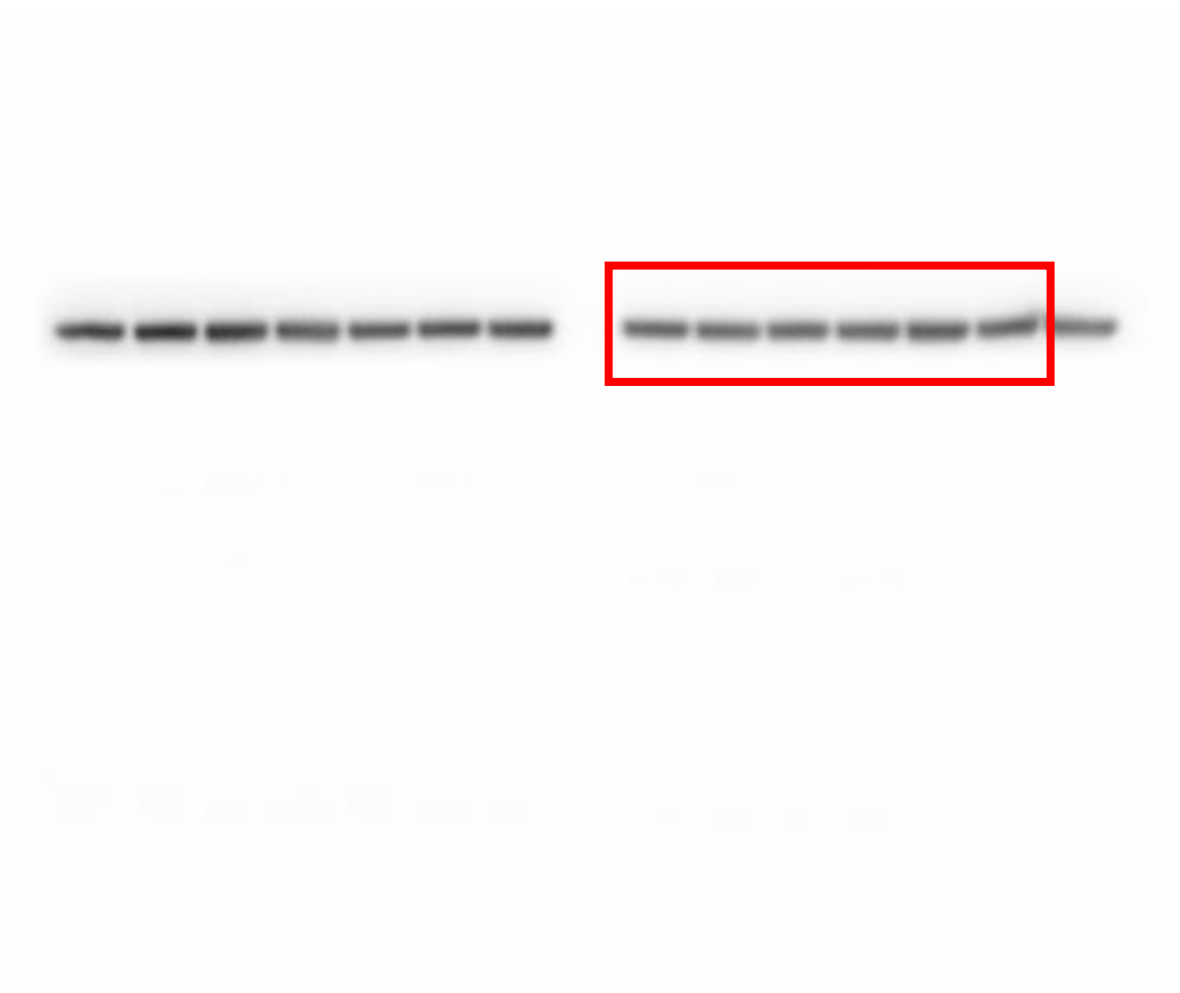

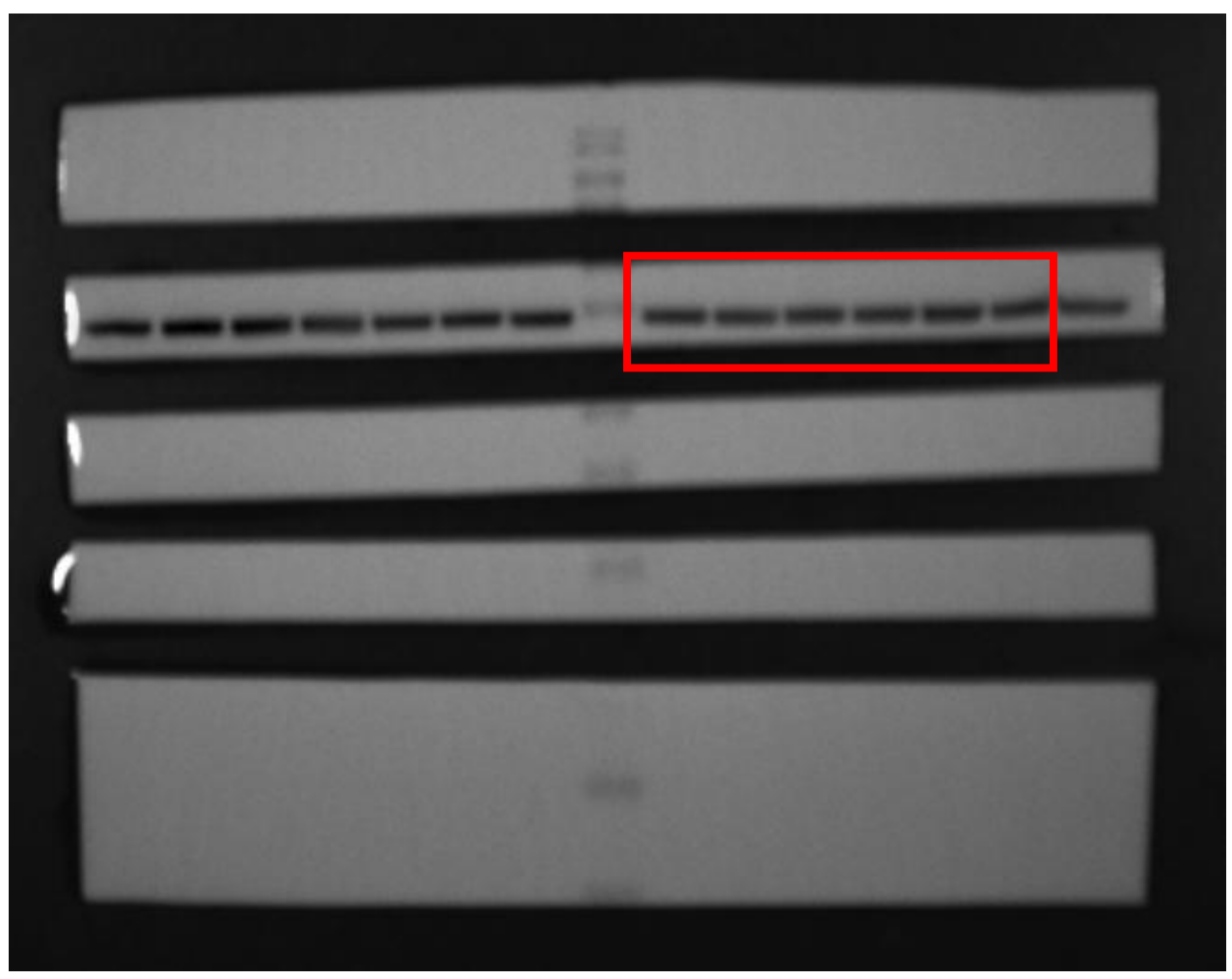

β-Tubulin

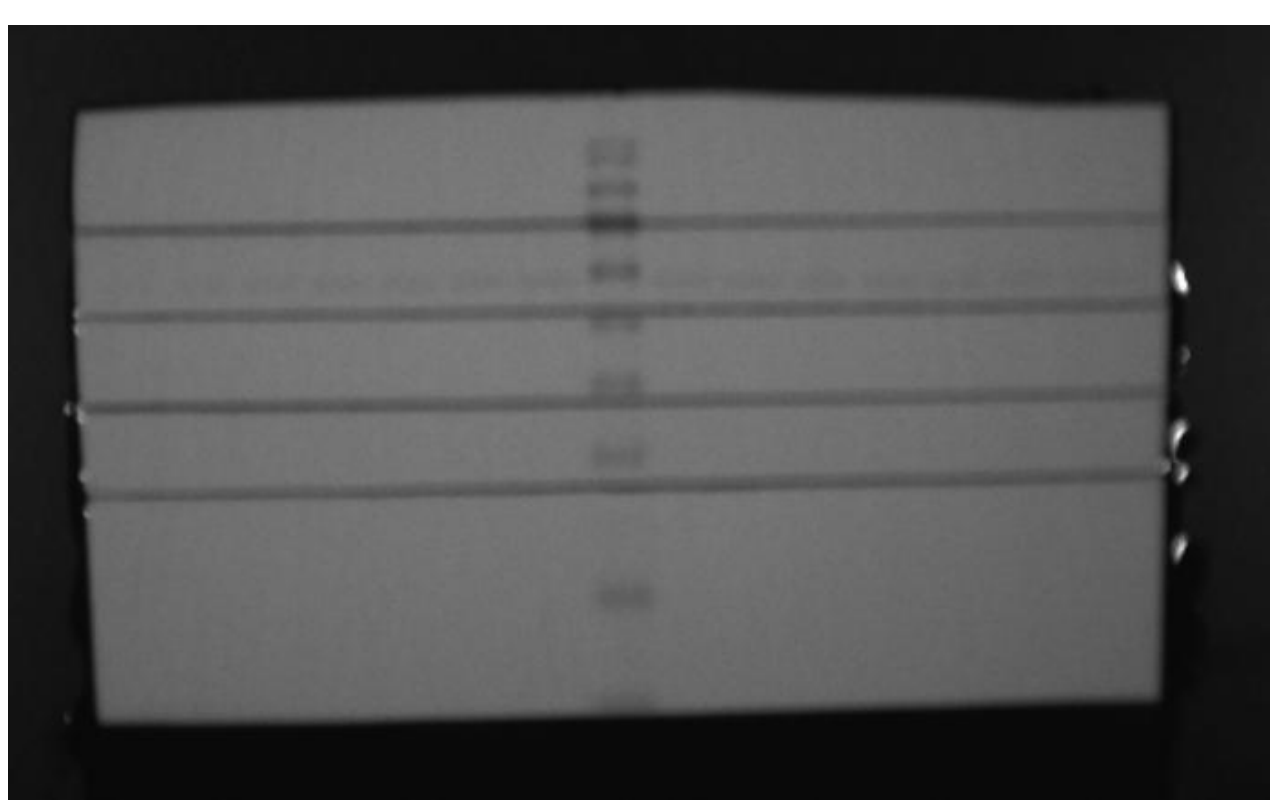

Figure S2C : Full length Lamin A/C; Cleaved Lamin A; Cleaved Lamin C、GAPDH

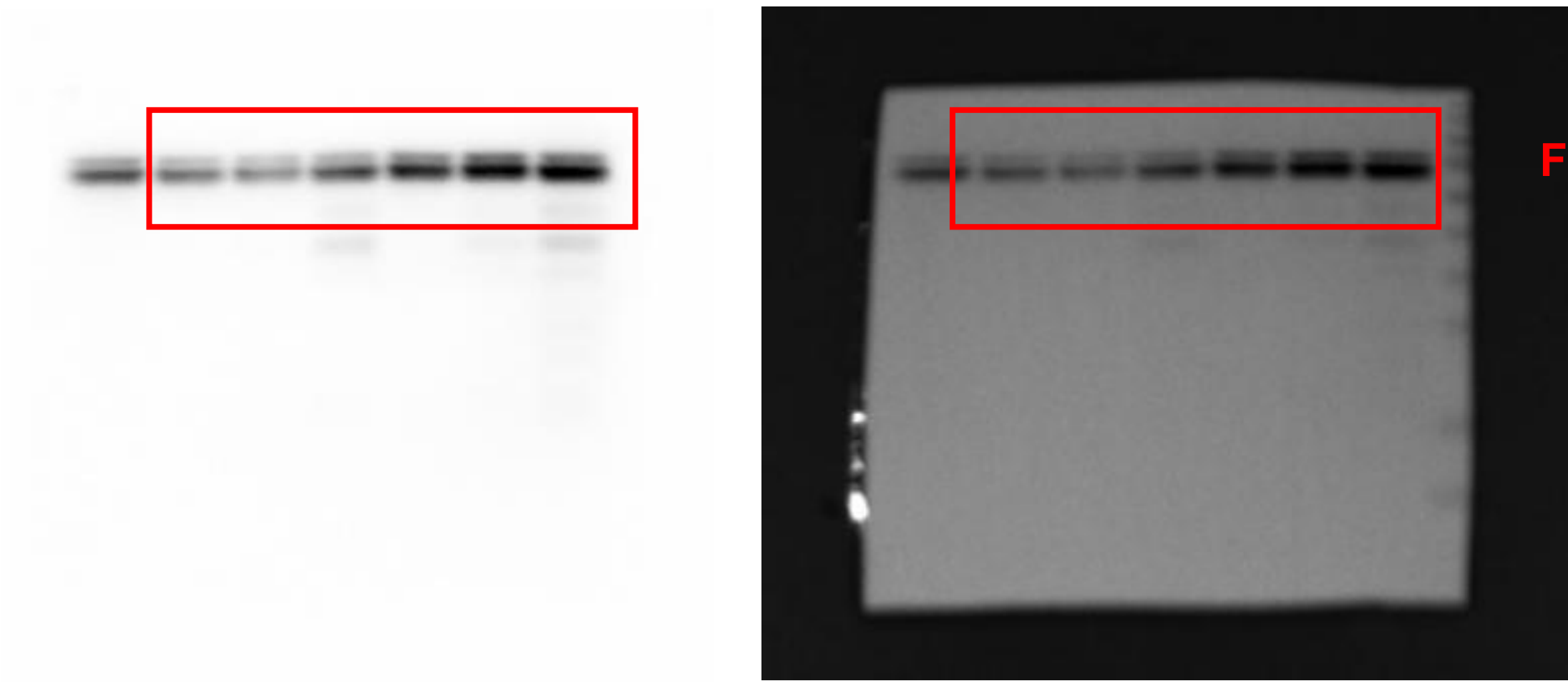

Full length Lamin A/C

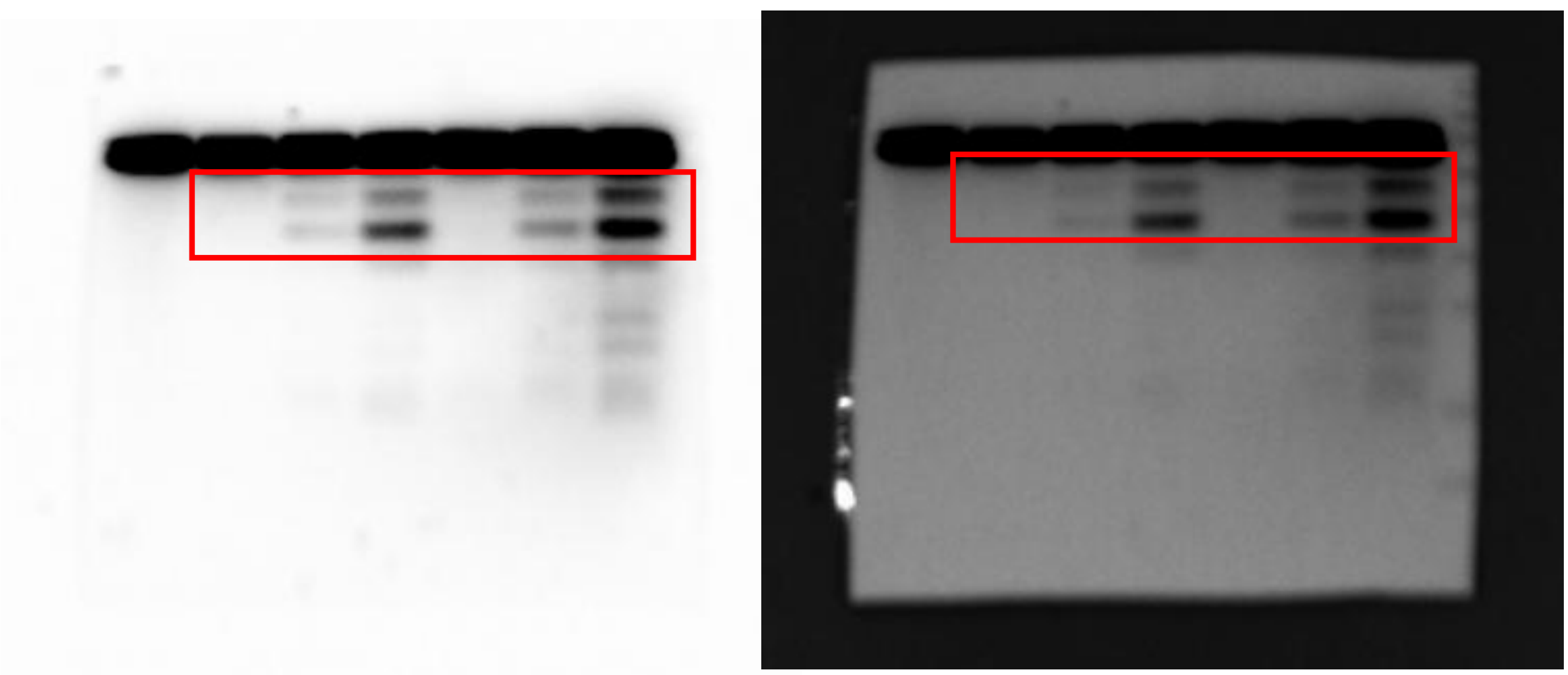

Cleaved Lamin A

Cleaved Lamin C

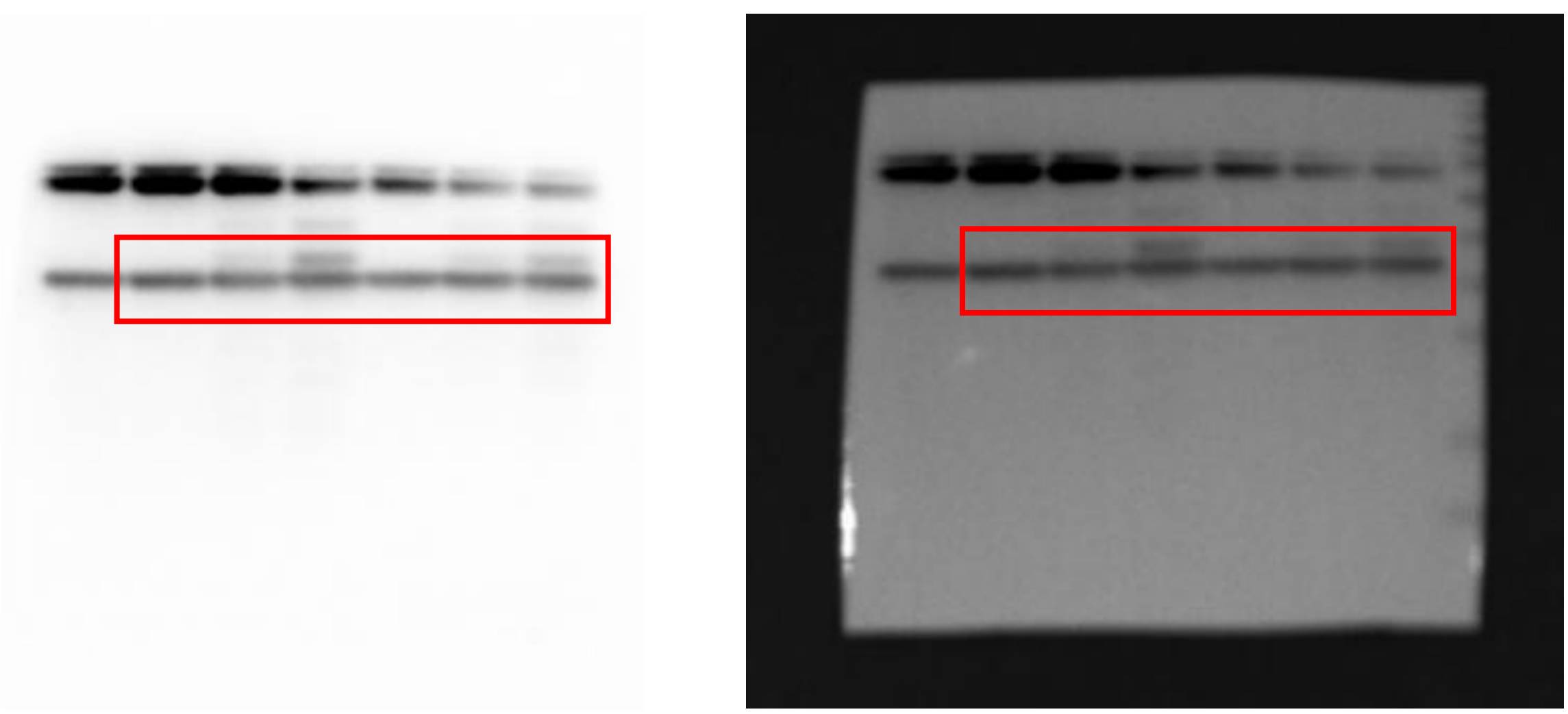

GAPDH

Figure S2C : Full length Caspase-3、Cleaved Caspase-3、β-Tubulin

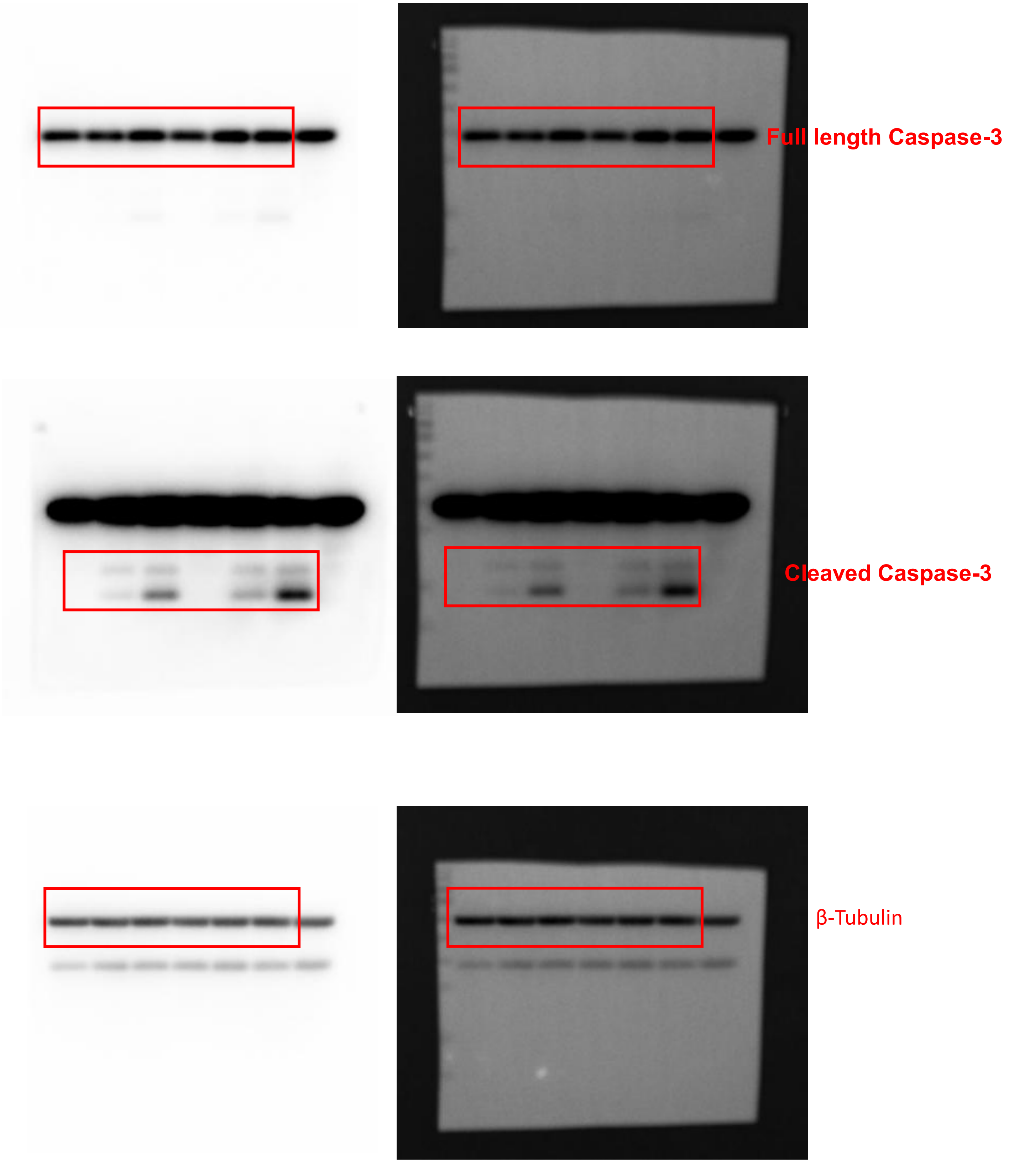

Figure S2E : Full length Lamin A/C; Cleaved Lamin A; Cleaved Lamin C、GAPDH

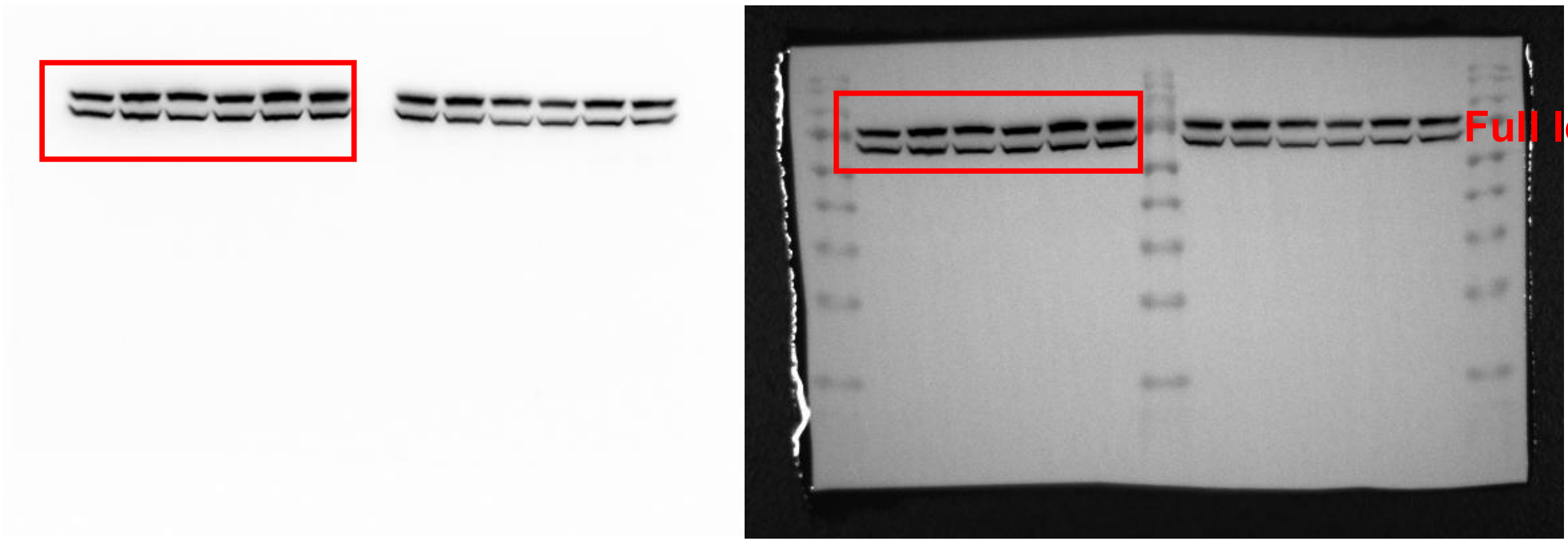

Full length Lamin A/C

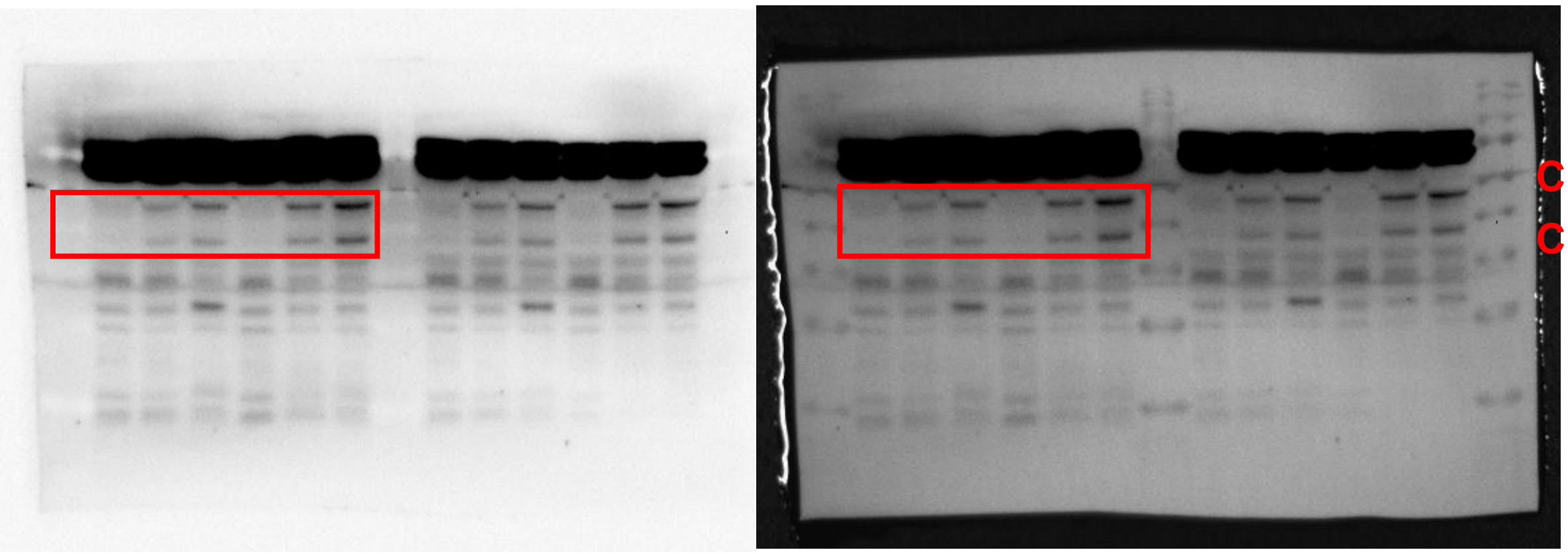

Cleaved Lamin A

Cleaved Lamin C

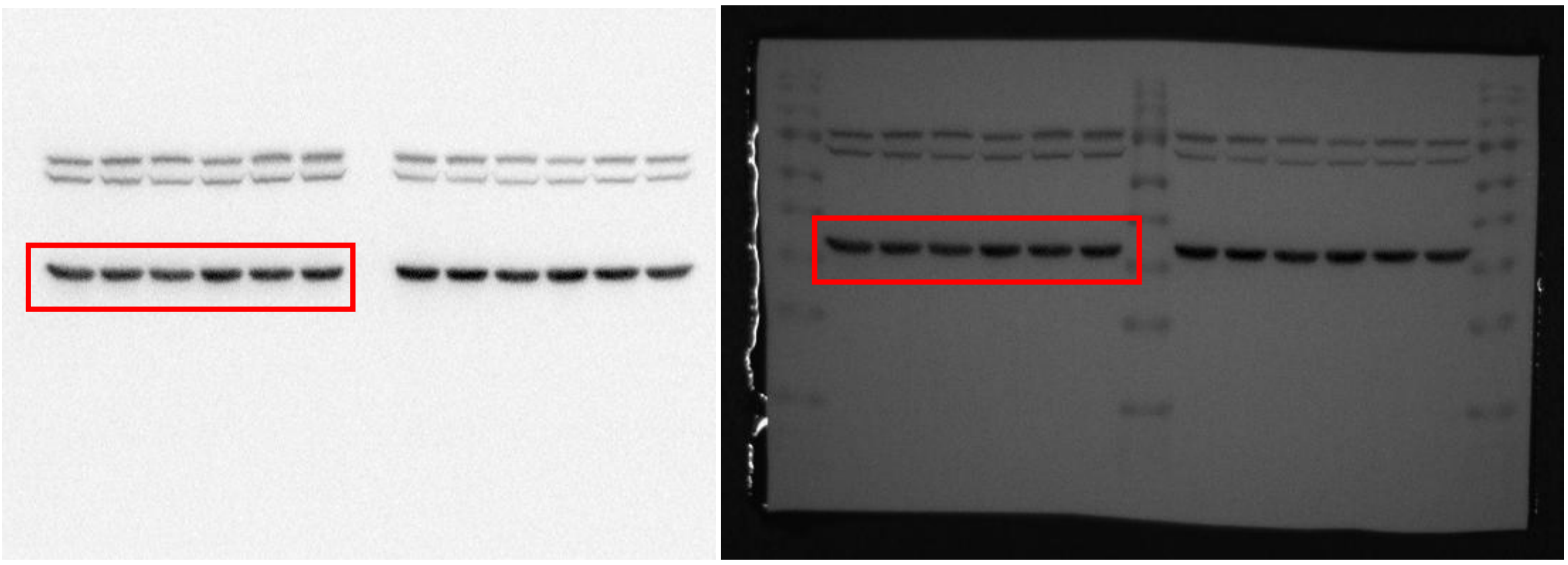

GAPDH

Figure S2E : Full length Caspase-3、Cleaved Caspase-3、β-actin

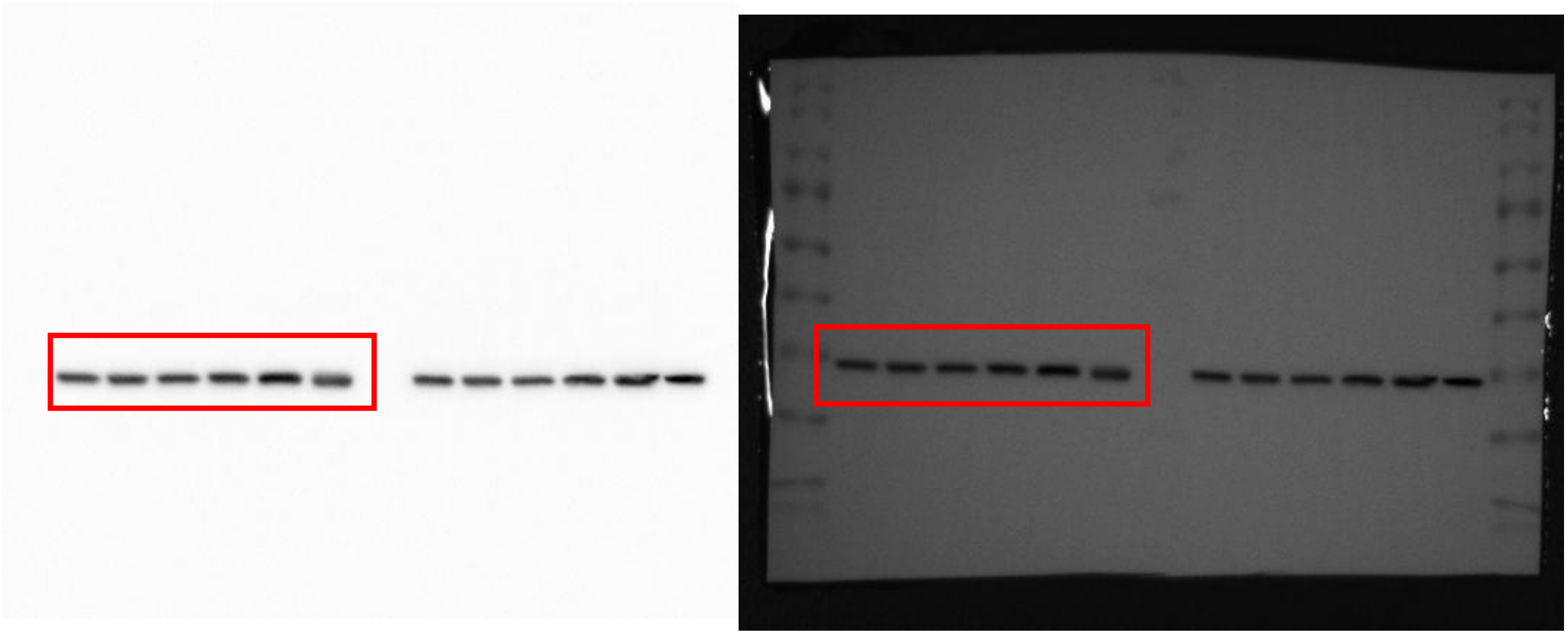

Full length Caspase-3

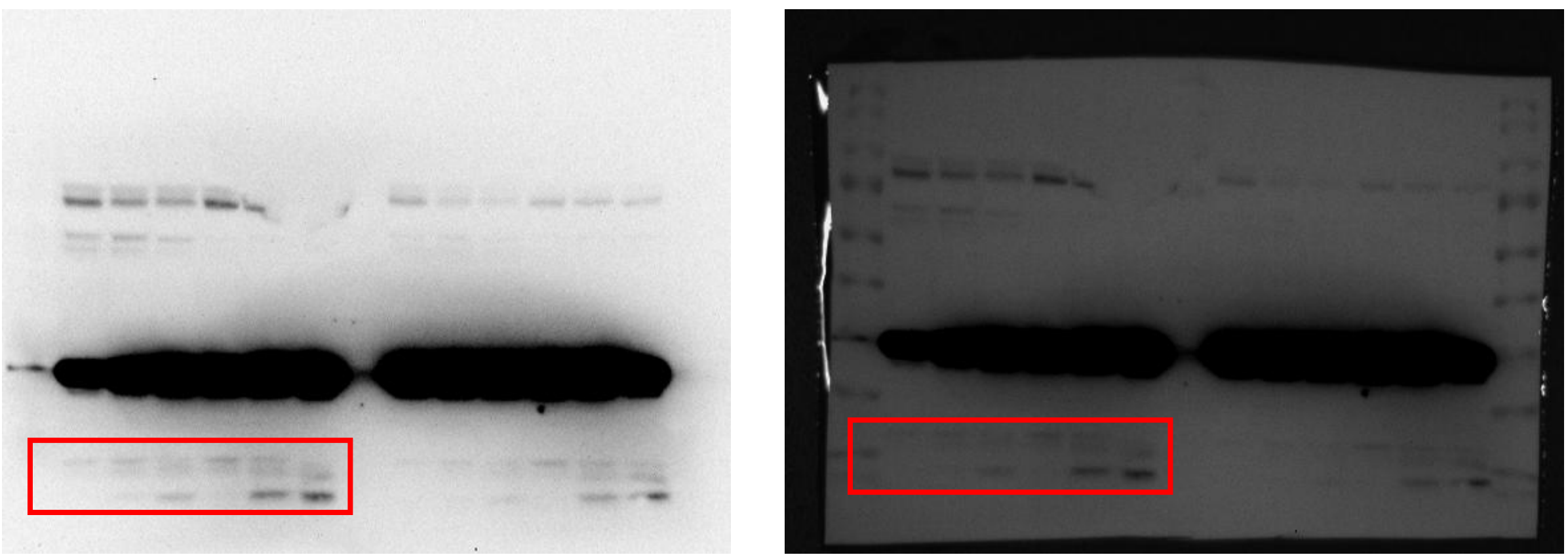

Cleaved Caspase-3

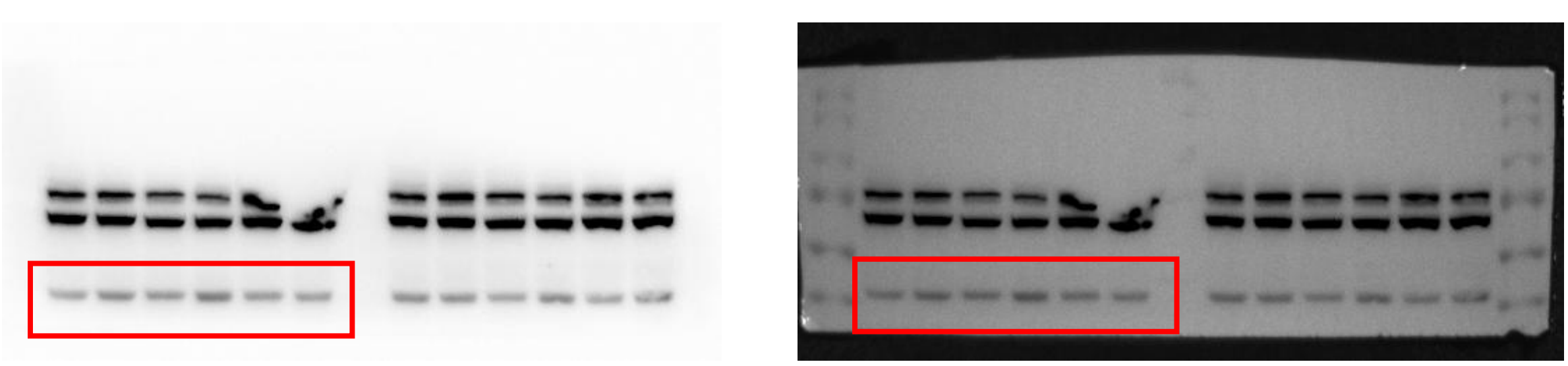

β-actin

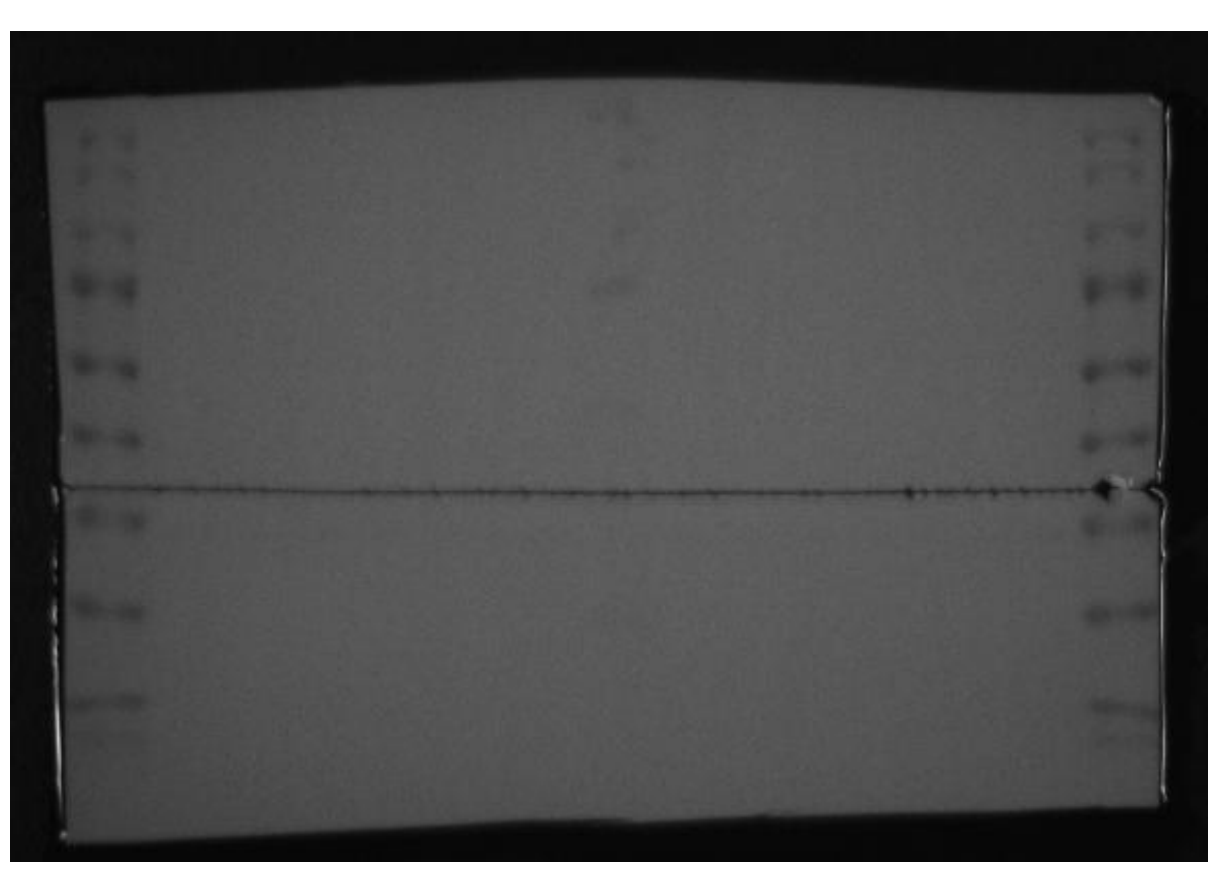

Figure S2E : Full length PARP、Cleaved PARP、β-Tubulin

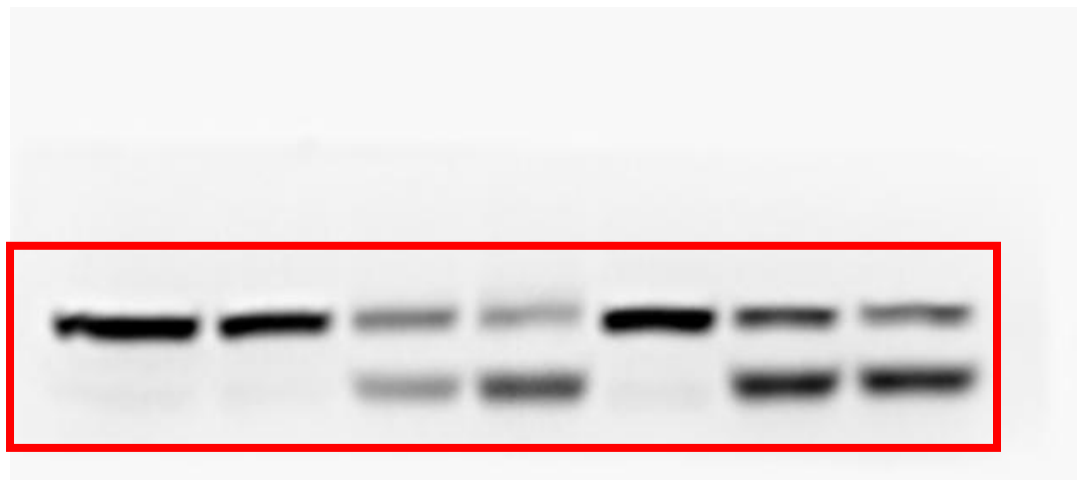

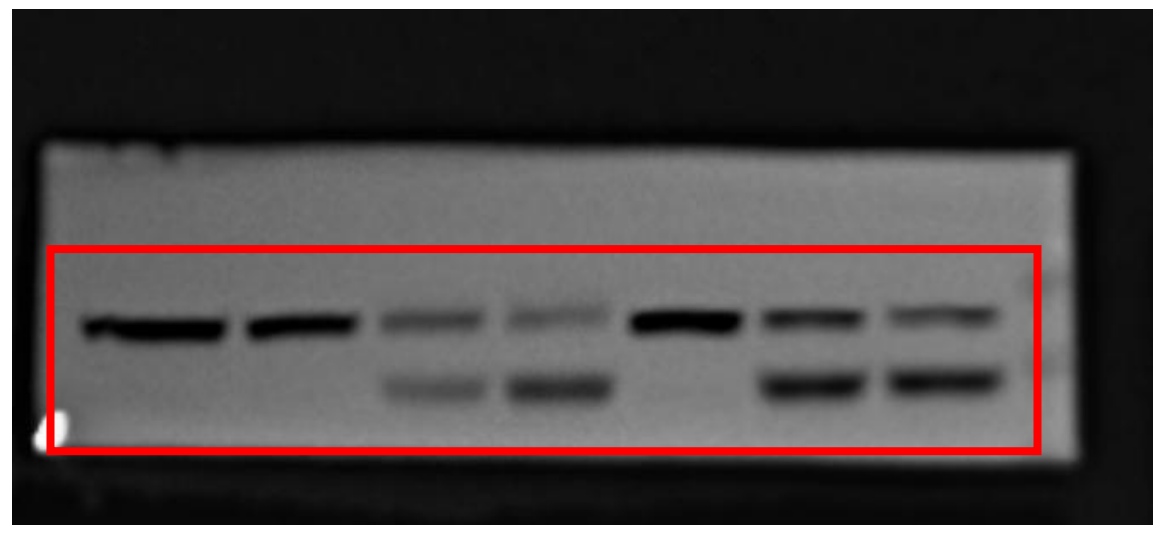

Full length PARP
Cleaved PARP

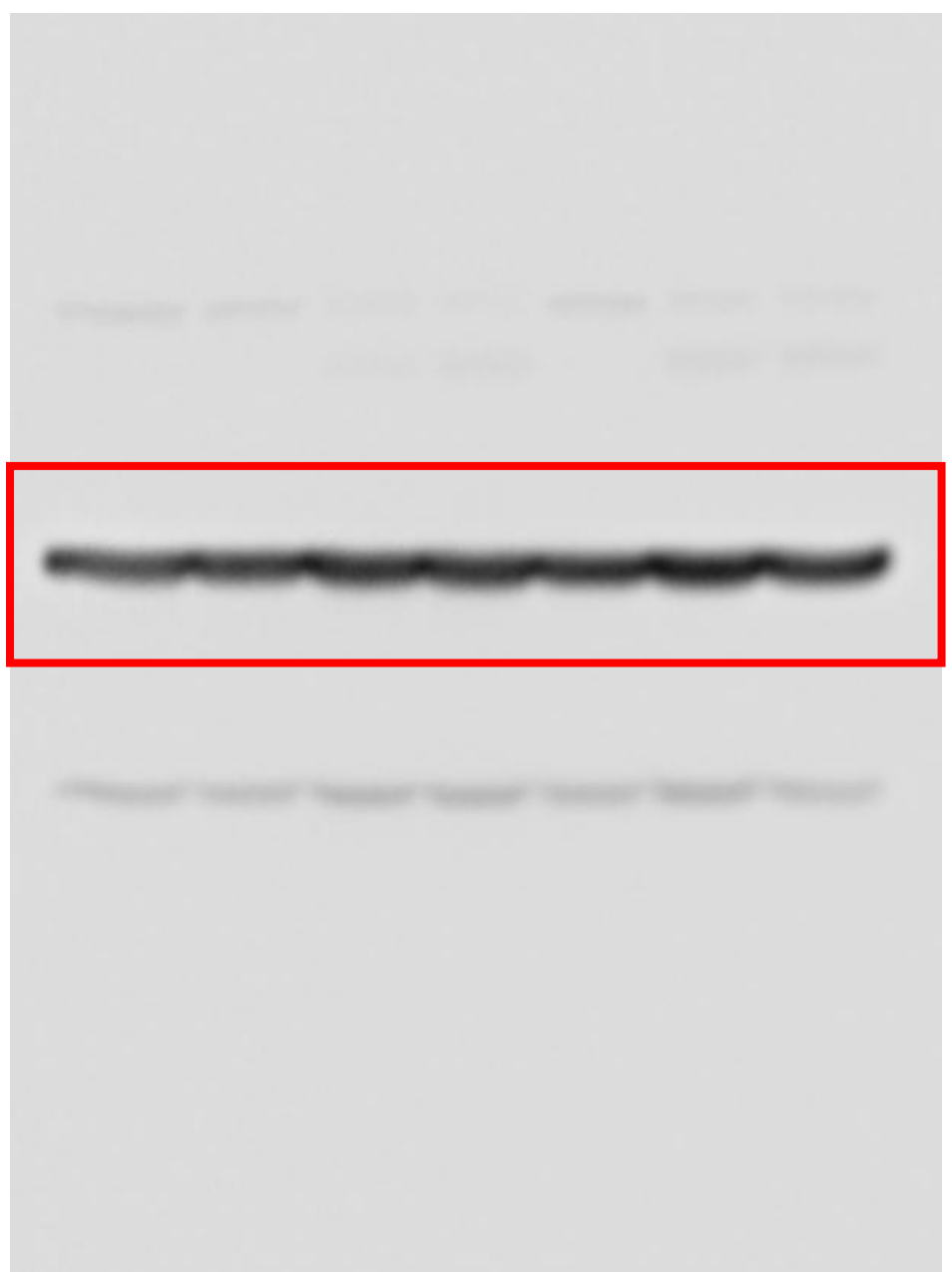

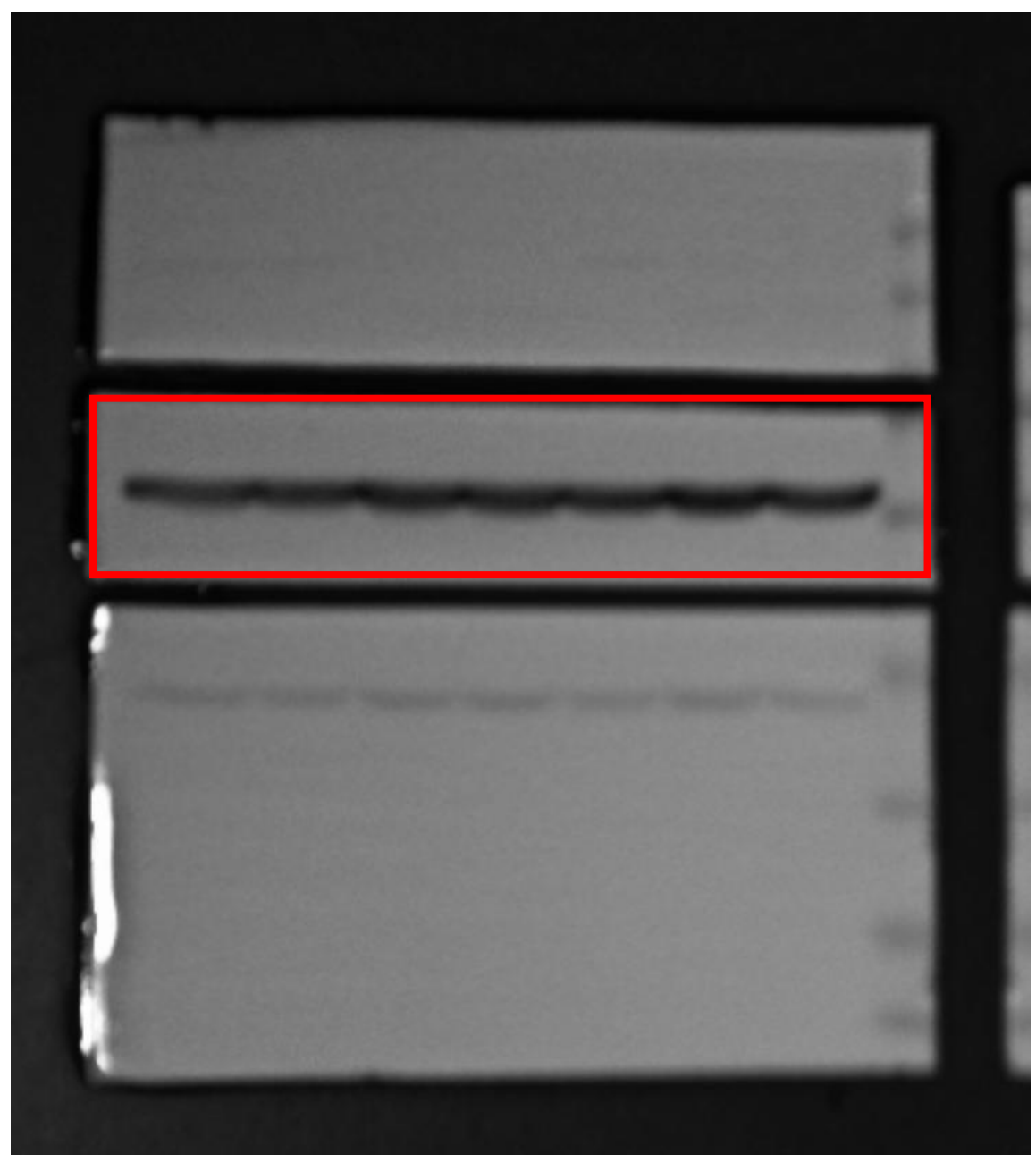

β-Tubulin

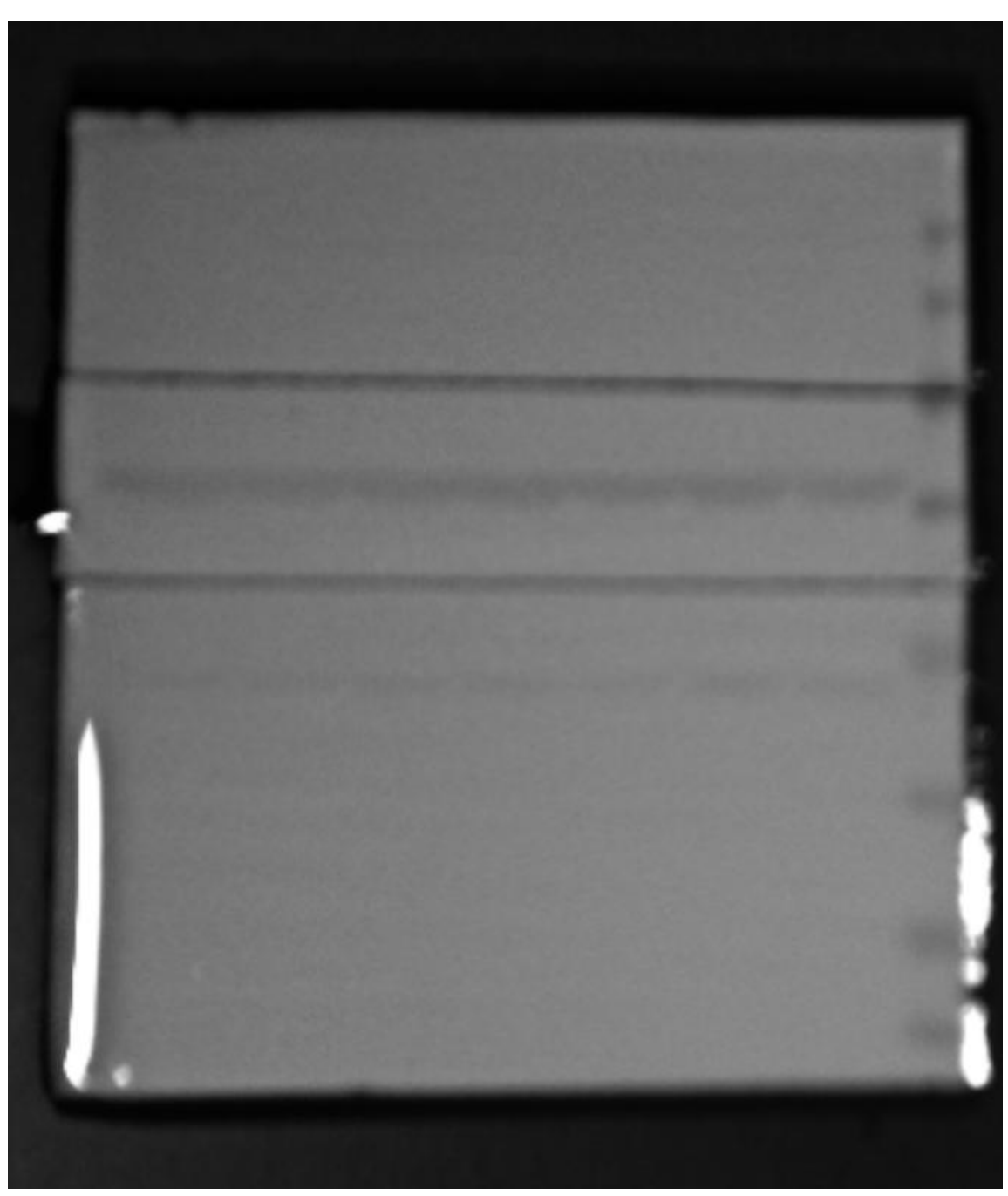

Figure S2E : Full length Caspase-8; Cleaved Caspase-8 p43/p41

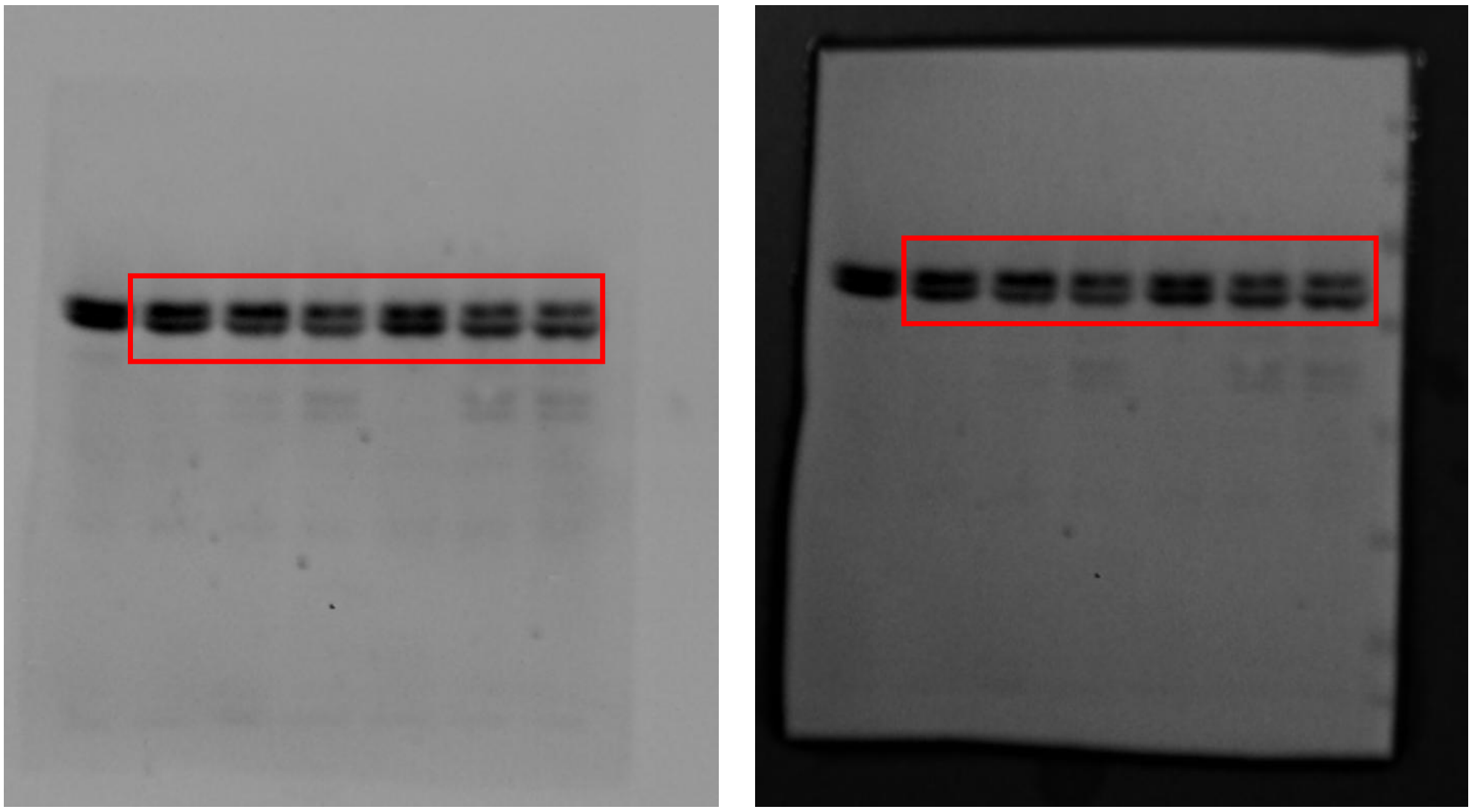

Full length Caspase-8

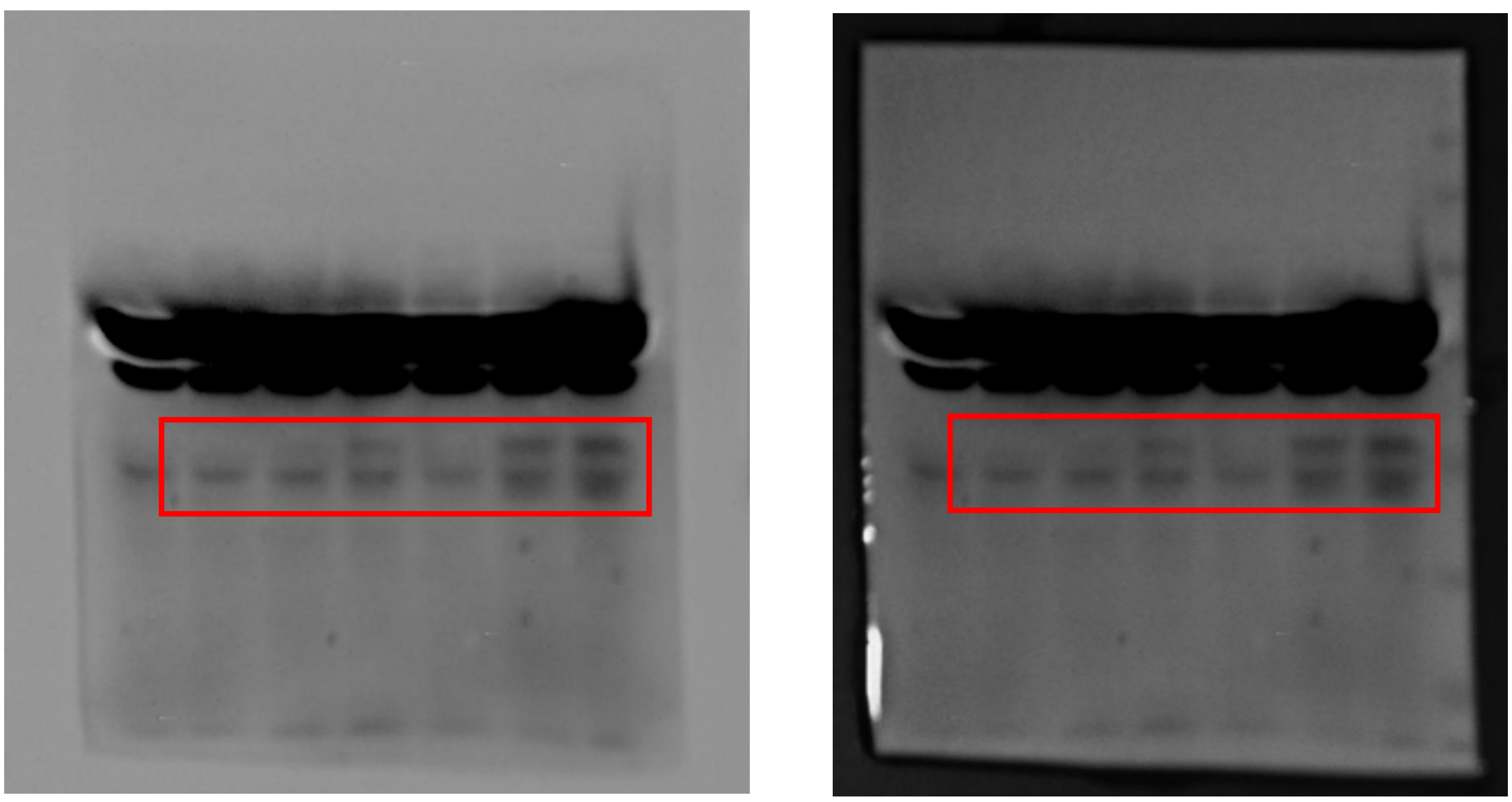

Cleaved Caspase-8 p43/p41

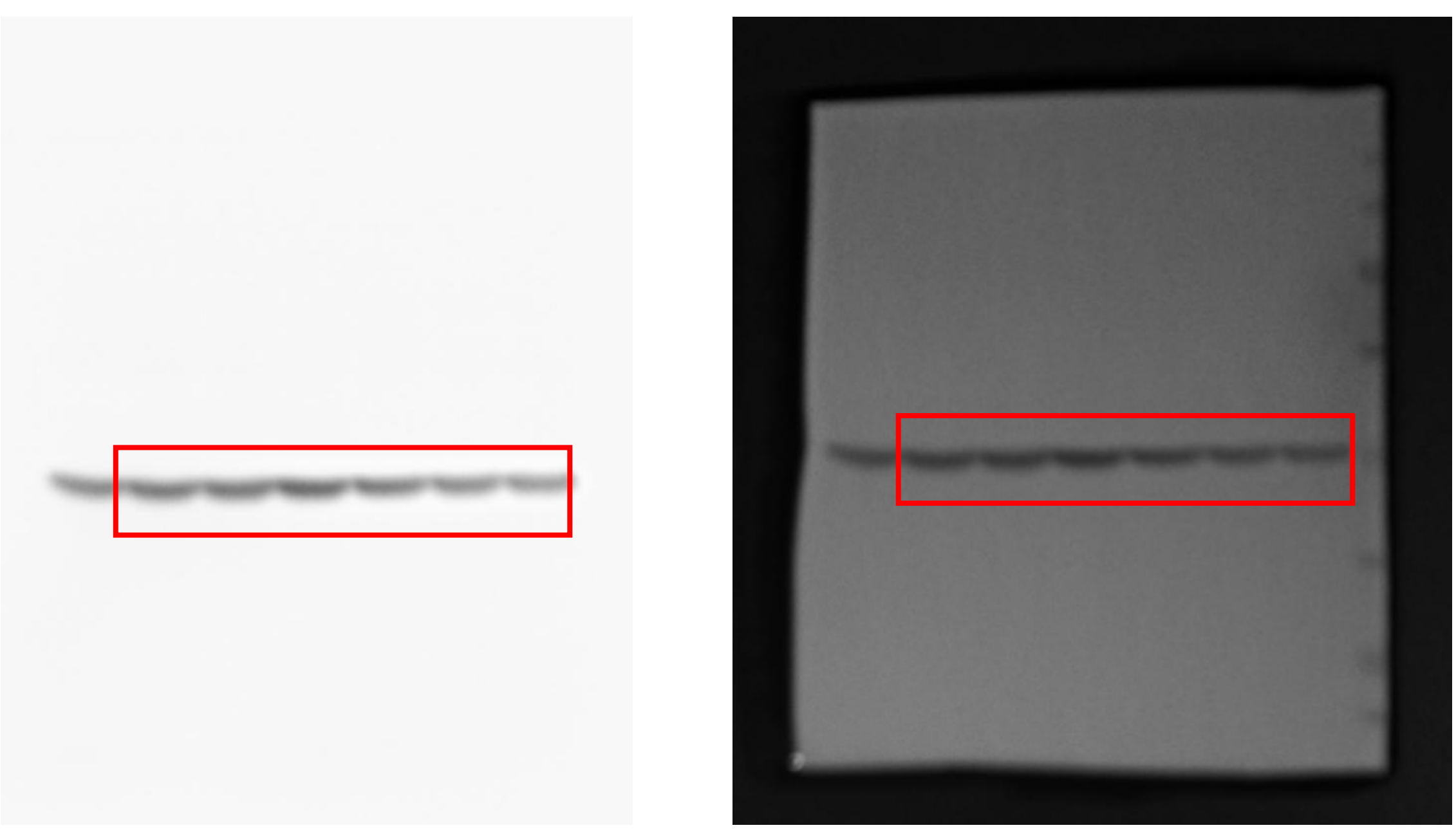

GAPDH

Figure S3A : STX17; β-Tubulin

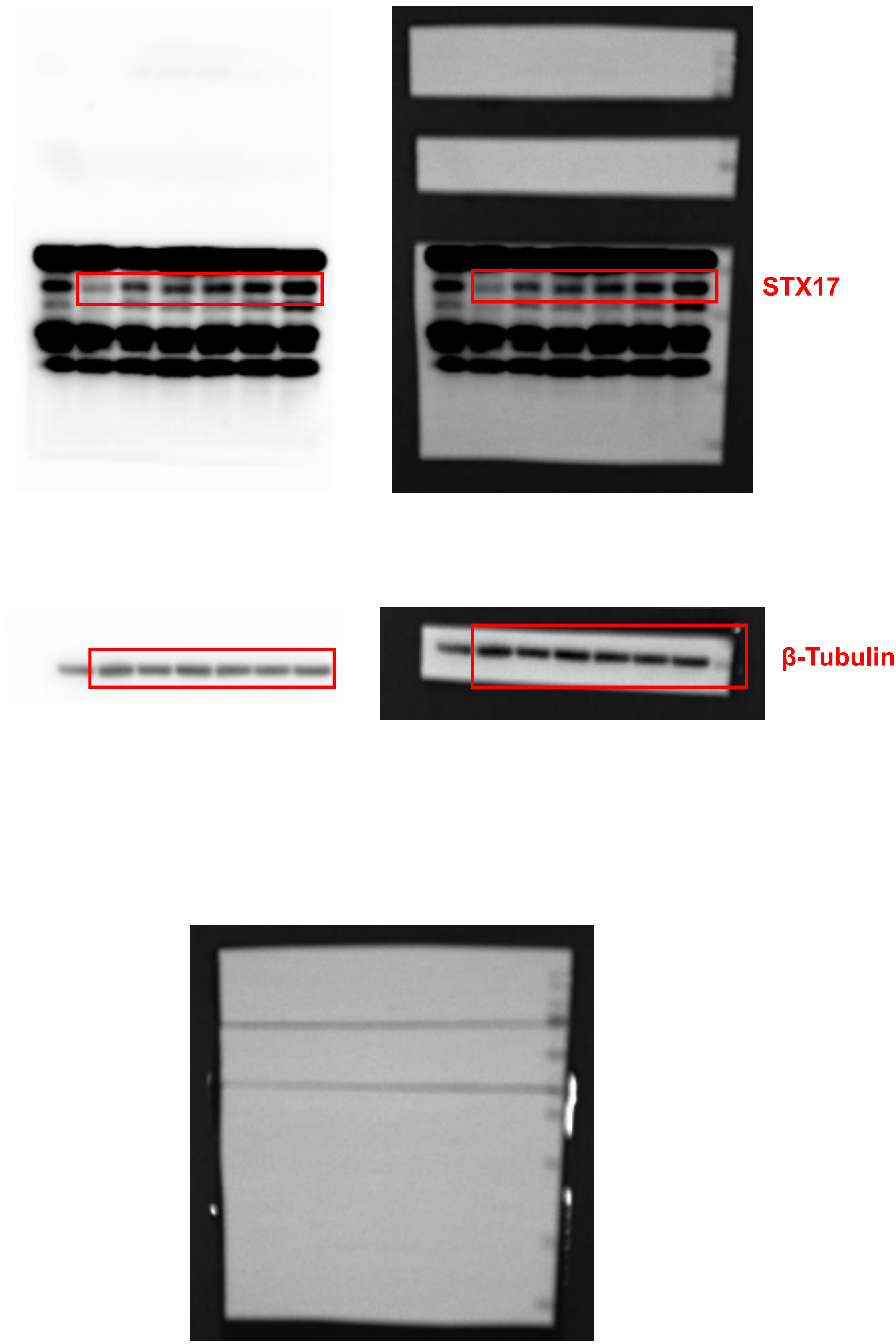

Figure S3A : UVRAG

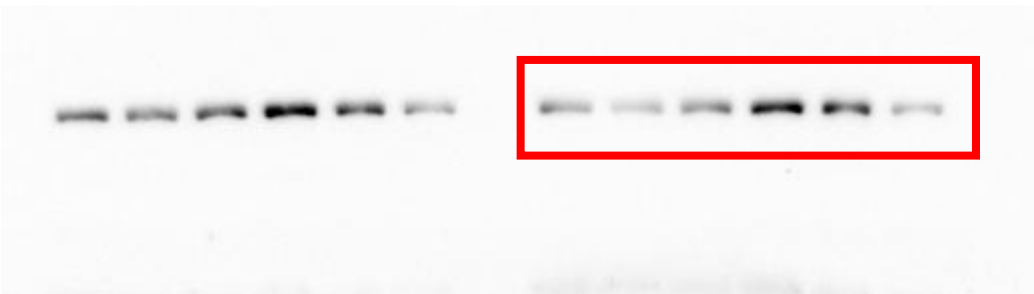

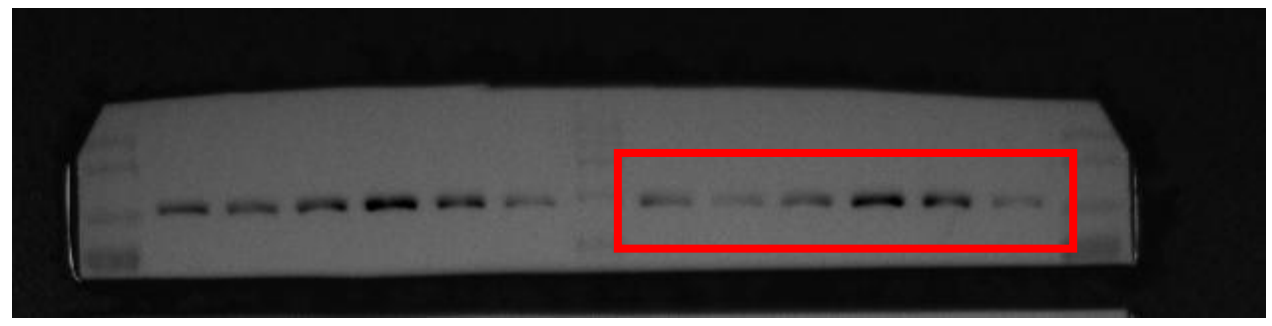

UVRAG

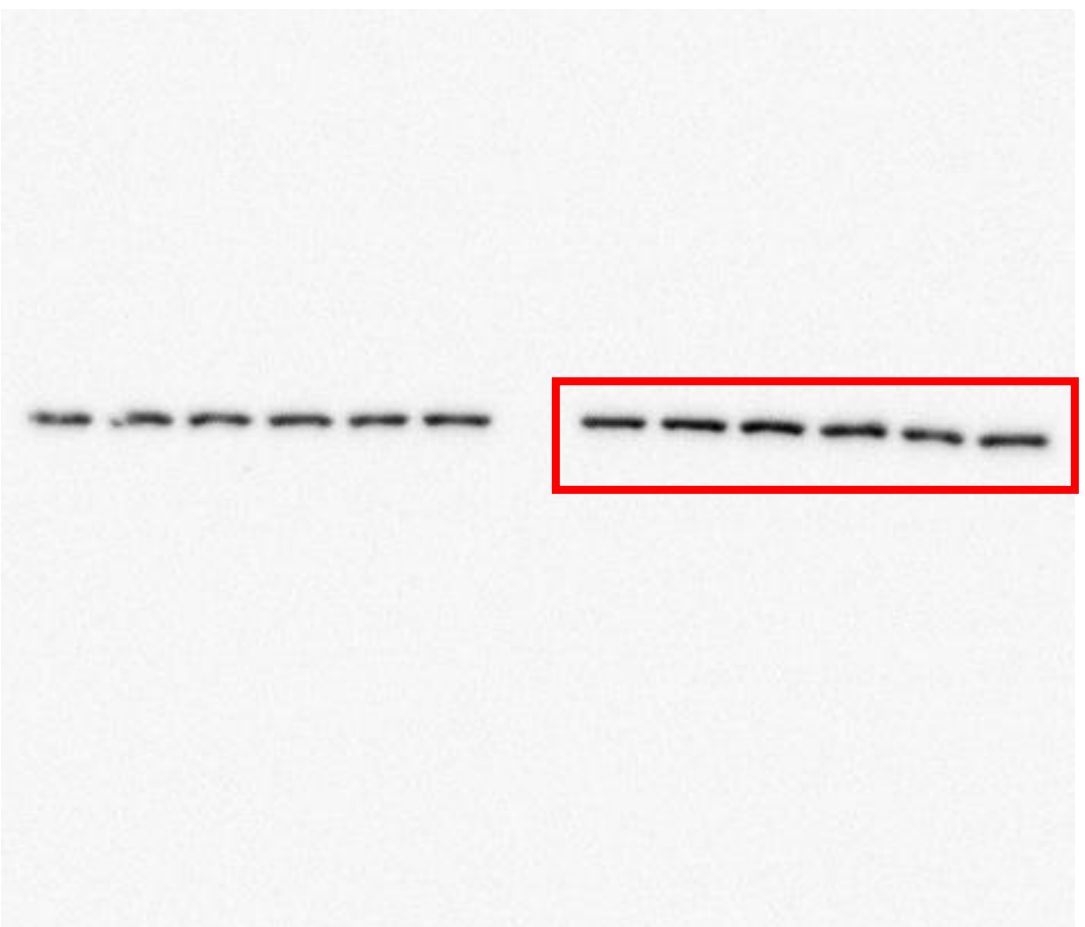

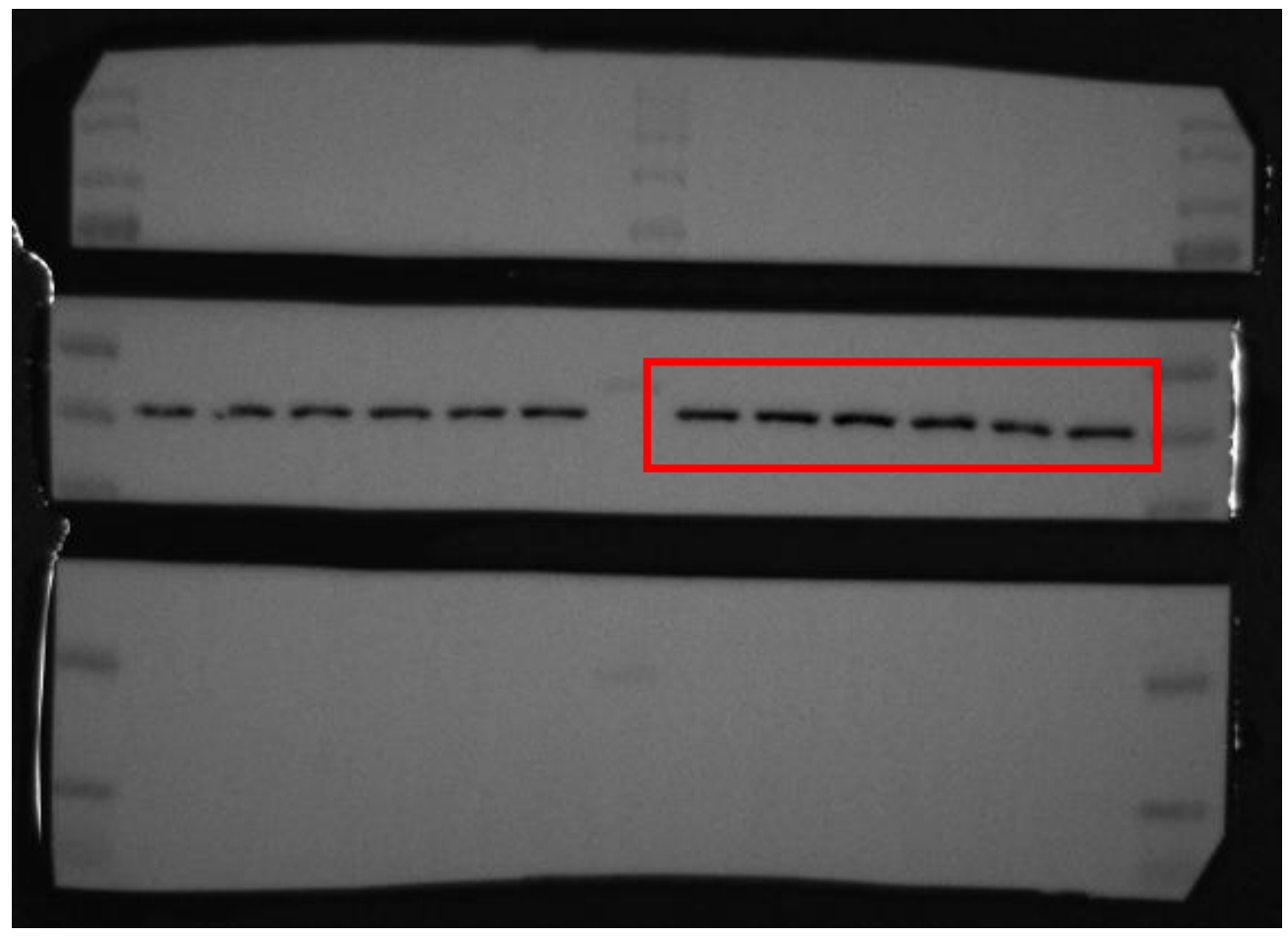

β-actin

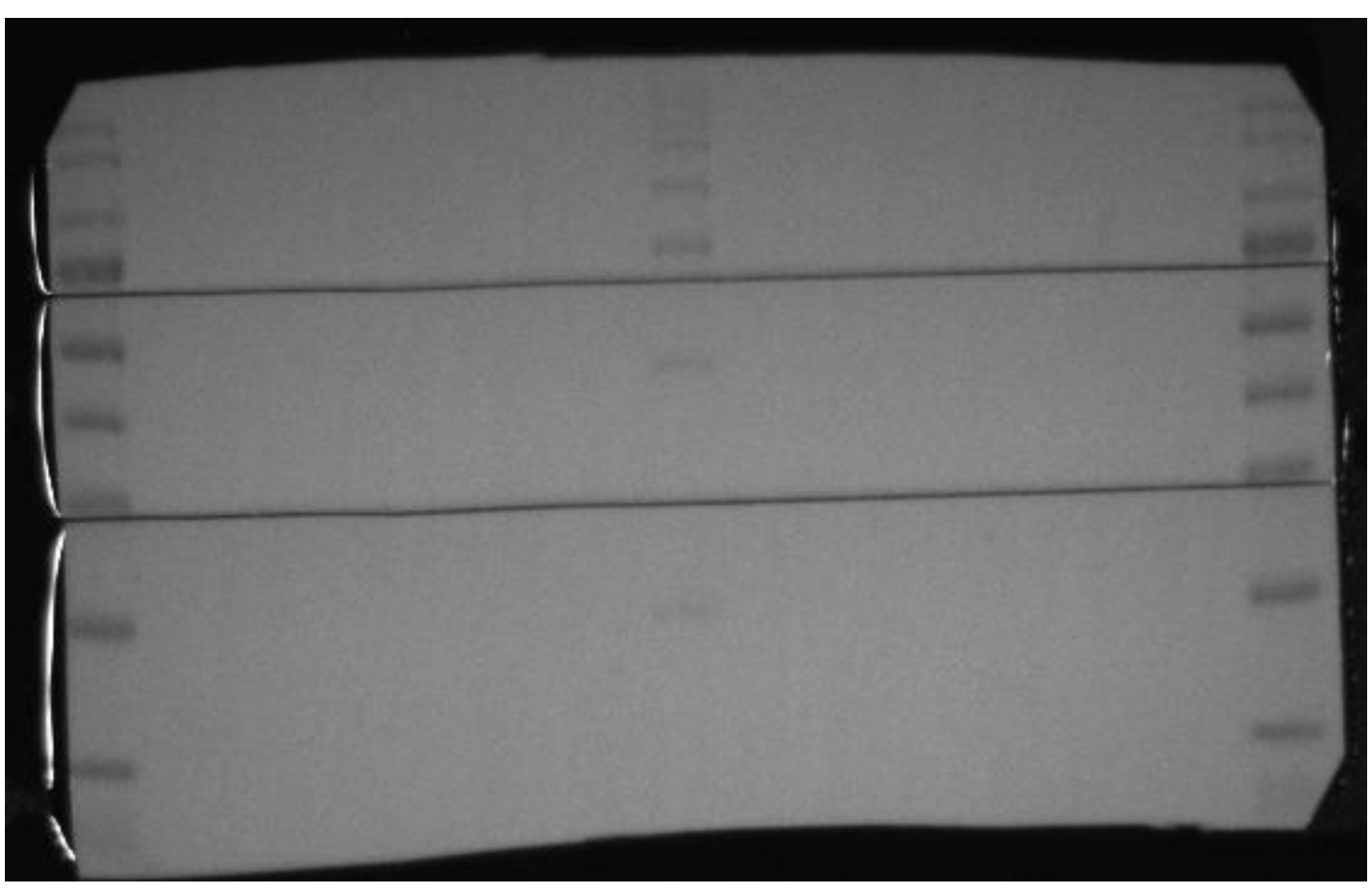

Figure S3A : RAB33B

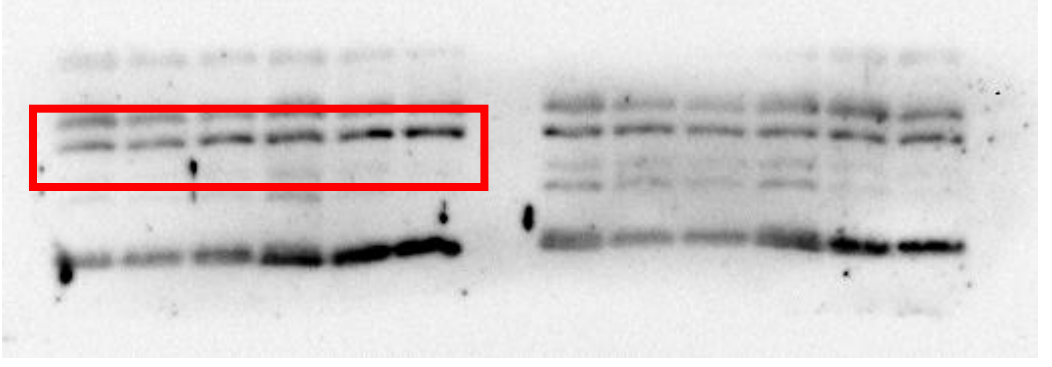
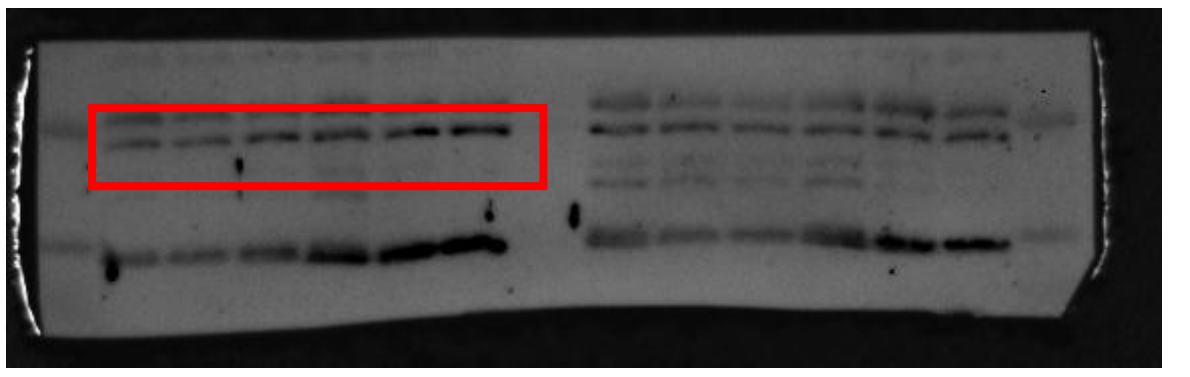

RAB33B

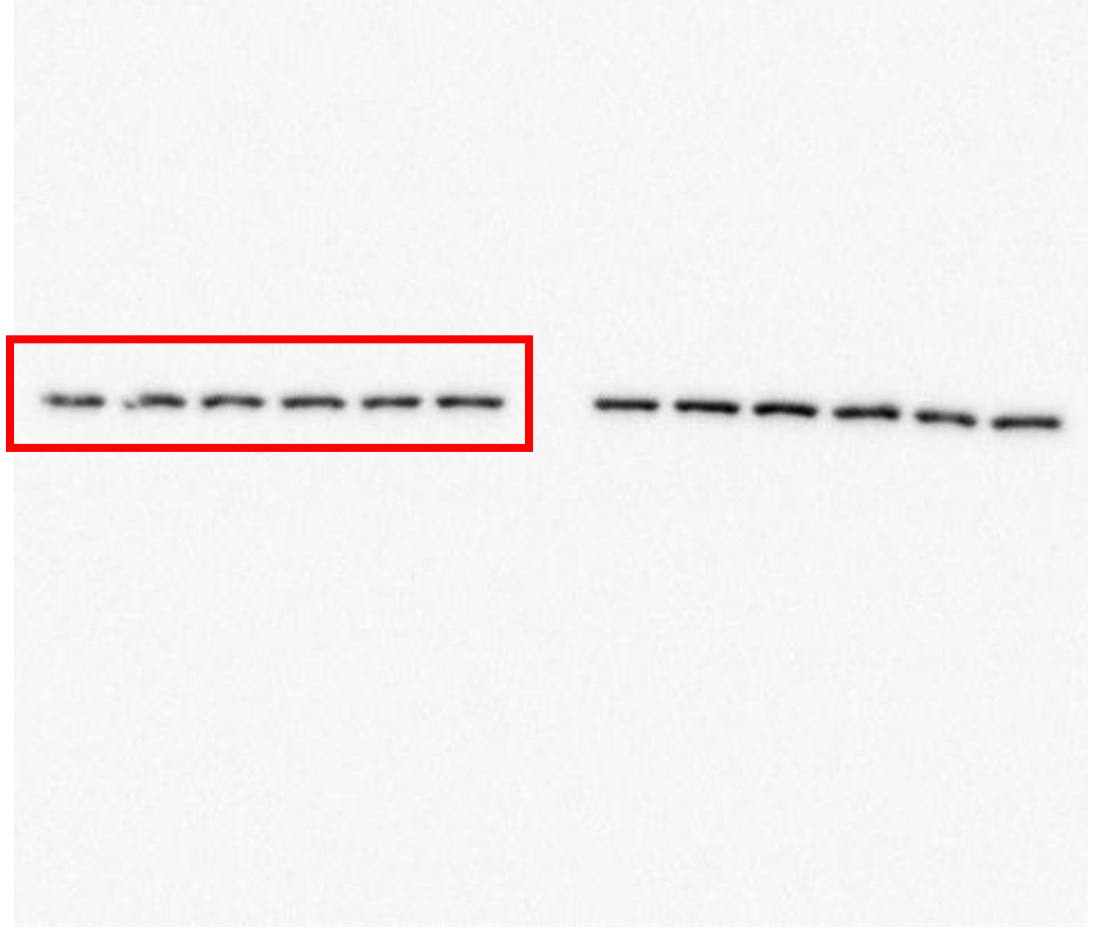
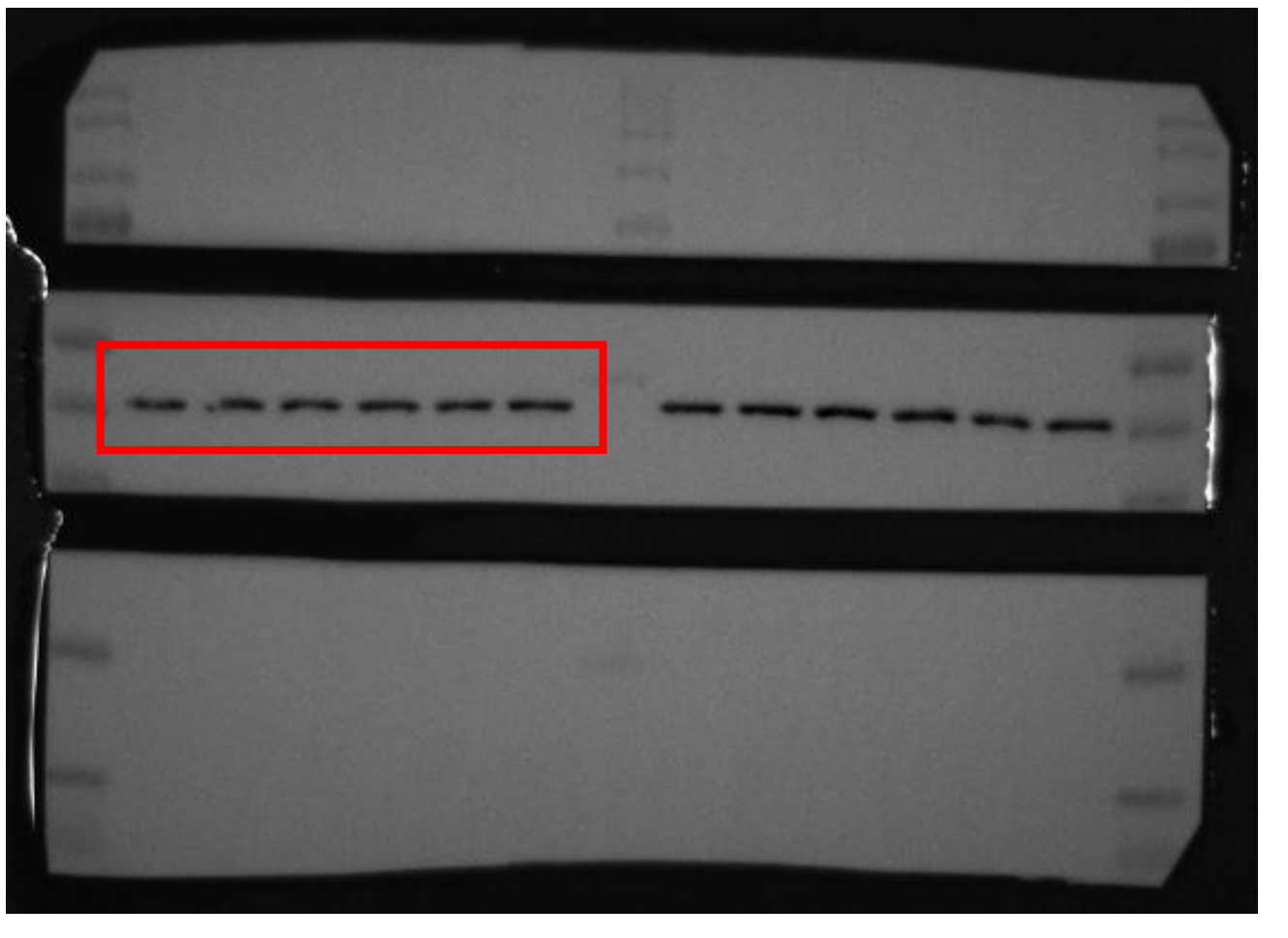

β-actin

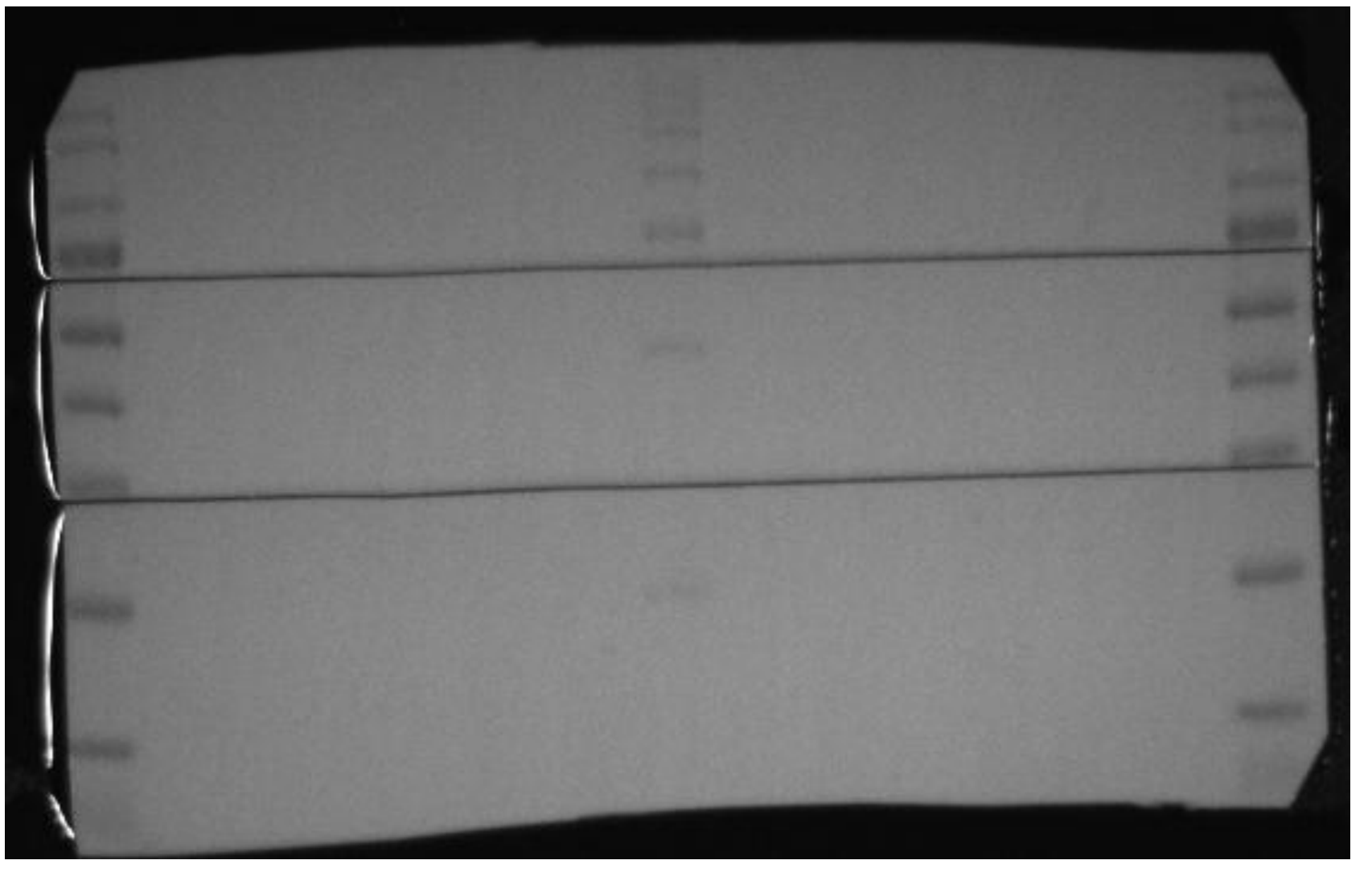

Supplement: Supplementary file 1 [file 13062_2026_909_MOESM7_ESM.pdf]
