# Supplementary material for: SLC7A11 suppresses paclitaxel resistance and improves survival via the KLF2 regulatory axis associated with autophagy in ovarian cancer
Source: Biol Direct. 2026 Jul 22;21:209. doi: 10.1186/s13062-026-00909-x (PMC13679285; doi:10.1186/s13062-026-00909-x)
Supplement: Supplementary file 2 [file 13062_2026_909_MOESM1_ESM.docx]

**Fig. S1** Effect of SLC7A11 overexpression on drug resistance in ovarian cancer paclitaxel-resistant cells (SKOV3-R, S-R). (**A-C**) The expression level of SLC7A11 in SLC7A11-overexpressed S-R-SLC7A11 cells and control S-R-EV cells was analyzed by RT-qPCR and Western blotting. The protein bands were quantified by Image J software. (**D, E**) S-R-SLC7A11 and S-R-EV cells were treated with the same concentration gradient of paclitaxel for 72 h, respectively. The cell viability and IC50 of paclitaxel were detected by CCK-8 assay. (**F**) Representative scan images of the colony formation assay. S-R-SLC7A11 and S-R-EV cells were treated with the gradient of paclitaxel for 13 days, respectively. Scale bar: 4 mm. (**G**) The relative colony formation rates of S-R-SLC7A11 and S-R-EV cells were analyzed by Image J. Data are presented as mean±SD. *, *P*<0.05; **, *P*<0.01; ***, *P*<0.001.

**Fig. S2** Effect of SLC7A11 overexpression on the expression levels of SLC7A11, cell cycle, and apoptosis proteins in paclitaxel-resistant ovarian cancer cells (HeyA8-R, H-R) and their parental cells (HeyA8, H) under paclitaxel treatment. (**A, B**) Western blotting was used to detect the expression of SLC7A11, p21 Waf1/Cip1, p27 Kip1, p18 INK4C, Cyclin B1, and Cyclin D1 in H-eGFP and H-SLC7A11 cells treated with the same concentration of paclitaxel for 72 h, and the protein bands were quantified by Image J software. **(C, D**) Western blotting was used to detect the expression of Full length Lamin A/C, Cleaved Lamin A, Cleaved Lamin C, Full length Caspase-3, and Cleaved Caspase-3 in H-eGFP and H-SLC7A11 cells treated with the same concentration of paclitaxel for 72 h, and the protein bands were quantified by Image J software. (**E, F**) Western blotting was used to detect the expression of Full length Lamin A/C、Cleaved Lamin A、Cleaved Lamin C、Full length Caspase-3、Cleaved Caspase-3、Full length Caspase-8、Cleaved Caspase-8、Full length PARP, and Cleaved PARP in H-R-eGFP and H-R-SLC7A11 cells treated with the same concentration of paclitaxel for 72 h, and the protein bands were quantified by Image J software. The expression levels of the same proteins in the two cell lines treated with the same concentration of paclitaxel for 72 h were statistically analyzed. Data are presented as mean±SD. *, *P*<0.05; **, *P*<0.01; ***, *P*<0.001.

**Fig. S3** Effect of SLC7A11 overexpression on the protein expression levels of STX17, UVRAG, and STX17 in paclitaxel-sensitive ovarian cancer cells (HeyA8, H) treated with paclitaxel. (**A, B**) The protein expression levels of STX17, UVRAG, and STX17 in H-EGFP and H-SLC7A11 cells treated with the same concentration of paclitaxel for 72 h were detected by Western blotting, and the protein bands were quantified by Image J software. The expression levels of the same proteins in the two cell lines treated with the same concentration of paclitaxel for 72 h were statistically analyzed. Data are presented as mean ± SD. *, *P*<0.05; **, *P*<0.01.

**Fig. S4** Sensitivity analysis of SLC7A11 prognostic value using the Receiver operating characteristic curve-derived optimal cutoff (immunohistochemical score = 3.375) in the ovarian cancer patient cohort. **(A)** Kaplan-Meier curve of OS in the 48-patient cohort with available OS follow-up data, with matched number-at-risk tables. *P* values by log-rank test. **(B)** Kaplan-Meier curve of PFS in the 57-patient cohort with documented recurrence records, with matched number-at-risk tables. *P* values by log-rank test. **(C)** Forest plot of multivariate Cox regression for OS in the 48-patient cohort, adjusted for SLC7A11 expression, HGSOC histology, NACT status, age, and postoperative chemotherapy cycles. PFS, progression-free survival. OS, overall survival. HGSOC, High-grade serous ovarian cancer. NACT, Neoadjuvant chemotherapy. HR, Hazard ratio. CI, Confidence interval.

**Fig S5** FDR-corrected analysis of the effect of SLC7A11 overexpression on protein expression profile in paclitaxel-resistant ovarian cancer cells (HeyA8-R, H-R) (**A**) Volcano plots of differential proteins after Benjamini-Hochberg FDR correction: H-R-SLC7A11+Control vs. H-R-eGFP+Control (top) and H-R-SLC7A11+PTX vs. H-R-eGFP+PTX (bottom). **(B)** Top 22 enriched KEGG pathways for both comparisons, with input number, Rich factor, raw P value, and Benjamini-Hochberg FDR value shown for each pathway. **(C)** Venn diagrams showing overlapping/unique down-regulated (left) and up-regulated (right) proteins between the two comparison groups, with representative overlapping proteins listed.
