# Supplementary material for: SLC7A11 suppresses paclitaxel resistance and improves survival via the KLF2 regulatory axis associated with autophagy in ovarian cancer
Source: Biol Direct. 2026 Jul 22;21:209. doi: 10.1186/s13062-026-00909-x (PMC13679285; doi:10.1186/s13062-026-00909-x)
Supplement: Supplementary file 3 [file 13062_2026_909_MOESM2_ESM.docx]

**Table S1. The clinical data of 65 patients with ovarian cancer**

| Clinical data parameters | Results | Number (%) |
| --- | --- | --- |
| Age (Year) ^a^ | >53 | 35 (53.85%) |
|  | ≤53 | 30 (46.15%) |
| Neoadjuvant chemotherapy | Yes | 23 (35.38%) |
|  | No | 42 (64.62%) |
| Postoperative chemotherapy ^b^ | >6 | 23 (35.38%) |
|  | ≤6 | 42 (64.62%) |
| High-grade serous ovarian cancer | Yes | 57 (87.69%) |
|  | No | 8 (12.31%) |
| Treatment status | Post-treatment recurrence | 57 (87.69%) |
|  | On-treatment progression | 6 (9.23%) |
|  | Primary resistance | 2 (3.08%) |
| Chemotherapy-sensitive | Yes | 35 (53.85%) |
|  | No | 30 (46.15%) |
| Successful patient follow-up | Yes | 48 (80.00%) |
|  | No | 17 (20.00%) |
| Death | Yes | 28 (49.23%) |
|  | No | 20 (30.77%) |

Note: ^a^ The age was divided into two groups according to the mean age of 53 years. ^b^ Postoperative chemotherapy was divided into two groups according to the average of 6 times of postoperative chemotherapy.

**Table S3. Primer sequence**

| Primer names | Primer sequence（5’-3’） |
| --- | --- |
| *SLC7A11* | Forward: TGCCCAGATATGCATCGTCC  Reverse: TCTTCTTCTGGTACAACTTCCAGT |
| *LC3B* | Forward: GAACGATACAAGGGTGAGAAGC  Reverse: TGAGCTGTAAGCGCCTTCTAAT |
| *KLF2* | Forward: TACACCAAGAGTTCGCATCTGA  Reverse: GCACAGATGGCACTGGAATG |
| *TFCP2* | Forward: CTGGCTTCAACAGTTCCCATAG  Reverse: CTGAGGTGTGGTTGTTGGTAAG |
| *SOX7* | Forward: CTCAGCAAGATGCTGGGAAAG  Reverse: ACTTGTAGTTGGGGTAGTCCTG |
| *GAPDH* | Forward: CAGCCTCAAGATCATCAGCAAT  Reverse: AGTCCTTCCACGATACCAAAGT |
